# Effectiveness of a culturally appropriate nutrition educational intervention delivered through health services to improve growth and complementary feeding of infants: A quasi-experimental study from Chandigarh, India

**DOI:** 10.1371/journal.pone.0229755

**Published:** 2020-03-17

**Authors:** Nikita Sharma, Madhu Gupta, Arun Kumar Aggarwal, Mutyalamma Gorle

**Affiliations:** 1 Department of Community Medicine and School of Public Health, Postgraduate Institute of Medical Education and Research (PGIMER), Chandigarh, India; 2 Dietetics Department, Postgraduate Institute of Medical Education and Research (PGIMER), Chandigarh, India; University of Ghana, GHANA

## Abstract

**Background:**

Malnutrition is a major public health problem in India, especially among urban poor children. The objective of the study was to determine the effectiveness of a culturally appropriate nutrition educational intervention that can be delivered through health services and digitized child undernutrition tracking module for health workers to improve complementary feeding of infants of age six months to 12 months in Chandigarh, North India, to prevent malnutrition in infants.

**Methods:**

A quasi-experimental study was conducted in a non-randomized intervention (Burail) and control area (Maloya) among a vulnerable population in Chandigarh, North India. The mother-infant dyads (MIDs) in the intervention group(n = 202) received culturally appropriate nutrition educational intervention, were supported individually by trained health workers in infant feeding and followed up for six months. Health workers were monitored through a digitized tracking module. The MIDs in the control group (n = 202) received routine care under the national health program. The mean (±S.D.) age of infants in the intervention and control group was 5.4 (±0.8) months and 5.5 (±0.7) months, respectively. The data was collected using a pre-tested semi-structured questionnaire and anthropometry of infants at baseline and end line. The primary outcome was a mean change in weight. The effectiveness of the intervention was measured by conducting the difference in difference (DID) analysis in mean change in weight between intervention and control group.

**Result:**

At baseline, the mean (±S.D.) weight of infants was 6.6(±0.64) kg and 6.6 (±0.52) kg in the intervention and control group. The mean (±S.D.) length of infants was 64.3 (±2.0) cm in the intervention group and 65.1 (±1.7) cm in the control group. Out of 404, 190 and 191 MIDs in the intervention and control group completed the study, respectively. A significantly higher number of infants in the intervention group were started on complementary feeding at six months of age (72.6% versus45.5%, p<0.01) and received foods having thick consistency (82.1% versus 41.9%, p<-0.01). There was significant weight gain in intervention group infants (DID means = 0.27 kg, p<0.01) and length gain (DID means = 0.9 cm, p<0.01) from the baseline. Also, there was significant decline in the proportion of undernourished (10% versus18.8%, OR = 0.47, p = 0.01) and wasted infants (7.3% versus15.7%, OR = 0.42, p = 0.01) in the intervention group.

**Conclusion:**

Community-based nutrition educational intervention delivered through the routine health services and digitized tracking of malnourished children can effectively improve the complementary feeding and growth of children six months to one year among vulnerable populations.

## Introduction

Undernutrition is the underlying cause of nearly half of the global deaths of under-five children [1]. Globally in 2016, 155 million under-five children were estimated to be stunted, while 52 million were estimated to be wasted [2]. Investing in nutrition is considered a cost-effective intervention that will benefit both present and future generations [3]. More than a third of all under-five deaths occur only in two countries India(21%) and Nigeria (13%) [1]. In India, about 38.4% of under-five children are stunted, 35.7% of children are wasted and 21% of children are underweight as per a recent national family health survey report (round 4, 2015–16) [4]. Complementary feeding is initiated at the age of six to eight months in 42.7% of infants. But only 9.6% of the six to twenty-four months old young children receive an adequate diet [4]. In spite of the implementation of large scale supplementary feeding programs, like integrated child development scheme (ICDS), and strategies to combat malnutrition like integrated management of neonatal and childhood illness (IMNCI), the evidence of their impact on reducing child malnutrition is limited in India [5,6]. The nutrition education regarding complementary feeding is provided during village health nutrition days through auxiliary nursing midwife (ANM), through village health nutrition and sanitation committees, however a study conducted in Orissa and Jharkhandreported that these workers focused mainly on sanitation, record keeping and referral activities [7]. The guidelines for enhancing optimal infant and young child feeding practices were introduced in 2013 [8], and *Anganwadi* workers were trained, but they failed to impart sufficient knowledge regarding feeding practices to the caregivers or mothers [9].

Globally, commitment to improving nutrition is the second Sustainable Development Goal of achieving zero hunger [10]. The reduction of stunting among children under five years of age has been proposed in Indian National Health Policy 2017, under the cross-sectoral goals [11]. Under these circumstances, there is a renewed focus on effective strategies to reduce child undernutrition [12]. In a review of studies on interventions for child undernutrition and survival, it is observed that promotion of breastfeeding, behavior change communication to improve the complementary feeding, zinc supplementation, vitamin A fortification, handwashing and treatment of severe acute malnutrition are effective strategies to reduce child undernutrition, while other interventions like vitamin D supplements, iodine supplements, cooking in iron pots, preschool feeding programs, and growth monitoring showed little effect [12]. Existing nutrition educational studies to prevent malnutrition had intervened through the involvement and training of community workers, who then counseled the caregivers regarding the feeding of the children [13–16]. But, most of these studies have reported significant improvement in growth and feeding practices mainly in rural settings. Only a few such studies are available, that are conducted in peri-urban settings or among migrants [13]. Rural population include those living in the villages with no municipal board, and peri-urban or migrants in the urban areas are those who have migrated from their place of birth to the urban areas, but because they could not afford the cost of living in the urban areas, are staying either in the slums or peri-urban localities with problem of overcrowding, poor access to health system, safe water, and sanitation facilities as compared to urban areas [17]. Almost 91.6% of children living in rural areas have access to A*nganwadi* centers compared to 53.3% urban poor children in India [18]. Rural women had more contact with health workers than women living in slums. Another issue that needs to be considered is the sustainability of such efforts in the community and the integration of such interventions with the existing health services. Also, it is essential to consider the factors that will enhance the accessibility and affordability of quality home foods to the children especially those belonging to vulnerable groups of the society. This study is planned to determine the effectiveness of a culturally appropriate nutrition educational intervention that can be delivered through health services, and digitized child undernutrition tracking module for health workers to improve complementary feeding of infants of age six months to 12 months in Chandigarh, North India, to prevent malnutrition in infants.

## Material and methods

The ethical approval to conduct this study was obtained from the Institutional Ethics Committee of PGIMER, Chandigarh (INT/IEC/2017/758) on 15^th^ June 2017. This intervention study was registered with the clinical trials registry, India (CTRI/2018/03/012512) retrospectively, as before April 2018, both prospective and retrospective registration was allowed by CTRI. The authors confirmed that all ongoing and related trials for this drug/intervention are registered. The study protocol is given as S1 File. There is no change in the methodology from the study protocol, except that we had planned to enroll mother-infant dyads and all the available health workers including auxiliary nurse midwives and *Anganwadi* workers in the study area. However, permission to involve the A*nganwadi* workers could not be obtained from Women and Child Development, and *Anganwadi* workers did not give consent to participate in this study, hence they were excluded. But they gave consent to use the *Anganwadi* centers for delivery of the intervention. Since the sample size estimations were not based on the number of *Anganwadi* workers, hence their exclusion in the study did not affect the internal validity of the study.

### Study area and study design

**Fig 1:**
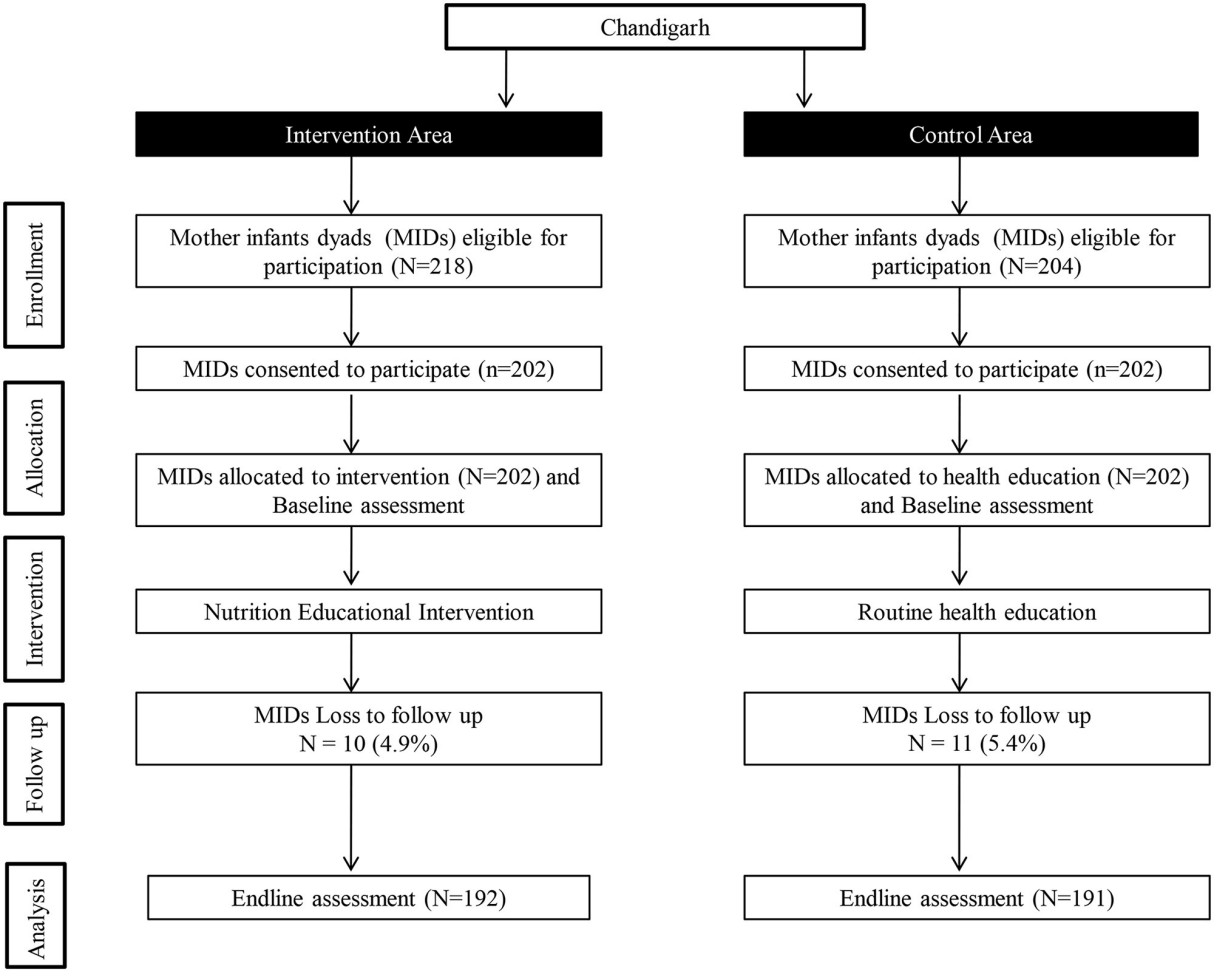
Flow chart showing the study design.

This study was conducted in Chandigarh, a Union territory located in north India. About 97% of the population resides in the urban area as per census 2011 [19]. Out of which, about 30% of the population resided in resettlement colonies. The study design was quasi-experimental. The flow chart of the study design is given as Fig 1.

**Fig 2:**
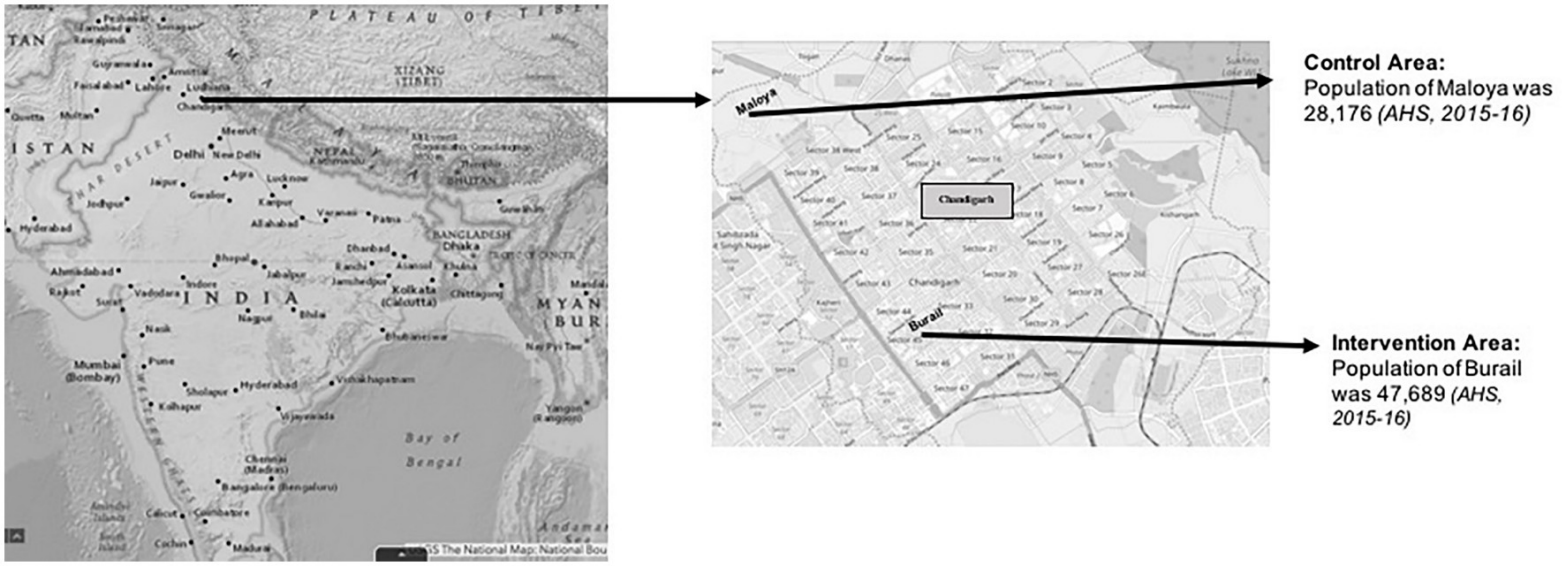
Map of the study area.

The intervention (Burail) and control area (Maloya) were selected purposively because the success of the intervention was dependent on the cooperation of ANMs and their medical officer-in-charge and also their willingness to participate in the study (Fig 2) [20].

The ANMs in both areas had agreed to participate in study. Burail (intervention area) was one of the field practice area of the Department of Community Medicine and School of Public health, PGIMER. PGIMER team was working in collaboration with the Chandigarh Administration in this area. The background characteristics of both areas (intervention and control) were similar in terms of vulnerability, migration of population, geographical location, socioeconomic status and baseline child health status. The utilization of child health services in Maloya (control area) was similar to Burail (intervention area) as indicated by the proportion of children receiving full immunization (90% vs. 97%). The distance between both the study areas was 9 km with a rare instance of inter-migration of the population between them.

The population of Burail (intervention area) was 47,869 with 862 infants below one year of age, as per the annual health survey report, 2015–16. Most of the residents in this area are migrants from neighboring states like Uttar Pradesh, Bihar, Uttarakhand, Haryana, and Himachal Pradesh, and belong to low or middle socioeconomic groups. About 97% of children aged between 12 to 23 months in Burail were fully immunized, as per the annual health survey report, 2015–16. There were six auxiliary nurse midwives (ANMs) and 22 *Anganwadi* workers (AWWs) and 22 *Anganwadi* centers in Burail. *Anganwadi* workers are the community level female health workers belonging to the area and working in *Anganwadi’s*. *Anganwadis* are the centers for providing supplementary nutrition, growth monitoring, and non-formal education to children less than six years old through *Anganwadi* workers [21]. Ministry of Women and Child Development is running these centers. Other field staff including ANMs and ASHAs (accredited social health activists) are under the Ministry of Health and Family Welfare, Government of India. ASHA works as a volunteer healthcare facilitator and mobilizes the community for health care utilization [22]. She conducts home visits of mothers and counsels them about breastfeeding and complementary feeding. No ASHA was working at both study areas under the National Health Mission in Chandigarh at the time of the study.

Maloya (control area) had a population of 28,176 with 483 infants below one year of age as per the annual health survey report, 2015–16, of Civil Dispensary. About 90% of children aged 12 to 23 months were fully immunized in this area as per the annual health survey 2015–16. There were four ANMs, one ASHA, and 26 AWWs and 26 *Anganwadi* centers in Maloya.

The ratio of ANMs and AWWs per 10,000 population was 1.25 and 5 respectively, in the intervention area; and 1.42 and 9.2 respectively, in the control area.

### Study population

The study population was mother-infant dyad (MIDs) with an infant in the age group of 4–6 months at enrollment, ANMs, and AWWs in the intervention and control area. The participants who were residing in the study areas for the past six months and had no plans to migrate during the intervention period were included in the study. The infants who had cerebral palsy, other congenital anomalies/malformations, severe acute malnutrition and suffering from severe illness were excluded from the study.

The permission for the involvement of AWWs in the study could not be obtained from the Department of Women and Child Development, Chandigarh. AWWs did not give consent to be the part of the study, as these workers had a low salary and feared that they would have to do extra work without monetary compensation if they were trained in providing nutrition education intervention by the researchers. Hence, these workers could not be included in this study.

### Sample size and sampling technique

The sample size for MID was calculated by using the formula $=\frac{{\left(u+v\right)}^{2}{(\sigma_{1}}^{2}+{\sigma_{0}}^{2})}{(\mu_{1}-\mu_{0})2}$ [23], where, u is two-sided percentage point of normal distribution corresponding to 100%—power = 1.28; v is percentage point of normal distribution corresponding to (two-sided) significance level = 1.96, assuming 80% power with 95% confidence interval; *μ*_1_-*μ*_0_ = difference between the means and assumed to be 250g [14], *σ*_1_ is the standard deviation in the intervention arm and assumed to be = 0.65; *σ*_0_ is the standard deviation in the control arm and assumed to be 0.65 [14]. Considering the attrition rate of 10%, the sample size for the study was calculated as 202 mother-infant dyads per arm. It was required to detect the difference in mean weight of 250 g between the intervention and control group infants [14].

A list of all MIDs, where the age of infants was between 4 to 6 months, was obtained from the *Anganwadi* centers of the study and control area. They were selected by simple random sampling from the complete list using computer-generated random numbers. Final inclusion was done for those MIDs that met the study criteria and gave consent for participation in the study. Written informed consent (S3 File) was obtained from all the eligible MIDs after giving them the participant information sheet (S4 File). In the first month of enrolment, all MIDs with age between four to six months were enrolled from 16^th^ June 2017. Further enrollment was continued thereafter until the required sample size was achieved in both the groups. All the ANMs in the intervention and control area were included.

The study was conducted in four phases from July 2016 to December 2017, which are described below.

### 1. Pre-intervention phase

#### Formative research

Four months before the initiation of the study, formative research was carried out in the form of focus group discussions. In total, six focus group discussions (FGDs) were conducted separately with each group of mothers and ANMs to explore the culturally appropriate foods for infants, the gap between knowledge and action of mothers, frequency of infant feeding, feeding practices, etc. using FGD guide (S5 File), till data saturation was achieved. Two FGDs were conducted with the ANMs and four FGDs were conducted with the mothers/caregivers. Prior consent was obtained from the participants before conducting the FGD. The mother groups had 6–8 participants who actively participated and discussed regarding culturally appropriate foods consumed by their infants, frequency of feeding, age at introduction of complementary foods and feeding practices of mothers. The facilitator also explored the different foods introduced at 6 months and 9 months and support that the mother receives from family for feeding the infant. The ANM groups discussed the reasons for undernutrition in community, current practices, barriers, facilitators of complementary feeding in the community and gap between knowledge and action of mothers. Result of the FGDs helped in the modifying the nutrition educational intervention as per the need of mothers and health workers, and development of child undernutrition tracking module for monitoring and supervision of child growth by health workers. Key messages regarding infant feeding and complementary foods were developed and modified based on formative research.

Pilot testing of the intervention and pretesting of the semi-structured questionnaires on mother-infant dyads, ANMs and AWWs was conducted in an area similar to the study area to modify the questionnaires, assess the feasibility of delivering the intervention, and fine-tuning the interventional module. About 40 mother-infant dyads were included in pilot testing where they were delivered the key messages and asked regarding the difficulties in understanding and following them. Similarly, ANMs were trained regarding nutrition education and counseling and strategy of delivering the intervention during routine home visits were devised. Pretesting was done in April 2017.

### 2. Baseline assessment phase

Baseline assessment of maternal knowledge of infant feeding, past feeding history, current infant feeding practices (frequency of feeding, number of meals and snacks per day), hand hygiene and responsive feeding practices was done by using a pretested semi-structured questionnaire (S6 File). The food frequency questionnaire was used to assess infant feeding practices over the past seven days before assessment [24]. Feeding on the day before the interview was used to note the current infant feeding practices.

#### Measurement of weight and length

The status of under-nutrition was assessed by anthropometry of the infants by a trained medical doctor. Infants were weighed using a digital weighing scale (Digital Salter Model 9000) with a maximum capacity of 150 kg and an accuracy of 0.1 kg [25]. It was calibrated weekly using standard weights and methods [26]. Recumbent length was measured using SECA height/ length measuring board (UNICEF) with a fixed head and sliding foot piece, with an accuracy of ±0.2 cm [27, 28]. The scales were placed on a flat surface and anthropometry was conducted using standard approved procedures [26]. The infants wore cotton nappy/ light diaper and weighed while being held in the arms of the mother. If the infant was crying, the measurement was deferred until he/she was pacified.

The baseline assessment of knowledge of ANMs regarding complementary feeding and responsive feeding was checked using a pre-tested semi-structured questionnaire (S7 File). They were asked regarding the status of growth monitoring and field-based activities related to infant feeding, which were routinely performed in their areas.

Baseline assessment was done in both intervention and control arm in June 2017.

### 3. Intervention phase

Two interventions were delivered in this phase. One is nutrition educational intervention and the other is web-based tracking of undernourished children.

#### a. Nutrition educational intervention

This intervention was delivered in classroom settings as well as community-based settings at health workers and the mother-infant dyad level. It had two modules i.e., health workers (S8 File) and mothers’ module (S9 File). These modules are described below:

- Module for Health workers
  Health workers were ANMs. This module had three parts and was delivered as:
  - Part 1. Orientation and introduction of health workers regarding complementary feeding
  - Part 2. Improving communication skills of health workers
  - Part 3. Tasks to be performed at home visit
- The ANMs were trained in nutrition counseling. They received training at baseline and the session was repeated after two months. They were provided nutrition education using health talks and videos on complementary feeding, hand hygiene and responsive feeding as per the modules. After the training, the ANMs conducted the home visit to counsel and support mothers for infant feeding practices every month. ANMs conducted fortnightly home visits for undernourished infants. During the home visit, the mother was asked regarding the breastfeeding, food items consumed during the last 24 hours, hand hygiene practices and responsive feeding. ANMs took interest in infant feeding, focused on praising what the mother was doing right and gave practical suggestions to them. The ANMs also recorded the weight and height of infants and delivered key messages to the mothers. Key messages on breastfeeding, age-appropriate complementary feeding, hand washing, and responsive feeding were delivered by them. These also included ways to enrich foods by adding *ghee*[refined oil]/oil/butter to the food and feeding advice during and after an episode of illness in the infant. The ANMs asked mothers to increase the quantity of food slowly over a while after the illness. Supervisory visits were conducted by the researcher (author) every month to assess their work. A routine meeting with ANMs was conducted and feedback provided to them helped to improve the implementation. The work plan provided to ANMs every week helped to keep tracking of nutritional status of infants. It provided individual attention to the MID and improved their feeding habits.
- *Module for mothers*
  Two sessions of nutrition education and counseling were conducted for the mothers/caregivers in classroom settings. The content of this intervention was similar to feeding recommendations given by the World Health Organization, 2001 [29]. Culturally appropriate food recipes were standardized by the dietician (author). The first session was conducted when the infants were 6–8 months old. The second session was conducted when the infants were 9–11 months old. Both the sessions were focused on age-appropriate complementary feeding and continuing breastfeeding of the infants with complementary feeding and appropriate complementary foods and snacks for infants. They were shown videos regarding the importance of hand hygiene and sanitation, maintaining hygiene while cooking, feeding and correct technique of handwashing. Each session lasted for 30–45 minutes and was conducted for 10–20 participants in each session. Talks, group discussions, and demonstrations were used to impart knowledge and skills related to infant and child feeding to mothers. Videos on complementary feeding, handwashing, and cooking nutritious foods were also shown to mothers/caregivers to impart knowledge creatively. The delivery of intervention through the medium of videos made it interesting for the mothers. The mothers paid more attention to the videos which were made in the local language, easy to understand and focused only on the key messages. The videos focused on the importance of clean hands and sanitation for the health of infants.

The control group continued to receive standard care delivered through routine health services offered by ANMs and AWWs. They received routine health education delivered through AWWs and feeding advice by ANMs at the time of routine immunization and home visits.

#### b. Digitized tracking of undernourished children

A digitized module was developed for tracking of the undernourished child. The infants were weighed every month in the *Anganwadi* centers by the ANMs. The anthropometric data of the infants were entered in an excel sheet in the computer and z score was calculated using the software WHO anthro 3.2.2 [30]. A monthly work plan showing the nutritional status was prepared for each ANM in the intervention area. This work plan was provided to all the ANMs in the area monthly, along with the routine mother and child tracking system work plan, so that they can also focus on mother-infant dyads with low infant growth along with their routine duties. The medical officer-in-charge of the ANMs were also involved in the supervision. A joint meeting was held with the researchers (authors) and medical officer and ANMs weekly to monitor the status of undernourished children as per the work plan.

The duration of the intervention phase was six months and mother infants dyads were followed up till 31^st^ December 2017.

### 4. Post-intervention phase

End line assessment of maternal knowledge of infant feeding, past feeding history, current infant feeding practices (frequency of feeding, number of meals and snacks per day), hand hygiene and responsive feeding practices was done by using the same pretested semi-structured questionnaire was used in the baseline (S6 File). The food frequency questionnaire was again used to assess infant feeding practices over the past seven days at the end-line [24].

The status of undernutrition was assessed by anthropometry of the infant and by calculation weight for age, weight for length and length for age Z scores. Similarly, the knowledge of ANMs on infant feeding and the status of growth monitoring was checked by the same pretested semi-structured questionnaire in both intervention and control arm as used in the baseline.

### Quality assurance

About 10% of the counseling sessions by the health workers with the mother-infant dyads in the field were directly observed and supervised by the authors in the study area, and accordingly, feedback to the health workers and mothers were provided to improve complementary feeding practices.

### Data collection

Data on anthropometry was collected at baseline and six months post-intervention in both intervention and control groups with the help of questionnaires and anthropometry tools. Data collection at three months post-intervention was done in the intervention area to gain knowledge regarding complementary feeding practices of infants.

### Data analysis

The primary outcome variable was mean change in the weight, and secondary outcomes were mean change in the length for age, mean change in the weight for length, change in level of maternal knowledge and practice of recommended infant and young child feeding practices following intervention, change in proportion of infants who were consuming foods from four or more food groups, and, change in proportion of infants who were consuming the minimum recommended number of meals per day. Statistical analysis was done by using Statistical Package for Social Sciences (SPSS) version 16. The anthropometric data of the infants was used to assess the nutritional status in WHO anthro 3.2.2 software and recorded in an excel sheet. Baseline characteristics between intervention and control groups were compared using the t-test for continuous variables and chi-square tests for categorical variables. Effectiveness was measured by calculating the difference in difference in mean change in weight between the intervention and control group. The difference in difference analysis was based on the assumption that there is no underlying time-dependent trend in the outcome related to intervention [31]. The control group is experiencing the same trend but not exposed to the intervention. Any event occurring during or the time of the intervention will equally affect the intervention and control group. A generalized estimate equation was used to analyze the longitudinal differences in categorical variables (pre and post-intervention) using STATA software version 13.

## Results

The themes generated from FGDs along with the supporting narratives are summarized below:

### Breastfeeding practices

The majority of mothers agreed that breast milk should be given until six months of age but they could not agree on the age till breastfeeding should be continued. Most of the mothers believed that it should be continued until the child continues to drink. Few women felt it should be continued until two years of age. Several mothers believed that they had inadequate milk production. They preferred to give animal milk in addition to breast milk to satisfy the hunger of the child. Fresh animal milk was perceived to be better than packaged milk.

“*I have buffalo in Punjab and I use buffalo milk only*. *My child is four months old and I am giving both breast milk and buffalo milk to him*. *But I breastfeed more and give buffalo milk sometimes*. *I felt that my breast milk was not adequate*. *So*, *my mother in law advised me to give a small amount of buffalo milk*.”

(Mother of a four months old child)

### Complementary foods and feeding practices

All respondents knew complementary foods like porridge, *khichadi* (rice cooked with lentils), *suji halwa*, *kheer* (rice cooked in milk), banana, boiled potato, eggs, etc. Home-cooked foods were perceived to be fresh and pure in quality than formula foods. Formula feeds were easy to feed but costly which the mothers could not afford. Most of the participants did not know about the consistency of foods or the frequency of foods. Many mothers introduced foods like pulse water or rice water first as these were thought to be easily digested.

*“I give a small amount of food like two to three spoonfuls only*. *I give it two to three times a day so that the child eats a Katori (small cup) in a day*. *This amount should be given to satisfy the hunger of the child*. *My mother-in-law refuses to feed the child frequently*. *She says that the child will pass stools frequently*.*”*

(Mother of a nine months old child)

### Cultural factors

Most of the mothers said that the initiation of complementary foods is celebrated in the family. Some families perform a *puja*(the act of worship) and give *kheer* (rice cooked in milk) to the child. The majority of them believed in the concept of hot or cold and heavy or light foodstuff. They thought that younger children should be given light foods like lentils or rice water. Heavy foods like meat and *parantha*(flatbread) should be avoided in younger children but can be given in older children.

*“Banana should not be given in winter as the baby will catch a cold*. *Sugarcane juice should not be given as the winter season is here*. *Egg*, *arhar dal (pigeon pea lentils)*, *brinjal should be avoided in summer as these are garam (hot) in nature*. *Maggi noodles should not be given to the baby as it is more bhari (heavy) and he can not digest these*.*”*

(Mother of a ten months old child)

### Effect of migration

Out of twenty-four respondents, the majority had migrated from neighboring states of Uttar Pradesh (10), Punjab (4), Uttrakhand (4), Delhi (1) and Bihar (2). Several respondents lived in remote villages. Most of them responded that common food items were not available in their native villages. Some mothers said that local markets had poor quality of food items. Mothers had to take some food items with them to the village for feeding the child. Most of the mothers felt that it is easier to cook food on the gas stove than on an earthen stove in the village. In some families, the elder family members objected to cooking food multiple times a day for a child. All mothers felt that every food item was available at Chandigarh easily. There was not much difference in food items fed to the children in the village.

“*When I go back to my native village I take foodstuffs from Chandigarh to cook there*. *I do not get the foodstuffs like suji (semolina)and good quality biscuits at the village*. *The quality of dal (lentils) is also different in the village*. *We sow dal(lentils) at the village which has good quality*. *Food is cooked on chullah (earthen stove) in the village while here it is cooked on gas*. *Also*, *food is cooked in the morning only*. *Kids do not like the food items in the village*. *My two kids are born here*. *So*, *they have developed the taste of this place*.*”*

(Mother of a seven months old child)

### Role of family

Several mothers reported that their husbands do not help in feeding the child due to lack of time, being busy in job and tiredness. Some men feel ashamed of doing household chores and do not help even when women are sick. Many women were living in a nuclear family and took care of the child alone. Few women reported that they are supported by husbands and other family members like the mother-in-law or sister-in-law.

### Background characteristics

There were 202 mother-infant dyads enrolled in the intervention and control group, respectively. The baseline characteristics of mother-infant dyads in both groups were similar with minimal differences as shown in Table 1. At end-line assessment, 190 and 191 mother-infant dyads could be followed up in the intervention group and control group, respectively. About 12 (6%) and 11 (5.5%) infants were lost to follow up due to migration in the intervention group and control group respectively. There was no mortality in any study group.

**Table 1:** Baseline characteristics of intervention and control group.

Variable	Intervention n = 202(%)	Control n = 202(%)	p-value
**Sex of infants**			
Male	96 (47.5%)	110 (55.4%)	0.16
Female	106 (52.8%)	92 (45.5%)	
**Birth weight (Mean± S.D.)**	2.80± 0.44	2.81± 0.44	0.82
**Age at enrollment in months (Mean± S.D.)**	5.41± 0.76	5.49± 0.67	0.28
**Weight (Mean± S.D.)**	6.56 ± 0.64	6.64± 0.52	0.51
**Length (Mean ±S.D.)**	64.32 ±1.95	65.07±1.67	<0.01
**Institutional delivery**	195 (95.5%)	200 (99%)	0.10
**Term Births**	(99.5%)	200(99%)	>0.99
**Parity**			
1	87 (43.1%)	103 (51.0%)	0.25
2	73 (36.1%)	61 (30.2%)	
3	28 (13.9%)	30 (14.9%)	
> = 4	14 (6.9%)	8 (4.0%)	
**Breastfeeding initiation after birth**			
Within 1 hour of birth	58 (28.7%)	69 (34.2%)	0.29
>1 hour—4 hours	100 (49.5%)	96 (47.5%)	
4–24 hours	15 (7.4%)	6 (3.0%)	
After 24 hours	25 (12.4%)	26 (12.9%)	
Not breastfed	4 (2.0%)	5 (2.5%)	
**Bottle feeding before six months of age**	55 (27.2%)	56 (27.7%)	0.91
**Exclusive breastfeeding**			
Yes	109 (54%)	109 (54%)	1.0
No	93 (46%)	93 (46%)	
**Maternal age in years** (Mean±S.D.)	25.2 ±4.1	25.2 ± 3.7	0.66
**Occupation of mother**			
Government/Private job	5 (2.5%)	6 (3.0%)	0.95
Homemaker	191 (94.6%)	190 (94.1%)	
**Type of family**			
Nuclear family	149 (73.8%)	126 (62.4%)	0.01
Joint family	53 (26.2%)	76 (37.6%)	
**Religion**			
Hindu	171 (84.7%)	168 (83.2%)	0.66
Muslim	24 (11.9%)	29 (14.4%)	
Sikh	7 (3.5%)	5 (2.5%)	
**Pucca house**	197 (97.5%)	199 (98.5%)	>0.99

### Assessment of growth parameters of infants

The mean weight and mean length of infants was calculated in both groups at baseline and end-line and effectiveness of the intervention was measured by calculating the difference in difference in mean change in weight between the intervention and control group. In the intervention group, the mean weight of infants at the end line was 8.8±0.8 kg versus 8.6±0.8 kg as compared to the control group (p = 0.04) [Table 2]. The mean length was 75.8±2.3 cm in the intervention group and 75.7±2.2 cm in the control group (p = 0.5). The difference in difference in mean change in weight between the intervention and control group was 0.27 kg which was found to be significant [p = 0.01]. The gain in length of the infants in the intervention group was significantly more than in the control group (0.9 cm, p<0.01). The weight for age Z score (WAZ) was also significantly higher in the intervention group (difference of means = 0.30, p< 0.01) and length for age Z score (LAZ) was also significantly higher (difference of means = 0.51, p<0.01).

**Table 2:** Comparison of mean weight and mean length of infants in the intervention and control group at baseline and end-line.

Variable	Baseline		After 6 months		Difference		Difference in difference of the means	p-value
	Intervention	Control	Intervention	Control	Intervention	Control		
Weight (Kg) (Mean± S.D.)	6.56 ± 0.64	6.64± 0.52	8.80 ± 0.8	8.64± 0.82	2.26	1.99	0.27	0.01
Length (cm) (Mean± S.D.)	64.32 ±1.95	65.07 ± 1.67	75.88 ± 2.27	75.73± 2.16	11.6	10.6	1.0	0.01
WAZ* (Mean± S.D.)	-1.01±0.73	-1.01± 0.64	-0.99± 0.69	-1.31± 0.78	0.016	-0.28	0.30	0.01
WLZ* (Mean± S.D.)	-0.82 ± 0.75	-0.98± 0.70	-0.92 ± 0.69	-1.15± 0.79	-0.095	-0.16	0.06	0.01
LAZ* (Mean± S.D.)	-0.57± 0.63	-0.43± 0.49	-0.71 ±0.67	-1.04± 0.66	-0.16	-0.67	0.51	0.01

*WAZ- weight for age Z-score, LAZ- length for age Z-score, WLZ- weight for length Z-score

One-tenth (11.6%) infant in the intervention group and 7.3% of infants in the control group were underweight at the beginning of the study (Table 3). At the end-line, the status of undernutrition was decreased (11.6% to 10%) in the intervention group, while in the control group the proportion of underweight infants had increased (7.3% to 18.8%, OR = 0.47, p = 0.01). At baseline, the infants in the intervention group were two times stunted compared to the control group (OR = 2.27) while at the end-line, more infants in the control group were stunted (OR = 0.53, p = 0.21). At baseline, more infants in the intervention group were wasted compared to the control group (11.6% vs. 8.4%, OR = 1.43), while at the end line more infants in the control group were wasted (OR = 0.42, p = 0.01).

**Table 3:** The proportion of underweight, stunting and wasting in the infants at baseline and end line in the intervention and control group.

Variable	Intervention N = 190 (%)	Control N = 191 (%)	Odds Ratio	p-value	Difference in difference	p-value
**Underweight**						
Baseline	22 (11.6)	14 (7.3)	1.65	0.16	-13.1	<0.01
Endline	19 (10)	36 (18.8)	0.47	0.01		
**Stunting**						
Baseline	3 (1.6)	1 (0.5)	2.27	0.39	-4.2	<0.01
Endline	6 (3.2)	11 (5.8)	0.53	0.21		
**Wasting**						
Baseline	22 (11.6)	16 (8.4)	1.43	0.3	-11.6	<0.01
Endline	14 (7.3)	30 (15.7)	0.42	0.01		

**Fig 3:**
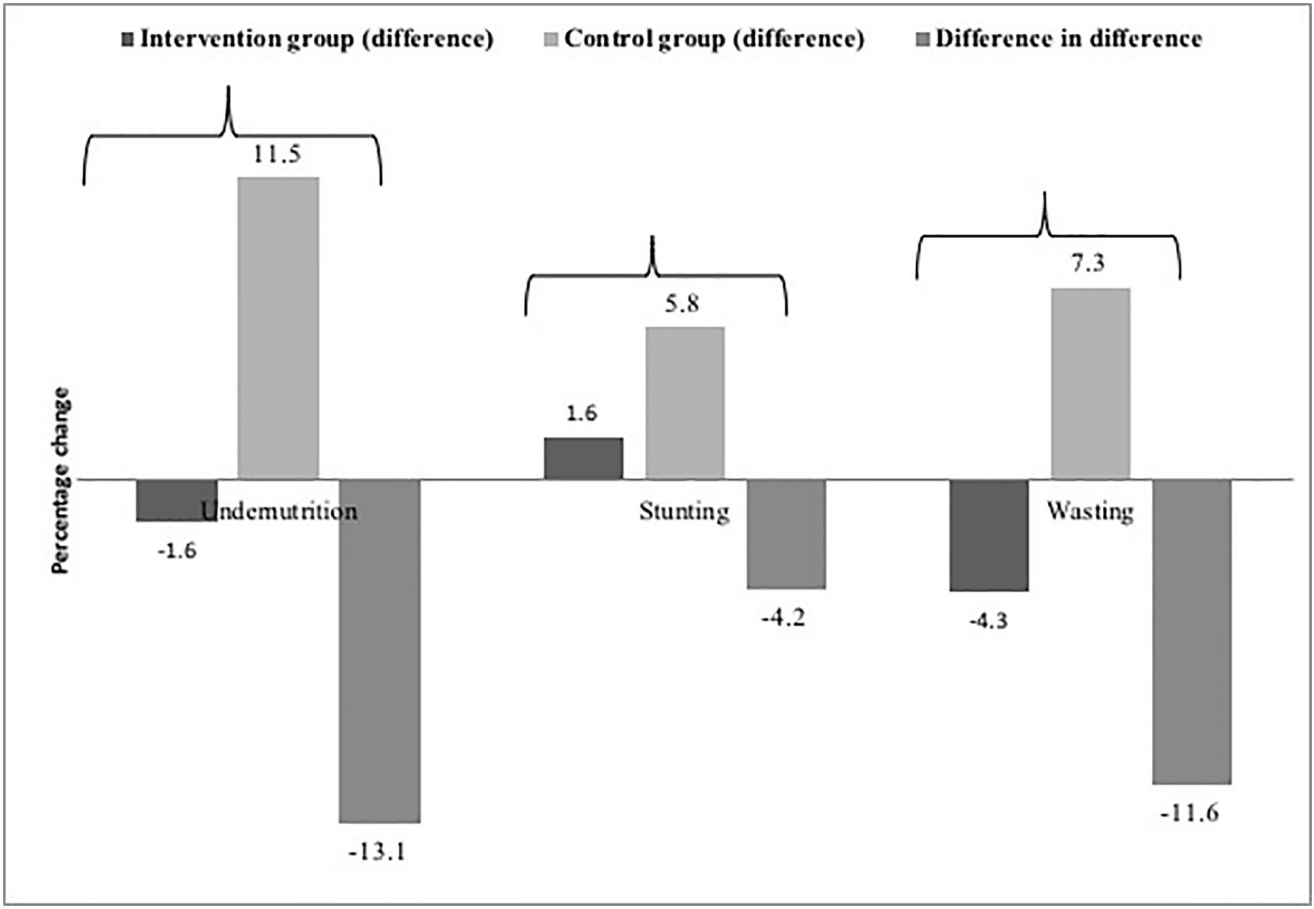
The difference in the proportion of underweight, stunting and wasting in the infants at baseline and end line in the intervention and control group.

The difference in difference analysis of the proportion of underweight, stunted and wasted infants at baseline and end line in the intervention and control group have shown that there was a net reduction of 13% in underweight children, 4.2% in stunting and 11.6% in wasting due to the intervention (Fig 3).

### Assessment of knowledge of mothers regarding infant feeding practices

The knowledge of mothers regarding breastfeeding and complementary feeding in both groups was similar in most areas at baseline (Table 4). Post-intervention, there was a significant improvement in certain knowledge parameters of the mothers in the intervention group as compared to the control group. Significantly more mothers knew about the appropriate age of introduction of complementary feeding (86.8% versus 62.8%, p<0.01), could name three healthy complementary foods (97.4% versus 77.5%, p<0.01) and snacks (74.2% versus 30.9%, p<0.01) for infant in intervention group as compared to the control group.

**Table 4:** Comparison of baseline and end line maternal knowledge regarding the feeding of infants between intervention and control group.

	Baseline			End-line			DID	p value
	Intervention n = 202 (%)	Control n = 202 (%)	p-value	Intervention n = 190(%)	Control n = 191(%)	p-value		
**Best milk for baby**								
Breast milk	192 (95.0%)	187 (92.6%)	0.21	190 (100%)	179 (93.7%)	<0.01	0.76	0.53
**Age till exclusive breastfeeding should be given**								
Up to 6 months	121 (59.9%)	142 (70.3%)	0.16	177 (93.2%)	137 (71.7%)	<0.01	-1.79	<0.01
**Bottle feeding to child**	136 (67.3%)	165 (81.7%)	<0.001	29 (15.3%)	110 (57.6%)	<0.01	3.16	<0.01
**Age of introduction of complementary feeding**								
At 6 months	97 (48.0%)	128 (63.4%)	0.002	165 (86.8%)	120 (62.8%)	<0.01	-2.14	<0.01
**Knowledge regarding frequency of feeding**	68 (33.7%)	102 (50.5%)	0.003	170 (89.5%)	109 (57.1%)	<0.01	-3.63	<0.01
**Thick consistency of foods**	4 (2%)	4 (2%)	0.92	137 (72.1%)	20 (10.5%)	<0.01	-8.63	<0.01
**Knowledge regarding complementary foods for infant**	115 (56.9%)	155 (76.7%)	<0.001	185 (97.4%)	148 (77.5%)	<0.01		
**Knowledge regarding healthy snacks for the infant**	28 (13.9%)	22 (10.9%)	0.36	141 (74.2%)	59 (30.9%)	<0.01	-4.74	<0.01
**Ghee/oil addition to complementary foods**	49 (24.3%)	48 (23.8%)	0.60	119 (62.6%)	46 (24.1%)	<0.01	0.39	<0.01
**Age till breastfeeding should be continued**								
Upto& beyond 2 years	81 (40.1%)	69 (34.2%)	0.13	175 (92.1%)	60 (31.4%)	<0.01	0.54	<0.01
**Commercial foods better than homemade foods**	44 (21.8%)	66 (32.7%)	0.001	38 (20.0%)	62 (32.5%)	<0.01	0.05	0.04
**Handwashing**	201 (99.5%)	199 (98.5%)	0.62	189 (99.5%)	188 (98.4%)	0.62	0	1.0
**Child threatened/bribed if not taking foods**	32 (15.8%)	18 (8.9%)	0.98	23 (12.1%)	18 (9.4%)	0.69	-1.61	<0.01

*DID: Difference in difference analysis

### Assessment of feeding practices of infants

Most of the infants were receiving complementary feeding in both groups at the end-line evaluation. However, more positive changes were observed in infant feeding practices in the intervention group [Table 5]. Significantly less number of infants were receiving bottle feeding in the intervention group as compared to the infants in the control group (35.8% versus 47.6%, p-0.04). A significant majority of the infants were receiving complementary feeding (92.6% versus 79.1%, p-0.01) and thick consistency foods(82.1% versus 41.9%, p<0.01) in the intervention group as compared to the infants in the control group. Significantly more infants in the intervention group were consuming food from four or more food groups at end-line assessment as compared to the control groups (83.7% vs. 61.8%) [p<0.01].

**Table 5:** End-line comparison of feeding practices of infants in the intervention and control group.

	Intervention n = 190 (%)	Control n = 191 (%)	p-value
**Current feeding practice**			
Complementary feeding	176 (92.6%)	153 (79.1%)	<0.01
**Age of introduction of complementary feeding**			
At 6 months	138 (72.6%)	87(45.5%)	<0.01
**Currently, baby receiving bottle-feeding**			
No	122 (64.2%)	100 (51.9%)	0.04
**Eating thick food first at the main meal**			
Yes	150 (78.9%)	53 (27.7%)	<0.01
**Consistency of food received**			
Thick	156 (82.1%)	79 (41.6%)	<0.01
**Mode of feeding child**			
With spoon	90 (47.4%)	76 (40.4%)	0.12
By hand	21 (11.1%)	31 (15.4%)	
Both spoon/ hand	77 (40.5%)	76 (40.4%)	
Self fed by hand/ spoon	2 (1.1%)	8 (3.7%)	
**Feeding tea/ sugar drinks**			
Yes	40 (21%)	68 (35.6%)	<0.01
**Eating food from four or more food groups**	159 (83.7%)	118 (61.8%)	<0.01

*None of the children was on only breastfeeding in both the areas.

### The extent of implementation of the intervention

Overall, the implementation of certain aspects of the intervention was very good like ANMs delivering key messages to mothers, providing appropriate feeding advice to mothers and supervision of ANMs by the researcher. It was found that all the tasks by the ANMs were fully implemented in the areas, except for the recording of the weight of infants every month and monthly follow up of infants for some ANMs. The monthly follow up of infants and supervision of the ANMs by the researcher was observed to be fully implemented and as per the plan.

## Discussion

This study highlighted that nutrition educational intervention delivered through the involvement of ANMs in the health system can significantly improve the knowledge of mothers regarding complementary feeding and infant feeding practices in the intervention group especially regarding giving thick consistency foods to the infants and breastfeeding to be continued up to and beyond two years of age. This also resulted in a significantly higher proportion of infants who were started on complementary feeding at 6 months of age (72.6% versus 45.5%, p<0.01), and significantly improved weight gain (net gain of 0.27 kg, p< = 0.01) and linear growth in infants (net gain of 0.9 cm, p<0.01). Improvement was observed for responsive feeding of infants by mothers like threatening or bribing infants if they were not taking food in the intervention group (12.1% versus 9.4%, p<0.01).

This is a community-based study with a quasi-experimental study design. Nutrition education interventions do not readily lend themselves to randomized control trials. Participants may have a desire for a specific intervention and advice, making blinding and consent difficult. If both intervention and control groups were randomly selected within an area, there was a high chance of contamination and we would not have been able to find out whether results were due to intervention or any other cause. A similar method of selecting the study groups where complete randomization is not possible has been used by Gulden et al. study (2000) [15]. The quasi-experimental design has many advantages where a true experiment is not feasible. Even a non-randomized control group help to reduce threats to both the internal and external validity of the study [32]. It facilitates in providing a large pool of eligible population for recruitment, involves more settings and enhances generalizability. This quasi-experimental study included formative research in the pre-intervention phase which helped in designing tailor-made intervention as per the needs of the community. Formative research helped to identify the needs and practices of the community and also explored the potential to change behavior. Other studies have also reported conducting formative research to develop interventions including feeding recommendations, flipbooks containing photographs on feeding practices and behaviors, or culturally appropriate messages and which were delivered through the local health services in randomized clusters of villages in rural Indian settings [14,33]. A quasi-experimental study conducted recently by Singh V et al. in Uttar Pradesh, India used the feeding recommendation of the Integrated Nutrition and Health Program of CARE-India. However, no formative research was done before the delivery of that intervention [34]. In a randomized controlled trial done in Peru, formative research before the intervention was conducted and government facilities were involved in the intervention and supportive supervision was done to correct any deficiencies in nutrition education [13]. An intervention study conducted in Pakistan used local women workers trained in early childhood development assessment, child care and nurture who conducted nutritional counseling and dietary assessment [35]. However, there was no control group in this study. None of the studies utilized the medium of videos to impart nutrition education which was done in the present study. Nutrition education videos are effective and interactive in delivering intervention [36]. The work plan provided to ANMs every week helped to keep track of the nutritional status of infants in this study. It provided individual attention to the mother-infant dyads and improved their feeding habits.

The height/length measuring board used in this study to measure length is recommended by UNICEF to be used in anthropometry assessment of children [26] and digital scales are recommended by WHO [26]. Penny et al. [13], Bhandari et al. [14], Zahid Khan et al. [34], Sahaet al. [37] and National Family Health Survey round 4 (2015–16) [38] have used the digital weight measuring scales and height/length measuring boards in their studies and surveys respectively for anthropometry assessment, which is similar to this study.

We have used the difference in difference analysis in this study to measure the effectiveness of the nutrition educational intervention. Wing et al have mentioned that DID is often used to study the causal relationships in the public health settings where conducting randomized control trials may not be feasible or unethical. Although DID may not be the perfect substitute for randomized control trials, it is a feasible way to understand causal relationships between the intervention and outcome [39]. This type of analysis was also considered in measuring the net effect of the nutrition-based intervention to estimate the change in dietary intake from baseline to end-line in the intervention and control arm in Kaur et al. study [40].

In a study conducted by Bhandari N et al. in India, the mean birth weight of infants measured at baseline assessment was found to be similar to the present study (2.7±0.4 kg versus 2.8±0.4 kg) [14]. The mean weight at 6 months was 6.4 kg while in the present study the mean weight at the age of 5.5 months was 6.6 kg. However, there were other differences like the majority of infants were born at home (72% versus 3.5%). In a similar study conducted by Singh V et al. in Uttar Pradesh, the mean birth weight of newborns was 3.04±0.3 kg [34]. However, about 33.1% of infants were born at birth order of four or above in the aforementioned study while in the present study only 6.9% of infants were fourth born child and more than 79% of the infants were either first or second born respectively. It might be a reason for improved nutritional status in the present study as parents could pay more attention to the only child. Also, the utilization of health facilities was good as observed by the higher number of institutional deliveries (>96%), and immunized children (99%) in the present study. In a hospital-based study in Karnataka, nutrition education was delivered every month to the caregivers of infants that resulted in about 77.5% of the mothers starting complementary feeding at six months of age, which is similar to the findings of this study (72.6%) [41]. However, 22% of the mothers were giving bottle feeding in the study mentioned above as compared to 35.8% of mothers in our study.

The standard deviations reported in this study are much lower than the suggested standard deviations reported by Mei and Grummer-Strawn estimations in a cross-country analysis [42]. This could be because of the difference in the sample size of theirs’s and this study. The sample size of this study constituted a small homogenous population living in peri-urban areas of the same city, while the sample size of Mei and Grummer-Strawn study constituted nationally representative demographic health survey data from 51 different countries. Secondly, the background characteristics of both study groups (intervention and control groups) were almost similar in terms of vulnerability, environmental conditions and health service utilization e.g. institutional delivery rate, immunization status, etc. in this study that might also have contributed to the low SDs.

Many other studies have tried to improve malnutrition among infants through nutrition education. Penny ME et al study in Peru showed similar results, where intervention group children gained 295-gram weight (p = 0.014) and 1.07 cm length (p <0.0003) more than the control group children at the end of 18 months. They were also eating more energy-dense and thick consistency meals (31% versus 20%, p = 0.03) [13]. In another community-based study conducted by Roy SK et al. in Bangladesh, the intervention group infants showed more weight gain (0.86 versus 0.77 kg, p = 0.053) as compared to infants in the control group. This intervention was culturally appropriate and involved fathers and old male members as they were the main decision-makers in the family [16]. In a cluster-randomized trial conducted by Saleem AF et al (2014), in Karachi, education messages were delivered to mothers for 30 weeks, and weight gain of 350 gram (p = 0.001) and 0.66 cm increase in length (p = 0.001) was observed in infants in the intervention group [43]. It showed that nutrition education intervention is successful in those populations where food security is present. The study conducted in rural Haryana also showed similar results, as a significant gain in length (0.32 cm) was observed in infants in the intervention group after 18 months. Male infants showed more improvement in length (0.51) as compared to female infants in that study [14]. In a study conducted in Pakistan, nutrition counseling targeting mothers were delivered at two sites (Tando Jam and Quetta) which had the largest impact on the children who were mildly wasted at baseline. There was a decline in the prevalence of wasting (Tando Jam- 81% to 60%, Quetta- 82% to 49%) and also an increase in the number of meals taken by children per day [34]. The present study had been conducted in an urbanized village where more population lived in nuclear families and mothers were more likely to respond to ANM's advice. A latest quasi-experimental study done in India showed that after delivery of an education package to the pregnant mothers, their infants showed a gain in mean weight for age z score (-2.1 versus -2.4, p = 0.003). At 12 months, there was a reduction in the prevalence of underweight infants (58.5% versus 69.3%, p = 0.047) [33]. Also in this study, the odds of intervention infants having low weight for age were significantly low at the age of twelve months (Adjusted OR-0.5, CI = 0.3–0.9) [33]. A study conducted by Nikiema L et al (2017) in Burkina Faso showed that effect of nutrition counseling intervention on stunting was not significant after 18 months (OR-1.0, p = 0.89) whereas, in our study, significantly fewer infants in intervention group were stunted when compared with control infants (OR = 0.53, p = 0.21) [44]. In a similar study done in Pakistan, wasting was reduced by 12% in intervention infants. But at end-line evaluation, no significant difference was noted among the study groups (adjusted OR = 0.10), whereas in our study significantly less intervention group infants were wasted as compared to control infants (OR = 0.42, p = 0.01) [43]. In the present study, the proportion of underweight infants had increased in the control group (7.3% to 18.8%; OR = 0.47, p = 0.01) while it was reduced in the intervention group (11.6% to 10%).

One of the strengths of the study was the inclusion of tracking of undernourished infants in the routine health system. This novel concept of tracking through the provision of a computer-generated weekly work plan to the ANMs helped to monitor the growth of infants with a focus on undernourished infants. A similar concept has been used by the Government of India under the recently launched *Poshan Abhiyaan* (National Nutrition Mission) in which *Anganwadi* worker records the weight and height every month through the software application to track under-nutrition, stunting and wasting [45]. In the study, personal visits to undernourished infants every fortnight helped to solve maternal problems in complementary feeding. More emphasis was given on feeding semi-solid/ solid foods at least 3–4 times a day and less on feeding milk. ANMs also advised continuing feeding in ill infants during illness. The investigator did regular supportive supervision of sessions. It led to the confidence-building of ANMs who could deliver the intervention without the fear of being judged. The videos on complementary feeding and hand washing helped the mothers to relate to the intervention practically. Recently, the Government of India has introduced Home Based Care of Young Child (HBYC) program where the number of home visits by ANM/ASHA has increased from seven visits within 42 days of life to additional visits at 3, 6, 9 12 and 14 months by the ANMs to monitor growth and development of young children [46]. This also indicates that ANMs/ASHAs are entrusted with an additional role in preventing undernutrition among children in the health system. ANM visits at least 10% of the houses of young children that are visited by ASHA along with her as part of the supportive supervision, which is similar to the data validation for maintaining the quality of data in this study.

Overall, the implementation of the intervention was good. There were many challenges in the implementation of the intervention. The Government of India launched a Measles-Rubella Campaign midway during the intervention and ANMs were engaged in immunizing the children. However, the mother-infant dyads were given nutrition advice even during the time of immunization. Frequent movement of some families within the intervention area made them difficult to track but these families were traced via telephone and kept under the study. The study population was vulnerable and had limited food security; hence the similar effects might be replicated in such populations.

Some of the limitations of the study include loss to follow up of malnourished infants who migrated from the area. As the list of infants was obtained from the *Anganwadi* centers, any infant who was not registered in the *Anganwadi* centers was not included in the study leading to selection bias. The proportion of children with stunting was found to be higher in the intervention group as compared to the control group (8.4% vs 1.5%) at baseline may have led to an unmeasured effect during the intervention period due to selection bias. However, we have tried to reduce the unmeasured effect during the period of intervention by doing a difference in difference analysis. Those infants whose mothers were unavailable due to work and did not give consent were not included in the study. Another limitation was the noninvolvement of AWWs in the study. The intervention would be better supported and sustainable if AWWS are involved in the implementation. The study was single-blinded so the possibility of bias cannot be ruled out. The interviewer's knowledge of the group could have influenced the interpretation of responses during the data collection period. The better growth pattern was seen in those infants who had healthy growth parameters in early life. However, the overall feeding habits were better in the intervention group as compared to the control group.

This study provided evidence that growth and complementary feeding practices of infants can be improved through nutrition education regarding culturally appropriate foods in vulnerable populations with limited food security through the routine health care delivery system by developing a computerized tracking mechanism. The services provided through the government health system have great potential in terms of sustainability, but only a few studies have utilized them. Even if the workers are replaced, the improved health system will help to continue to serve these infants. The inclusion of routine tracking of infants via the health system is a novel concept that needs further exploration. This study supports the formulation of a robust policy for utilizing the health system for routine tracking in a package of health interventions to combat malnutrition and improve the growth and feeding of infants in their formative years. However, the impact of the intervention on micronutrient intake and dietary diversity needs to be assessed. Further research is required to assess the long-term impact of this intervention on the migrant population.

## Supporting information

S1 File Study protocol. (DOCX)

S2 File TREND statement checklist. (DOCX)

S3 File Informed consent form. (DOCX)

S4 File Participant information sheet. (DOCX)

S5 File Focus group discussion guide for mothers/ caregivers. (DOCX)

S6 File Questionnaire for mother-infant dyad. (DOCX)

S7 File Questionnaire for health workers. (DOCX)

S8 File Training modules for health workers. (DOCX)

S9 File Training module for mothers. (DOCX)

S10 File Data set. (XLSX)

S11 File Code book. (DOCX)

## References

[1] Levels and trends in child mortality: Report 2014. New York: United Nations Children’s Fund. 2014.

[2] Fact Sheet—Infant and young child feeding [Internet]. World Health Organization. 2017. http://apps.who.int/mediacentre/factsheets/fs342/en/index.html. Accessed on 21 April 2019.

[3] World Bank. 2012. India—Nutrition at a glance. Nutrition at a glance; India. Washington, DC; 2012.

[4] Government of India. National Family Health Survey, round 4. 2015-16. India Fact Sheet. 2015.

[5] Lokshin M, Das Gupta M, Gragnolati M, Ivaschenko O. Improving Child Nutrition ? The Integrated Child Development Services in India. Wiley-Blakewell. 2005;36(4):613–40.

[6] Mohan P, Kishore B, Singh S, Bahl R, Puri A, Kumar R. Assessment of Implementation of Integrated Management of Neonatal and Childhood Illness in India. J Heal Popul Nutr. 2011;29(6):629–38. 10.3329/jhpn.v29i6.9900 PMC3259726 22283037

[7] Srivastava A, Gope R, Nair N, Rath S, Sinha R S P. Are village health sanitation and nutrition committees fulfilling their roles for decentralized health planning and action? A mixed method study from rural eastern India. BMC Public Health. 2016;16(1):59. 26795942 10.1186/s12889-016-2699-4 PMC4722712

[8] Government of India. Guidelines for enhancing optimal infant and young child feeding practices. Ministry of health and family welfare. 2013.

[9] Chaturvedi A, Nakkeeran N, Doshi M, Patel R B S. Capacity of frontline ICDS functionaries to support caregivers on infant and young child feeding (IYCF) practices in Gujarat, India. Asia Pac J Clin Nutr. 2014;23(3): S29–37. 25384724 10.6133/apjcn.2014.23.s1.04

[10] United Nations. Sustainable Development Goals Report 2016. UN; 2016.

[11] Government of India. National Health Policy, 2017.

[12] Bhutta ZA, Ahmed T, Black RE, Cousens S, Dewey K, Giugliani E, et al What works? Interventions for maternal and child undernutrition and survival. Lancet. 2008;371(9610):417–40. 10.1016/S0140-6736(07)61693-6 18206226

[13] Penny ME, Creed-Kanashiro HM, Robert RC, Narro MR, Caulfield LE, Black RE. Effectiveness of an educational intervention delivered through the health services to improve nutrition in young children: a cluster-randomised controlled trial. Lancet. 2005;365(9474):1863–72. 10.1016/S0140-6736(05)66426-4 15924983

[14] Bhandari N, Mazumder S, Bahl R, Martines J, Black RE, Bhan MK, et al An educational intervention to promote appropriate complementary feeding practices and physical growth in infants and young children in rural Haryana, India. J Nutr. 2004;134(9):2342–8. 10.1093/jn/134.9.2342 15333726

[15] Guldan GS, Fan H-C, Ma X, Ni Z-Z, Xiang X, Tang M-Z. Culturally appropriate nutrition education improves infant feeding and growth in rural Sichuan, China. J Nutr. 2000;130(5):1204–11. 10.1093/jn/130.5.1204 10801920

[16] Roy SK, Jolly SP, Shafique S, Fuchs GJ, Mahmud Z, Chakraborty B, et al Prevention of malnutrition among young children in rural Bangladesh by a food-health-care educational intervention: a randomized, controlled trial. Food Nutr Bull. 2007;28(4):375–83. 10.1177/156482650702800401 18274163

[17] Census of India—Census Terms. Office of the Registrar General & Census Commissioner, India, New Delhi. n.d. http://censusindia.gov.in/Data_Products/Library/Indian_perceptive_link/Census_Terms_link/censusterms.html (accessed 1 August 2019).

[18] Urban health resource centre. Key indicators for urban poor in India from NFHS-3 and NFHS-2 2008:1–2. http://uhrc.in/downloads/Factsheet-India.pdf (accessed 4 July 2019).

[19] Chandramouli C. Census of India 2011: rural-urban distribution of the population (Provisional population totals). Indian Ministry of Home Affairs New Delhi Vol. 4 2011.

[20] USGS The National Map—Advanced Viewer. https://viewer.nationalmap.gov/advanced-viewer/. Accessed 9 July 2019.

[21] Anganwadi centers. https://data.gov.in/dataset-group-name/anganwadi-centers. Accessed 19 April 2019.

[22] National Urban Health Mission: Guidelines for ASHA and Mahila Arogya Samiti in the Urban Context Guidelines for ASHA and Mahila Arogya Samiti in the Urban Context. 2014.

[23] Kirkwood BR, Sterne JAC. Essential medical statistics. Second ed John Wiley & Sons; 2010 413–28 p.

[24] Mahajan R, Malik M, Bharathi AV, Lakshmi PVM, Patro BK, Rana SK, et al Reproducibility and validity of a quantitative food frequency questionnaire in an urban and rural area of northern India. Natl Med J India n.d.;26:266–72. 25017832

[25] Salter Model 9000 weighing scale. Specifications. https://www.price-hunt.com/health-care/salter-model-9000-weighing-scale.php. Accessed 3 August 2019.

[26] WHO. Measuring a Child’s Growth. Training courseon child growth assessment. WHO Child Growth Standards. https://www.who.int/childgrowth/training/module_b_measuring_growth.pdf. Accessed on 2 August 2019.

[27] UNICEF. Height/length measuring boards. UNICEF. Technical Bulletin No.18. 2012.

[28] UNICEF Target Product Profile Height/length Measurement Device(s). 2016. https://www.unicef.org/supply/files/HMD_TPP_V2.0.pdf

[29] World Health Organization. Complementary feeding: report of global consultation and summary of guiding principles for complementary feeding of the breastfed child. Geneva; 2001.

[30] Child growth standards: WHO anthro (version 3.2. 2, January 2011) and macros. World Health Organization.[Online] January. 2011.

[31] Dimick JB, Ryan AM. Methods for Evaluating Changes in Health Care Policy. JAMA 2014;312:2401 10.1001/jama.2014.16153 25490331

[32] Axelrod DA, Hayward R. Nonrandomized interventional study designs (quasi-experimental designs). Clin. Res. Methods Surg., Humana Press; 2007, p. 63–76. 10.1007/978-1-59745-230-4_4

[33] Vazir S, Engle P, Balakrishna N, Griffiths PL, Johnson SL, Creed-Kanashiro H, et al Cluster-randomized trial on complementary and responsive feeding education to caregivers found improved dietary intake, growth and development among rural Indian toddlers. Matern Child Nutr. 2013;9(1):99–117. 10.1111/j.1740-8709.2012.00413.x 22625182 PMC3434308

[34] Singh V, Ahmed S, Dreyfuss ML, Kiran U, Chaudhary DN, Srivastava VK, et al An integrated nutrition and health program package on IYCN improves breastfeeding but not complementary feeding and nutritional status in rural northern India: A quasi-experimental randomized longitudinal study. PLoS One. 2017;12(9):e0185030 10.1371/journal.pone.0185030 28931088 PMC5607187

[35] Zahid Khan A, Rafique G, Qureshi H, Halai Badruddin S. A nutrition education intervention to combat undernutrition: experience from a developing country. *ISRN Nutr*. 2013;2013:210287. Published 2013 Feb 5. 10.5402/2013/210287 24967253 PMC4045279

[36] Yadav S, Rawal G. The HIFA and the Health Phone: Laying the foundation for combating malnutrition in India. Int J Heal Sci Res. 2015;5(7):368–71.

[37] Saha KK, Frongillo EA, Alam DS, Arifeen SE, Persson LA, Rasmussen KM. Use of the new World Health Organization child growth standards to describe longitudinal growth of breastfed rural Bangladeshi infants and young children. *Food Nutr Bull*. 2009;30(2):137–144. 10.1177/156482650903000205 19689092 PMC4425403

[38] Indian Institute of Population Sciences. Clinical Anthropometric Biochemical (CAB) Manual. Nationa Family Health Survey 2015–16 (NFHS 4). Government of India. http://rchiips.org/NFHS/NFHS4/manual/NFHS-4%20Biomarker%20Field%20Manual.pdf Accessed 3 August 2019.

[39] Wing C, Simon K, Bello-Gomez RA. Designing Difference in difference studies: Best Practices for Public Health Research. Annu Rev Public Health. 2018 39:453–469. https://www.annualreviews.org/doi/pdf/10.1146/annurev-publhealth-040617-013507. Accessed 3 August 2019. 29328877 10.1146/annurev-publhealth-040617-013507

[40] Kaur J, Kaur M, Webster J, Kumar R. Protocol for a cluster randomised controlled trial on information technology-enabled nutrition intervention among urban adults in Chandigarh (India): SMART eating trial. *Glob Health Action*. 2018;11(1):1419738 10.1080/16549716.2017.1419738 29370744 PMC5795704

[41] Rao S, Swathi PM, Unnikrishnan B, Hegde A. Study of complementary feeding practices among mothers of children aged six months to two years-A study from coastal south India. Australas Med J. 2011;4(5):252 23393516 10.4066/AMJ.2011.607 PMC3562932

[42] Mei Z, Grummer-Strawn LM. Standard deviation of anthropometric Z-scores as a data quality assessment tool using the 2006 WHO growth standards: a cross country analysis. *Bull World Health Organ*. 2007;85(6):441–448. 10.2471/BLT.06.034421 17639241 PMC2636355

[43] Saleem AF, Mahmud S, Baig-Ansari N, Zaidi AKM. Impact of maternal education about complementary feeding on their infants' nutritional outcomes in low-and-middle-income households: a community-based randomized interventional study in Karachi, Pakistan. J Health Popul Nutr. 2014;32(4):623 25895196 PMC4438693

[44] Nikièma L, Huybregts L, Martin-Prevel Y, Donnen P, Lanou H, Grosemans J, et al Effectiveness of facility-based personalized maternal nutrition counseling in improving child growth and morbidity up to 18 months: A cluster-randomized controlled trial in rural Burkina Faso. PLoS One. 2017;12(5):e0177839 10.1371/journal.pone.0177839 28542391 PMC5444625

[45] Government of India. National Nutrition Mission: Administrative Guidelines. Ministry of Women and Child Development. New Delhi: 2018. https://icds-wcd.nic.in/nnm/NNM-Web-Contents/UPPER-MENU/AdministrativeApproval-Guidelines/Administrative_Guidelines_NNM-26022018.pdf.

[46] Operational guidelines—Home based care for young child (HBYC). A joint initiative of Ministry of Health and Family Welfare & Ministry of Women and Child Development. 2018. https://www.nhm.gov.in/images/pdf/programmes/RBSK/Operational_Guidelines/HBYC_Guidelines.pdf.

