# Supplementary material for: Effectiveness of a culturally appropriate nutrition educational intervention delivered through health services to improve growth and complementary feeding of infants: A quasi-experimental study from Chandigarh, India
Source: PLoS One. 2020 Mar 17;15(3):e0229755. doi: 10.1371/journal.pone.0229755 (PMC7077818; doi:10.1371/journal.pone.0229755)
Supplement: S1 File — (DOCX) [file pone.0229755.s001.docx]

**S1 File. Study protocol submitted to Institute Ethics Committee.**

**PLAN OF THESIS**

**EFFECTIVENESS OF A CULTURALLY APPROPRIATE NUTRITION EDUCATIONAL INTERVENTION DELIVERED THROUGH HEALTH SERVICES TO IMPROVE GROWTH AND COMPLEMENTARY FEEDING OF 6 MONTHS TO 1 YEAR OLD INFANTS IN CHANDIGARH: A QUASI EXPERIMENTAL STUDY.**

SUBMITTED IN PARTIAL FULFILLMENT OF THE REQUIREMENT FOR THE

DEGREE OF

**M.D. COMMUNITY MEDICINE**

OF THE

POSTGRADUATE INSTITUTE OF MEDICAL EDUCATION AND RESEARCH,

CHANDIGARH

By

Dr. Nikita Sharma

Junior Resident

Department of Community Medicine, School of Public Health,

Post Graduate Institute of Medical Education and Research,

Chandigarh

GUIDE

Dr. Madhu Gupta

Additional Professor

Department of Community Medicine, School of Public Health,

Post Graduate Institute of Medical Education and Research,

Chandigarh

CO-GUIDES

| Dr. Arun Kumar Aggarwal | Mrs. Mutyalamma Gorle, |
| --- | --- |
| Professor | Assistant Dietician |
| Department of Community Medicine, | Dietetics Department, |
| School of Public Health |  |
| Post Graduate Institute of Medical | Post Graduate Institute of Medical |
| Education and Research, Chandigarh | Education and Research, Chandigarh |

**INDEX**

| **Sr.No.** |  | **Contents** | **Page no.** |
| --- | --- | --- | --- |
|  |  |  |  |
| 1. | Introduction |  | 1 |
|  |  |  |  |
| 2. | Review of literature |  | 4 |
|  |  |  |  |
| 3. | Aims and Objectives |  | 18 |
|  |  |  |  |
| 4. | Methodology |  | 19 |
|  |  |  |  |
| 5. | Data collection |  | 25 |
|  |  |  |  |
| 6. | Statistical Analysis |  | 26 |
|  |  |  |  |
| 7. | Ethical considerations |  | 26 |
|  |  |  |  |
| 8. | Annexure |  | 27 |
|  |  |  |  |
| 9. | References |  | 56 |
|  |  |  |  |

**SUMMARY**

Under-nutrition among under five-year-old children is a public health problem in India. It makes children vulnerable for many diseases and untimely death. The various causes of under-nutrition include lack of availability of food, inadequate care and poor knowledge of feeding practices of mothers or care givers and lack of access to health care services. Despite having many supplementary feeding programs, the nutritional status of children has not seen much improvement in India. Malnutrition rate is high between the ages of 6 to 24 months because breast milk alone is not enough to meet the nutritional needs of the child and other foods are introduced to fulfill them. Complementary feeding is introduced in only 55.8% infants between 6-9 months of age, with only 44% of breastfed children being fed at least the minimum number of times recommended*.* Only half of them consume the food from three food groups. There is no mechanism to monitor growth of the children in the routine health system, as *anganwadi* workers working under integrated child development scheme (ICDS) under women and child development department have the primary responsibility of monitoring the growth of children between 0-6 years. But there is limited evidence that the ICDS program is meeting its goals of reducing child malnutrition in India. Hence, there is a need to strengthen the involvement of the auxiliary nurse midwives/ accredited social health activists in monitoring the growth of children belonging to her catchment area. Also there is a need to implement sustainable interventions through existing health care system that provide individualized care to improve the maternal/caregiver’s feeding practices so as to improve the nutritional status of children. However, effectiveness of such interventions needs to be evaluated before the large-scale implementation of such interventions in the routine program.

The present study is planned with an aim to ascertain the effectiveness of a culturally appropriate nutritional education intervention delivered through health services to improve growth and complementary feeding of infants of age 6 to 12 months in Chandigarh; and to develop a child undernutrition tracking module for health workers to monitor child growth. The study design will be quasi-experimental. The study and control area will be selected purposively. The study will be conducted among vulnerable, migrant population usually belonging to lower/lower middle socioeconomic groups. The primary end point will be mean change in weight for age. The study population will be mother infant dyad with the age of infants between 4-6 months. Mother infant dyad will be enrolled at the baseline, and they will be followed up for 6 months after initiation of the intervention. Prior formative research in the form of focus group discussions will be conducted with mothers, health workers and community workers to explore culturally appropriate foods, affordability and knowledge regarding feeding practices. At the baseline, a pretested semi-structured questionnaire will be used to assess the knowledge and feeding practices of mothers and health workers. A baseline anthropometry of the infants will be done. During the intervention phase, both mothers and health workers will be trained as per modules of World Health Organization. The mothers will be provided nutritional education about culturally appropriate, easily available, and affordable low cost home based standard recipes that they will feed their children. The health workers will support mothers at individual level and do the growth monitoring of the children. A monthly work plan will be prepared for each health worker for monitoring growth of the infants. The resident will do supervision of health workers in the field every two months. At the end of the study, the same pretested semi-structured questionnaire and anthropometry will be used to find the improvement in feeding practices and growth respectively. The anthropometric data will be entered in excel sheet and z sores will be calculated. Statistical analysis will be done by using Statistical Package for Social Sciences version 16. Effectiveness of the intervention will be measured by calculating the difference in difference in mean change in weight between the intervention and control group.

**INTRODUCTION**

Under nutrition is the underlying cause of 45% of global deaths for children under 5 years [1]. Around half of under-five deaths occur in only five countries: India, Nigeria, Democratic Republic of Congo, Pakistan and China [1]. Globally in 2013, 161.5 million children under 5 were estimated to be stunted and 50.8 million were estimated to have low weight-for-height. The prevalence of childhood malnutrition remains high in the developing world [2]. Early childhood nutritional deficiencies lead to inadequate growth, which in turn impairs brain development, creates academic difficulties and can lead to a lifetime of diminished earning capacity and an elevated risk of non-communicable diseases.[3] As per recent World Bank report, it is reported that malnutrition remains the single largest cause of child mortality; economic growth alone does not solve malnutrition; investing in nutrition is a cost effective intervention [4].

Protein energy malnutrition is identified as a major health and nutrition problem in India[5]. As per UNICEF's estimates (2009); 48% of Indian children less than 5 years of age were stunted, 43% were underweight, and 20% of children had wasting. In global ranking of stunting prevalence, India ranked 11 highest out of 136 countries [6]. Among urban poor children below five years of age, 54 % were stunted and 47% were wasted as compared to 50.7% and 45.6% of their rural counterparts. Over the past several years, India has failed to see any remarkable progress in infant feeding practices. Complementary feeding is introduced in only 55.8% infants between 6-9 months of age, with only 44% of breastfed children being fed at least the minimum number of times recommended*.* Only half of them also consume the food from three food groups [7]. The Government of India has initiated several large scale supplementary feeding programs in last 65 years, like integrated child development scheme (ICDS) through ministry of women and child development. However, there is limited evidence that the ICDS program is meeting its goals of reducing child malnutrition in India [8]. Integrated management of neonatal and childhood illness (IMNCI) was introduced as a strategy for an integrated approach to the management of childhood illness including malnutrition, but this program is yet to make an impact on improving malnutrition [9,10]. The nutrition education regarding complementary feeding is provided at community outreach level through auxiliary nurse midwives (ANM) along with the support from accredited social health activists (ASHA) and *anganwadi* workers at village health and nutrition days (VHND) through village health and sanitation committees (VHSC), routine immunisation sessions, IMNCI/ sick child consultation and through outpatient services like immunisation or sick child clinic. But in a study conducted in Orissa and Jharkhand, it was observed that although nutritional education was being provided at village health and nutrition days (VHND), health workers focused mainly on sanitation, record keeping and referral activities [11]. Guidelines for enhancing optimal infant and young child feeding practices were introduced in 2013 and *anganwadi* workers were trained, but a study conducted in Gujarat reported that *anganwadi* workers had more knowledge regarding feeding practices than the care givers/ mothers. They did not take feeding history, had poor listening skills and were unable to provide need-based advice to the caregivers [12].

Malnutrition rate is high between the ages of 6 to 24 months because breast milk alone is not enough to meet the nutritional needs of the child and other foods are introduced to fulfill them [13]. WHO defines complementary feeding as the process starting when breast milk alone is no longer sufficient to meet the nutritional requirements of infants, and therefore other foods and liquids are needed, along with breast milk*.*[14] While some studies indicate that complementary feeding can be introduced at 4 months, WHO recommends that children should be introduced to complementary foods at 6 months [15–17].

In the recent times there has been renewed interest in preventing malnutrition. A review of studies on interventions for child under nutrition and survival, it is observed that promotion of breastfeeding, behavior change communication for improved complementary feeding, zinc supplementation, Vitamin A fortification, handwashing and treatment of severe acute malnutrition are effective to reduce child under nutrition. While other interventions for which evidence show little effect include Vitamin D supplements, iodine supplements, cooking in iron pots, preschool feeding programs and growth monitoring [18]. Existing intervention studies on nutritional education have involved and trained community workers in the first step of intervention. These trained health workers then counseled the caregivers regarding feeding of the children [19–22]. These studies have reported significant improvement in growth and feeding practices mainly in rural settings. Only few such studies are available, that are conducted in peri-urban setting or among migrants [19]. Another issue which needs to be seen is the sustainability of such efforts in the community. From the sustainability point of view it is important to integrate such activities/interventions with existing health services. Also it is important to consider the factors that will enhance the accessibility and affordability of quality home foods to the children especially those belonging to vulnerable groups of the society. This study is planned to determine the effectiveness of a culturally appropriate nutritional education intervention that can be delivered through health services to improve complementary feeding of infants 6 months to 12 months in Chandigarh, North India, so as to prevent malnutrition in infants.

**REVIEW OF LITERATURE**

Review of literature was done by searching the search engines at PubMed, Clinical key, Researchgate, and Google scholar using the keywords as “nutritional intervention”, “educational intervention”, “intervention study”, “infants”, “complementary feeding”, “feeding practices”.

**Definition and causes of under nutrition**

Under nutrition is defined as the outcome of insufficient food intake and repeated infectious diseases. It includes being underweight for one’s age, too short for one’s age (stunted), dangerously thin for one’s height (wasted) and deficient in vitamins and minerals

(micronutrient malnutrition) [23]. Malnutrition is self perpetuating. It is an important cause of childhood morbidity and mortality, and leads to permanent impairment of physical and possibly mental growth of those children who survive. The nutritional status of a child at any point of time depends on his or her past nutritional history. To some extent this nutritional history is linked to the mother’s health and nutritional status [24].

According to UNICEF, the main causes of childhood malnutrition can be categorized as; household food insecurity, inadequate care and unhealthy household environment, and lack of health care services [25]. There are many other contributing factors in the web of causation, viz. poor environmental conditions, large family size, poor maternal health, failure of lactation, premature termination of breast feeding, adverse cultural practices related to child rearing and weaning such as the over use of diluted cow’s milk and discarding cooking water from cereals and delayed supplementary feeding [26].

One of the major causes of malnutrition in young children in developing countries is poor feeding practices such as: introducing complimentary foods at an early or late stage, restriction in food selection and giving children poor quality and insufficient amounts of complimentary foods [17,20,27,28]. It is argued that poor feeding practices are associated with caregivers poor knowledge, lack of information and their being restricted by traditional beliefs [21]. Poverty is another major cause and affects food choices [2]. With poverty, caregivers tend to give children the food that is available regardless of its nutrition value [29]. Compared to the richest children, the poorest children are 1.9 times more likely to die before age 5. The urban poor living in slums face different challenges as compared to their rural counterparts. The slum dwellers are very vulnerable to price increases as they are almost exclusively dependent on the market for food and other items. Although it is considered that urban areas have better healthcare, education and sanitation, evidence reports that access to these services is not easy for urban poor. Significant challenges are faced in urban food security and nutrition programming (by government, UN and NGOs), beginning with assessment and targeting issues when faced with a highly mobile, densely packed population, where in- and out-migration is a constant feature. Social protection and cash transfer programs are promising approaches in urban areas, with evidence to suggest that they improve dietary diversity, but as yet there remains a lack of evidence of their impact on the nutritional status of children under 5 years of age [30].

**Problem statement**

***Developed countries***

In developed countries, over nutrition is more common than under nutrition. In United States of America,1.2 % of children below 5 years were stunted, 0.7% were wasted while 7.2% were obese in the year 2012 [31]. Many federal food assistance programs are run for malnourished individuals in need of assistance e.g. Expanded food and nutrition education program and Temporary emergency food assistance program in USA [32].

***Developing countries***

About 178 million children aged younger than 5 years who were stunted, most were living in South Asia and sub-Saharan Africa. About 90% of these stunted children were living in just 36 developing countries [33]. Globally about 80% of the child deaths occurred in South Asia and sub-Saharan Africa in 2015, and almost half occurred in just five countries: India, Ethiopia, Nigeria, Pakistan and the Democratic Republic of Congo. In these countries malnutrition is an important public health problem and nutrition interventions can save the life of millions of children [34].

***India***

India has higher stunting rates than some of its South Asian neighbours like Sri Lanka, Maldives, Vietnam and Bangladesh. Most of the irreversible damage due to malnutrition in India happens during pregnancy and in the first 2 years of life [4]. As per National Family Health Survey (NFHS) 3 data; 44.9% of Indian children, less than 3 years of age were stunted, 40.4% were underweight, and 22.9% of children had wasting [7].

In Chandigarh, 27.5% of children less than 5 years of age were stunted, 23.0% were underweight and 21.0% of children had wasting [35]. A study conducted by Thakur JS et al to ascertain decadal trend in childhood nutritional status between 1997 and 2007 in Chandigarh and to assess the impact of Integrated Child Development Services (ICDS) on childhood undernutrition reported that the prevalence of underweight among under-five children remained almost stagnant in the last one decade from 51.6% (1997) to 50.4% (2007) and an insignificant difference (p=0.3)was observed in prevalence of underweight among children registered under ICDS program (52.1%) and those not registered (48.4%) in 2007 in Chandigarh [36].

**Intervention strategies**

It is against this background that numerous strategies have been put in place to reduce the prevalence rate of malnutrition in children. These strategies have been tested in 36 countries which has 90% of the stunted children of the world [18]. These strategies are: promotion of breast feeding [37], behaviour change communication for improved complementary feeding [19–21,38], WHO recommended treatment of severe acute alnutrition[39], and provision of Vitamin A [40] and Zinc supplementation [41] as shown in table 1. Conditional cash transfer programs for nutrition education in Latin American countries have been successful [42,43]. Dietary diversification strategy has been proven to be successful at small scale only [44,45]. To combat malnutrition it has been recommended by many researchers that nutrition intervention should be accessible, sustainable and culturally sensitive and integrated with local resources [19].

**Table1: Interventions for child undernutrition** [18]

| **Sufficient evidence for implementation in all 36 countries** | **Evidence for implementation in specific situational contexts** | **Interventions with insufficient or variable evidence of effectveness** | **Interventions for which evidence showed little or no effect** |
| --- | --- | --- | --- |
| Promotion of  breastfeeding  (individual and group  counselling).  communication for  improved  complementary  feeding (additional  food supplements in  food insecure  population).  Zinc  supplementation.  Zinc in management  of diarrhoea.  Vitamin A  fortification or  supplementation.  Universal salt  iodisation.  Hand washing or  hygiene  interventions.  Treatment of severe  acute malnutrition. | Conditional cash  transfer programmes  (with nutritional  education).  Iron fortification and  supplementation  programmes.  Insecticide-treated  bed nets. | Dietary  diversification  strategies.  Small animal  gardening.  Iodine supplements  Cooking in iron pots. | Growth monitoring.  Vitamin D  supplements.  Preschool feeding  programmes. |

A systemic review conducted by Black RE et al (2013) emphasises the integration of nutrition interventions with improvements in health and hygiene behaviour and other nutrition sensitive interventions. It also reinforces the evidence for behaviour change interventions (BCI) in improving complementary feeding practices and linear growth [33].

Culture is defined as the way of life, especially the general customs and beliefs, of a particular group of people at a particular time [46]. Culturally appropriate food items are those that are most commonly found within a particular culture e.g. for religious and/or socio-economic reasons, many South Asians follow a vegetarian diet which focuses on balanced staples such as mung beans (dal), lentils, potatoes, rice (long-grained basmati), millet, and pulses (chickpeas, beans, peas). Caregivers might not make the best use of available resources because of cultural beliefs and practices, lack of knowledge of the best foods for young children even when available in the home, and inappropriate advice [47,48]. In these circumstances, interventions that provide additional complementary food can prevent growth retardation especially for children 6–12 months old [49,50]. Large-scale educational interventions have also been effective in changing the way caregivers give food, increasing dietary intake and in improving child growth [49].

Integrated Child Development Services (ICDS) program is the biggest nutrition supplementation program in India which was started in 1975. For young children, this program aims to provide supplementary feeding, preschool education, monitor child growth and some basic health services. But due to regressive placement, irregular funding, poor supervision and inadequate training of staff, the program has made little impact on child nutrition [8]. Integrated management of neonatal and childhood illness (IMNCI) was another strategy for management of childhood illness including malnutrition which utilized community health workers. From 2005 – 2009 more than 2 lakhs community health workers were trained in IMNCI. Health workers visited more newborns but focused on weighing and diagnosing common childhood illnesses, counseling, and feedback regarding malnutrition remains neglected. Also poor supervision of workers and inadequate essential supplies has affected the performance of trained workers [10]. Auxiliary nurse midwives, accredited social health activist and *anganwadi* workers are important community workers who provide nutrition education regarding complementary feeding. It is. provided at community outreach level during routine immunisation sessions, IMNCI/ sick child consultation and village health and nutrition day. Village health, sanitation and nutrition committees (VHSNC) are an important platform to promote sanitation and educate community members on nutritional issues. But a study conducted in Orissa and Jharkhand reported that community health and nutrition workers focused on sanitation and record keeping, rather than on malnutrition. Regarding nutrition, the main activity was keeping record of malnourished children and referring them to hospital [11] In 2013, Guidelines for enhancing optimal infant and young child feeding practices (IYCF) were introduced, but a study conductedin Gujarat to assess the knowledge of *anganwadi* workers regarding infant and young child feeding practices, and their ability to counsel and influence caregivers regarding these practices, reported that a huge gap existed between the *anganwadi* workers' knowledge and their ability to apply this in formal counseling sessions with caregivers. They were not able to engage with caregivers and had poor active listening skills. Also they were not taking the feeding history of childrenand were unable to provide need-based advice [12].

In a study conducted in China, village nutritional educators were trained to conduct monthly growth monitoring and complementary feeding counselling visits to all pregnant women and families with infants born during intervention. The education group infants were significantly heavier and longer at 12 months (weight for age -1.17 vs. -1.93, height for age - 1.32 vs. -1.96) as compared to control group [21].

Penny ME et al conducted a study in Peru to see the effectiveness of an educational intervention delivered through health services, where health service providers were trained to provide simple, standardised age appropriate messages for caregivers of young children. It was found that the children in intervention group gained 295 gmoreweight than the children in the control group and they were 1 cm taller than the children in the control group at the end of eighteen months of age. Caregivers in the intervention area were more likely to report receiving nutrition advice from the health service than the control group (52% vs. 24%) [19].

Roy SK et al conducted a similar study in Bangladesh using culturally appropriate nutritional education package. Nutrition education given every week for first 3 months and then biweekly for next 3 months with the help of community based health workers. This study reported that the intervention group had a higher weight gain than the control group after the end of the six month intervention (0.86 vs. 0.77kg, p = 0.053) and after the end of the one year observation period (1.81 vs. 1.39 kg, p < .001) [22].

A study conducted in Vietnam used the positive deviance approach to improve the feeding behaviour of malnourished children by conducting intensive nutrition rehabilitation sessions. Two weeks long daily nutrition education sessions were conducted every month for nine months. Growth monitoring and promotion sessions were conducted every month for two years. It resulted in better eating behaviour of intervention children (20% or 70 g more food per day) as compared to control children. Nearly 40% more intervention than control children met their energy requirements [38].

Zaman S et al conducted a study in Pakistan to see the effect of complementary feeding counselling of health workers and its influence on maternal behaviour and child growth. Lady health visitors (LHV) were trained in IMNCI. Mother-child pairs were visited at home within two weeks, 45 days, and 180 days after recruitment. The communication skills and consultation performance of health workers were significantly better in the intervention group than in the control group. The mothers' recall of the recommendation of health workers and reported infant-feeding practices were also significantly better in the intervention group. Growth faltering was less in the intervention group, with the largest effect observed among children in the age-group of 12 + months [51].

In a study conducted in Tando Jam and Quetta, Pakistan, mothers were given nutritional counselling which focussed on five messages for children 6months to one year of age. Nearly 36% children in Tando Jam and 32% children in Quetta progressed to a normal nutritional status with a significant increase in the number of meals taken per day (Tando Jam— ≤ 0.000/Quetta— ≤ 0.025). However, this study was conducted for a short duration of time. There is a need to assess the long term changes in the nutritional status and dietary habits of the study population [52].

Many studies have been conducted in India to show that nutritional education can improve complementary feeding practices.

In a study conducted in Haryana, health and nutrition workers in the intervention community were trained to counsel on locally developed feeding recommendations. There was a small but significant effect on length gain in the intervention group (difference in means=0.32 cm) and more in male infants (difference in mean length gain=0.51 cm).Hence concluding that improving complementary feeding practices through existing services is feasible but the effect on physical growth is limited [20].

Kushwaha KP et al conducted a study in Lalitpur, India to see the effect of mother support groups on complementary feeding practices. This study was conducted over a period of five years. It was found that initiation of complementary feeding (6–8 months) was 85% vs. 54% and complementary feeding along with continued breast feeding up to 2 years of age was 36% vs. 4.5% as compared to pre-intervention period [53].

In a study conducted in Karnataka, locally trained counsellors delivered monthly nutrition education to caregivers of 5 – 11 months old infants. Improvement was found in weight velocity for female infants in the intervention groups. Theinfants were also more likely to exhibit at least four positive feeding behaviors--intervention infants had a higher mean daily feeding frequency (more likely to be fed solids at least four times a day (OR = 4.35, 95% CI = 1.96, 10.00), higher dietary diversity (more likely to receive a more diverse diet OR = 3.23, 95% CI = 1.28, 7.69), and were more likely to be fed foods suggested by the counselors such as bananas (OR = 10.00, 95% = 2.78, 33.3) compared to non-intervention infants [54]. Rao S et al studied the complementary feeding practices among mothers of children aged six months to two yearsin Karnataka in 2011. It was found that 77.5% mothers had started complementary feeding at the recommended time of six months but only 32% of mothers were giving an adequatequantity of complementary feeds. The association of initiation of complementary feeding with socio-economic status, birth order, place of delivery and maternal education was found to be statistically significant [55].

**Table** **2: Summary of literature about intervention studies for child** **undernutrition.**

| **Author** | **Year of publication** | **Place** | **Methodology** | **Findings** | **Gaps/ limitations** |
| --- | --- | --- | --- | --- | --- |
| Gulden GS, et al[21] | 2000 | China | Quasi-experimental study.  495 children were enrolled at 4–12 months.  Monthly growth monitoring and counseling by community-based/Village nutrition educator.  Pre-post 1-year program. | The education group infants were significantly heavier and longer at 12 months (weight for age -1.17 vs. -1.93, height for age -1.32 vs. -1.96) as compared to control group. | Weak supervision of community based village educators.  No baseline assessment was done in the study. |
| Pachón H, et al[38] | 2002 | Vietnam | Randomized effectiveness trial.  238 children age 5–25 months were enrolled.  2 weeks long daily nutrition education sessions per month for nine months.  Monitoring of growth was done monthly. | Intervention children consumed 20% (70 g) more food per day as compared to control children at 12 months.  More intervention children(40%) had met energy requirement. | Amount of food served to children was not taken into account and latest energy values of foods were not calculated. |
| Bhandari N, et al[20] | 2004 | Haryana, India | Randomized control trial  1025 newborns were enrolled  Nutrition counseling at monthly home visit for 12 months by ANM, AWW and HW.  Pre-post 18-month program. | Small but significant increase in length was seen intervention group (0.32cm, 0.03).More effect in male infants(0.51 cm, 95% CI 0.03, 0.98). no effect on weight.. Signiﬁcantly more energy intake was found in the nine month old infants in intervention group. | As the study was funded, so it may be difficult to replicate it in a resource constrained circumstances. |
| Penny ME, et al[19] | 2005 | Peru | Randomized Control Trial.  Birth cohort of 377 infants were enrolled and followed up from 0-18 months of age.  Intervention constituting of nutrition education was delivered through health services through by nutritional counseling. | Children in intervention group gained 295 g weight and they were 1 cm taller control group at 18 months of age. More caregivers in the intervention area reported that they received nutrition advice from the health workers than the control group (52% vs.. 24%). | This study may be difficult to replicate in areas where access to food is a limiting factor. |
| Kilaru A, et al[54] | 2005 | India | Effectiveness study  242 infants age 5-11 months were enrolled.  Nutrition education was delivered every month to caregivers by locally trained counsellors | Velocity of weight of female infants was more in the intervention group.  They had a morefrequency of feeding, more diverse diets. They had more chances to be fed complementary foods. | Assessment of diet was not done.  Field research staff was specially recruited for the study purpose only, hence the effect may not be sustainable in community. |
| Roy SK, et al[22] | 2007 | Bangladesh | Randomized control trial.  605 children age 6–9 months were enrolled in community nutrition centres.  Nutrition education given weekly for 3 months and then biweekly for 3 months  with the help of community based health workers.  6-month follow-up | Infants in the intervention group gained more weight than the control group after six month intervention (0.86 vs. 0.77kg, p = 0.053) and after one year observation period (1.81 vs. 1.39 kg, p < .001) | As the study was funded, so it may be difficult to replicate it in a resource constrained circumstances. |
| Zaman S, et al[51] | 2008 | Pakistan | Randomized control trial  375 children at the age of 6 to 24 months were enrolled.  5 1⁄2 days training for LHV using IMCI training, they visited the mother- and child pairs at home within two weeks, then at 45 days, and after 180 days of enrollment | More intervention mothers offered animal foods to their children (p=0.03 and 0.01). At the 3^rd^ visit, the mean SD score for WAZ more in intervention group (−1.17 vs. −1.7, p=0.012) | Only 58% of the trained LHV could perform adequately on observation; most mothers in the intervention group received only one counseling session. |
| Rao S, et al[55] | 2011 | India | Cross sectional study  Mothers of children 6 months – 2 years.  Semi structured questionnaires.  Study duration – 2 months. | Complementary feeding was started by 77.5% mothers had started at six months but complementary feeds was given by only 32% of mothers in adequate amounts. | Hospital based study so it can’t be used to represent the value in the general population.  Recall bias may be more.  The feed consistency was not taken into account. |
| Khan ZA, et al[52] | 2013 | Pakistan | Intervention study.  586 children between 6 months – 8 years of age.  Intervention strategy-nutrition education (individual counseling sessions targeting the mothers) | About 36% children in Tando Jam and 32% children in Quetta gained normal status of nutrition. Significant increase in the number of meals was observed. (Tando Jam-𝑃≤0.000/Quetta-𝑃≤0.025). | Short period of intervention.  Long term changes should be assessed in the population. |
| Kushwaha KP, et al[53] | 2014 | India | Quasi experimental study conducted over 5 year period.  Mother infant pairs were recruited.  Mother support groups provided counseling and support to the mothers. | Complementary feeding was started at the age of 6–8 months in 85% vs. 54% 2 years old children and in 5 year olds was 96% vs. 54%.  Complementary feeding along with continued breast feeding up to 2 years of age at both 2 years (36% vs. 4.5%); and 5 years (42% vs. 4.5%) as compared to pre-intervention period. | Project was funded so it may be difficult to replicate in resource constrained setting. |

Most of the studies included formative research but there was no pilot testing of the intervention in these studies [19–22]. They involved community health workers and nutritionist as facilitators of intervention but primary caregivers were not directly trained. The health workers working in public health sector has been used in few studies [19,20]. It is our understanding that if community workers are involved in delivering the intervention, then the benefit will reach more people and intervention will likely to be sustainable in a community. None of the studies conducted in India have explored the concept of tracking for undernutrition among children. There is lack of intervention studies on improving feeding practices and undernutrition in children among migrants in India. Since migrant population is on the rise in the country [56], and they are most vulnerable population especially their children in terms of higher morbidity and mortality, hence it is essential to focus on this group to prevent the most common underlying cause of morbidity and mortality among these children i.e., malnutrition. The present study is planned to assess the effectiveness of a nutritional education intervention by involving the primary care givers/mothers and health workers catering to vulnerable and migrant population in Chandigarh. The health workers will use the indivisualized approach and provide culturally appropriate nutritional counselling at home and support the mothers in infant feeding practices. The growth monitoring will done every month through monthly work plan for ANMs and *anganwadi* workers, so that they can focus on mother-infant dyads with low infant growths.

**Research question**

Whether culturally appropriate nutrition educational intervention delivered through health services can improve growth and complementary feeding of infants of age 6 months to 1 year old in Chandigarh?

**Null Hypothesis**

Culturally appropriate nutrition educational intervention delivered through health services has no effect on improving growth and complementary feeding of infants of age 6 months to 1 year old in Chandigarh.

**Aim**

To assess the effectiveness of culturally appropriate nutrition educational intervention delivered through health services to improve growth and complementary feeding by infants of age 6 months to 1 year old in Chandigarh.

**Objectives**

- To ascertain the effectiveness of a culturally appropriate nutritional education intervention delivered through health services to improve the growth and complementary feeding of infants of age 6 to 12 months in Chandigarh.
- To develop a child undernutrition tracking module for health workers to monitor child growth.

**METHODOLOGY**

**Study area:**

This study will be conducted in Chandigarh, Union territory. The population of Chandigarh is 1,054,686 with 1,19,434 children below the age of 6 years as per 2011 census.[57] About 97% of population resides in urban area. Out of which about 30% of the population resides in resettlement colonies. The female literacy rate is 81%.[57]

**Study design**

The study design will be quasi-experimental. The study and control area will be selected purposively. Study area will be Burail, an urbanized village in the catchment area of Civil Hospital, sector 45, Chandigarh. It is also one of the field practice area of department of Community Medicine, School of Public Health, PGIMER, Chandigarh. The population of Burail is 47,869 with 862 children below 1 year of age, as per annual health survey report, 2015-16. Most of the residents are migrants from neighboring states like UP, Bihar, Uttarakhand, Haryana and Himachal Pradesh, and belong to low or middle socio-economic groups. About 97% of children aged between 12 to 23 months in Burail are fully immunized, as per annual health survey report, 2015-16. Burail has 7 auxiliary nurse midwives (ANMs) and 19 anganwadi workers and centers. The control area will be Maloya, another urbanized village in Chandigarh. The background characteristics of control area are similar to study area in terms of vulnerability, migration of population, geographical location, socioeconomic status and baseline child health status. It has a population of 28,176 with 483children below 1 year of age as per annual health survey report 2015-16 of Civil Dispensary, Maloya. Maloya has 3 ANMs, 1 ASHA, 26 *anganwadi* workers and centers. About 90% of children aged 12-23 months are fully immunized in this area as per annual health survey 2015-16.

**Study population**

The study population will be mother-infant dyad with infant in age group 4-6 months at enrollment;auxiliary nurse midwives (ANM) and *anganwadi* workers (AWW) in the study and control area.

**Inclusion criteria**

1. Mother-infant dyad with age of infant between 4 to 6months of age.
2. Parents who are residing in the study area for the past 6 months and have no plans to migrate during the intervention period.
3. Mothers, ANMs and *anganwadi* workers who will give consent to be the part of the study.

**Exclusion criteria**

1. Severely ill infant or infant having clinical complications requiring hospitalization.
2. Infants with cerebral palsy or other congenital anomalies/malformations.

**Sample size:**

The sample size for the study is calculated by using the formula = (u+v)²*(σ₁²+σ₀²)/(µ₁-µ_0_)²

where

u=two sided percentage point of normal distribution corresponding to 100% - power=1.28

v=percentage point of normal distribution corresponding to (two sided) significance level=1.96, assuming 80% power with 95% C.I. [58].

(µ₁-µ₂)= difference between the means

d₁, d₀ = standard deviations

Difference between the mean weights is assumed to be 250 g [20].

Sample size thus calculated to be= [(1.28+1.96)²*((0.65)^2^+ (0.65)²]/ (0.25)² = 182. This is the minimum sample size of infants to be included in the study per arm.

Considering attrition rate of 10%, sample size per arm = 202.

**Sampling Technique**

A list of all mother-infant dyads, where age of infants will be between 4 to 6 months, will be obtained from the *anganwadi* centers of the study and control area. Mother-infant dyads will be selected by simple random sampling in study and control groups from the complete list. Final inclusion will be done for those mother-infant dyads who will meet the study criteria. In the first month of enrolment, all mother-infants dyads between four to six months old infant will be enrolled. Enrollment will be continued thereafter till the required sample size is achieved in both study and control groups.

**Study duration**

1 year (July 2016- June 2017)

**The study will be conducted in four phases:**

1. **Pre-intervention phase**

***Formative research***

Focus group discussions (FGDs)will be conducted separately with each group of mothers, ANMs, AWWs and community representatives to explore the culturally appropriate foods for infants, gap between knowledge and action of mothers, frequency of infant feeding, feeding practices etc, using FGD guide, till data saturation (Annexure 1). Result of the FGDs will help in the modifying the nutritional education intervention as per the need of mothers and health workers, and development of child undernutrition tracking module for monitoring and supervision of child growth by health workers. Pilot testing of the intervention will be conducted in an area similar to the study area but not in study area.

1. **Baseline Assessment**

Baseline assessment of maternal knowledge of infant feeding, infant feeding practices by 24 hour dietary recall (frequency of feeding, number of meals and snacks per day) and hand hygiene practices will be done by using a pretested semi-structured questionnaire (Annexure 2). Feeding on the day before the interview will be used to represent the infant feeding practices. The status of under-nutrition will be assessed by baseline anthropometry of the infants by using Digital Salter Model 235, CMS weighing equipment and stadiometer (SECA height board UNICEF) for measuring weight for age, weight for length and length for age [59,60]. The knowledge of ANM and *anganwadi* workers on infant feeding and status of growth monitoring will be checked by a pre tested semi-structured questionnaire (Annexure 3). It will be assessed in both study and intervention arm.

**3. Intervention Phase**

***a. Intervention for mothers/ caregivers:***

The intervention will be delivered in community settings at mother-infant dyad level. The intervention duration will be six months. Mothers/caregivers will be provided nutritional education as per the modules.

**Module for mothers**: The content is consistent with age specific feeding recommendations made by World Health Organization, 2001 [61]. Culturally appropriate food recipes will be standardized and delivered with the help of dietician (co-guide).

**Nutritional Education & counseling** (for mothers) – Two sessions of nutrition education and counseling will be conducted for the mothers/caregivers.

- 1^st^sessionwill be conducted when the infants will be 6-8 months old.
- 2^nd^ session will be conducted when the infants will be 9-11 months old.

Both the sessions will be centered on feeding of infants, maintaining hygiene while cooking and eating and sanitation. Each session will last 30-45 minutes and will be conducted for 20-30 participants. Talks, group discussion and demonstration will be used to impart knowledge and skills of mothers related to infant and child feeding.

The control group will continue to receive standard care through routine health education offered by ANMs and *anganwadi* workers.

***b. Intervention for health workers***

**Module for Health workers –** The module will have three parts and will be delivered as:

- Part 1--- orientation and introduction
- Part 2 --- communication skills
- Part 3 --- home visit tasks and procedures

The ANMs will be trained as nutrition counselors. They will receive training at baseline and the session will be repeated after 2 months. After the training, the ANMs will conduct a fortnightly home visits to counsel and support mothers for infant feeding practices. Supervisory visits will be conducted by the resident every two months to assess their work.

A sensitization meeting with families, ANMs and AWWs responsible for child health will be conducted before the training of mothers and another meeting will be conducted after two months. ANMs/ AWWs will visit the mothers/care givers in their homes fortnightly/monthly. They will prepare an individualized plan of each mother-infant dyad for infant growth based upon the family’s acceptability of food and affordability. They will observe how mother is feeding the baby, hand hygiene practices. They will accordingly counsel them and improve the feeding practices. They will also resolve any problem mothers are facing while feeding the baby. They will also organize the monthly group meetings of the mothers. In these meetings mothers will get a chance to learn from the peers whose child are showing weight gain and will be motivated through positive deviance model[38].

1. ***Supervision of health workers***
   1. A module will be developed for tracking of undernourished child. The infants will be weighed every month in the *anganwadi* centers. The anthropometric data of the infants will be entered in an excel sheet in computer and z score will be calculated. A monthly work plan showing the nutritional status will be prepared for each child in the study area. This work plan will be given to all the ANMs and *anganwadi* workers in the area, so that they can focus on mother-infant dyads with low infant growth.
   2. About 10% of the counseling sessions by the health workers with the mother-infant dyads in the field will be directly observed and supervised by the junior resident in the study area, and accordingly feedback to the health workers and mothers will be given.

**4. Post-intervention phase**

End line assessment of maternal knowledge of infant feeding and infant feeding practices, 24 hour dietary recall (consuming foods from 4 or more food groups, recommended number of meals and snacks per day) will be done by using the same pretested semi-structured questionnaire as was used in baseline assessment in study and control area (Annexure 2). The status of undernutrition will be assessed by anthropometry of the infant (wt/age, wt/length, length/age). Similarly, the knowledge of ANMs and *anganwadi* workers on infant feeding and status of growth monitoring will be checked by the same pretested semi-structured questionnaire (Annexure 3) in both intervention and control arm.

**Study Tools**

1. Focus group discussion will be conducted with mothers/ANMs/AWWs/Community representatives to explore the culturally appropriate foods, gap between knowledge and action of mothers, frequency of feeding etc using focus group discussion guide (Annexure 1).
2. Pretested semi-structured questionnaire will be administered to the mothers (Annexure 2) participating in the study to assess their baseline and post-intervention knowledge of infant feeding, infant feeding practices e.g. number of meals and snacks consumed per day and hand hygiene practices. 24 hour dietary recall form will be used to assess the infant feeding. The proportion of children consuming recommended number of semi-solid/soft meals and snacks per day according to WHO will be determined at baseline and 6 months in intervention and control groups. The infant’s age will be recorded from the mother and child health card or birth certificate.
3. Pretested semi-structured questionnaire will be administered to the ANMs/AWWs (Annexure 3) participating in the study to assess their baseline and post-intervention knowledge of infant feeding practices and advices given to mothers/ caregivers.
4. Separate training modules will be designed for mothers (Annexure 5) and health workers (Annexure 4). The content of mother’s training module is consistent with age specific feeding recommendations made by World Health Organization, 2001 and Complementary Feeding Counseling: a training course made by World Health Organization, 2004 [61,62]. The content of ANMs and AWWs training module is consistent with Complementary Feeding Counseling: a training course made by World Health Organization, 2004 [62].
5. Anthropometric measurements will be done by standardized weighing scale (Digital Salter Model 235, CMS weighing equipment) and stadiometer (SECA height board UNICEF) [59,60]. The nutritional status of the children will be assessed by plotting the weight and height of the children on World Health Organization (WHO) 2006 Growth Standards growth charts using z-scores. Children falling below the −2 standard deviation (SD) cut-off would be considered as underweight (weight for age), stunted (height for age), and wasted (weight for height).

**Data Collection**

Data on anthropometry will be collected at baseline and 6 months of intervention in both study and control group. Data collection will be done by the chief investigator who is a junior resident in the Department of Community Medicine. After data collection, all filled forms will be manually checked for completeness and consistency.

**Outcome measures**

**Primary outcome**

- Mean change in the weight for age.

**Secondary outcome:**

- Mean change in the weight for height.
- Mean change in the Height for age.
- Change in level of maternal knowledge and practice of recommended infant and child feeding practice following intervention.
- Change in proportion of infants who were consuming foods from four or more food groups
- Change in proportion of infants who were consuming the recommended number of meals and snacks per day

**Statistical Analysis**

The data will be entered in Microsoft excel version 7. The data will be presented in frequencies, proportion and means. Statistical analysis will be done by using Statistical Package for Social Sciences version 16. T-test will be used to assess level of significance in difference between the means of the group. Chi square test will be done to assess significance of difference between proportions. Effectiveness will be measured by calculating the difference in difference in mean change in weight between the intervention and control group. Repeated measure ANOVA will be used to assess the difference in mean change in weight in the intervention group at baseline, 3 months and 6 months post intervention.

**Ethical Issues**

The approval for the study will be taken from the Institutional Ethics committee of PGIMER, Chandigarh. A written informed consent will be obtained from parents of infants and AWW/ANMs prior to their recruitment in the study (Annexure 6). Participant information sheet (Annexure 7) will be provided to all the participants of the study. Confidentiality of study subjects will be maintained. The parent/legal guardian/ANM/AWW will be given the right to remove their child from the study or to withdraw consent to their participation in the study, at any time without reprisal.

**ANNEXURE 1**

**Focus Group Discussion Guide for Mothers/ Caregivers**

Title: Effectiveness of a culturally appropriate nutrition educational intervention delivered through health services to improve growth and complementary feeding of 6 months to 1 year old infants in Chandigarh: a quasi experimental study.

Name of institute: Post graduate institute of medical research and training (PGIMER)

Estimated time: 90 minutes at maximum

**Objectives:**

1. To explore current practice, barriers, facilitators on complementary feeding (CF) from perspectives of mothers/ ANMs

2. To explore the culturally appropriate foods and foods that is available to families in your area.

3. To explore the knowledge and attitude of mothers/ANM regarding complementary feeding.

4. To identify the role/influence of the family members, especially grandmother and husband on decision making of the mother to give appropriate complementary foods.

5. To identify potential communication channels for infant and young child feeding.

**Subjects:**

A group of 8-10 mothers/ caregivers / 3-4 ANMs/ 2-3 community representatives will be conveniently selected from the community. Age group difference, economic conditions (poor and non-poor) and different children groups (6-8 months and 9-11 months) will be considered to select purposive participants. In total, 2 FGDs with the participants will be done.

Materials: Pieces of colored paper, different colored pens, audio recorders/ video recorders.

**Methods:**

The focus group discussion will be facilitated by the researcher who is the junior resident and a note-taker. The discussion will be audio recorded/video-recorded if participants agree to do so. Free listing and ranking will be applied to identify the common benefits, disadvantages, roles of family members etc. The following topics will be explored:

- Culturally appropriate foods and foods that is available to families in your area.
- gap between knowledge and action of mothers
- frequency of feeding etc.

**FGD for mother included the following discussion points:**

1. At what age should the mother start to feed her child foods other than breastmilk? Why?
2. What are the current feeding practices of your child?
3. What are the culturally appropriate foods available in your area?
4. Can you tell us what foods are appropriate to give children 6-12 months? And why?

PROBE: any foods including 1) cereals (rice), 2) meat, fish, egg, 3) fruits (banana, mango), 4) vegetable (saag, tomato, gobi), 5) liquids like (tea, milk)

1. Is there any difference between what you would give to children 6-8 months old and what you would give to children 9-12 months old? (E.g.: daal ka pani for the former and khichadi, dalia for the latter)
2. What foods are NOT good/appropriate for children 6-12 months? And why? ( not good taste, take time to prepare, cook, feed the child…….)
3. What is the support you have to feed your child?

(PROBE: preparing, cooking, feeding the child, encourage the child to eat)

1. Who else in the family supports the mother to feed her child? (FREE LISTING then RANKING who is the most supportive (PROBE: the father, the grandfather, cousin, neighbour)

**FGD for health workers included the following discussion points:**

1. What are the factors that contribute to under nutrition among children in this community?
2. What are the current practices, barriers, facilitators and influencers complementary feeding (CF) in the community ?
3. At what age should the mother start to feed her child foods other than breastmilk? Why?
4. What are the culturally appropriate foods available in your area?
5. Can you tell us what foods are good / culturally appropriate to give children 6-12 months? And why?
6. Is there any difference between what you would give to children 6-8 months old and what you would give to children 9-12 months old?
7. What foods are NOT good/appropriate for children 6-12 months? And why?
8. How do you support the mother to feed her child? What more can you do to support the mother? FREE LISTING (PROBE: preparing, cooking, instructing, demonstrating the mother to feed the child, feeding the child, encourage the child to eat)
9. How can you improve the complementary feeding practices in the community?

Also, after an issue has been discussed, a summarization will be done to ensure that all opinions surrounding the issue have been covered.

**ANNEXURE 2**

**Questionnaire for mother-infant dyad**

Title: Effectiveness of a culturally appropriate nutrition educational intervention delivered through health services to improve growth and complementary feeding of 6 months to 1 year old infants in Chandigarh: a quasi experimental study.

**Institute: Department of Community Medicine, PGIMER**

**I. Basic information of mother/ caregiver:**

1. Unique ID no. …………………………
2. Name - ……………………………..
3. Age(years) - ………………………………
4. Sex: 1= M, 2=F
5. Address - ……………………………………………………..
6. Area: 1. Urban 2. Rural 3. Slum
7. Phone no. ……………………………….
8. Religion- 1. Hindu 2. Muslim 3. Sikh 4 Christian 5. Others.
9. Caste – 1.Gen 2. SC 3. ST 4. OBC 5. others
10. Education- 1/2/3/4/5/6/7
11. Occupation - 1/2/3/4/5
12. Monthly income of the mother:…………..
13. Marital status – 1/2/3/4/5/6
14. If mother is working who will take care of the baby ………….( 1 = in laws, 2 = crèche, 3 = others specify …………………. 4.By self )
15. Type of family- 1. Nuclear family 2. Joint family 3. Three generation family 4. Broken family 5. Other specify………

Family profile –

| Serial no. | Name | Age | Sex | Relationship with the head | Education | Occupation | Monthly income |
| --- | --- | --- | --- | --- | --- | --- | --- |
|  |  |  |  |  |  |  |  |
|  |  |  |  |  |  |  |  |
|  |  |  |  |  |  |  |  |
|  |  |  |  |  |  |  |  |
|  |  |  |  |  |  |  |  |
|  |  |  |  |  |  |  |  |
|  |  |  |  |  |  |  |  |

1. **Relation to family member-** self-1, father-2, mother-3, sister-4, brother-5, grand (father-mother)-6, uncle & aunt-7, other-8
2. **Gender-** Male- 1, Female*-* 2
3. **Education-** illiterate-1, primary school certificate- 2, middle school certificate - 3, High school certificate - 4, Intermediate or post high school diploma-5, Graduate or post graduate - 6, Professional or Honours- 7
4. **Occupation -** 1. Working (Govt./Private) 2. Homemaker 3.selfemployed
    4. Labourer/ maid 5. Student
5. **Marital status-** unmarried-1, married-2, widow-3, separated-4, divorce-5, married but not gauna-6
6. Total income of the family-…………..
7. Kuppuswamy SES score:……………
8. Socioeconomic status (According to modified Kuppuswamy Scale, 2014) – 1.Upper class 2. Upper middle class 3. Lower middle class 4. Upper lower class 5. Lower class
9. Food security score:………….(1= always enough food to eat, 2 = sometimes not enough food to eat, 3 = often not enough to eat)

**II. Child Information sheet**

1. Unique ID no of the child:
2. Name - …………………….
3. Father’s Name - ………………………….
4. D.O.B. - …………………(dd/mm/yy)
5. If date of birth not available age of the baby as per caregiver:………(dd/mm/yy)
6. Completed age at enrolment - …………..months …………days
7. Sex – ……….(1 = M 2 = F)
8. Was the child born at …………( 1= hospital, 2= home, 3=other)
9. Order of birth – 1 / 2 / 3 / 4 / 5 / 6 and above.
10. Birth weight (in Kg) - …………………….
11. Birth:………………(1= Term, 2= Preterm…….Weeks)
12. Immunization status –

| Sr. No. | Vaccine | date of administration of vaccine | age at which vaccine administered | Status- (1= Received/2=not received) |
| --- | --- | --- | --- | --- |
|  | Date of Birth |  |  |  |
| 32. | BCG |  |  |  |
| 33. | OPV-0 |  |  |  |
| 34. | Hep B -0 |  |  |  |
| 35. | DPT1/ Penta1 |  |  |  |
| 36. | DPT2/Penta2 |  |  |  |
| 37. | DPT3/Penta3 |  |  |  |
| 38. | IPV |  |  |  |
| 39. | Rotavirus vaccine |  |  |  |
| 40. | OPV-1 |  |  |  |
| 41. | OPV-2 |  |  |  |
| 42. | OPV-3 |  |  |  |
| 43. | Measles |  |  |  |
| 44. | Vitamin A |  |  |  |

45. Present weight (Kg) -…………………..

1. Present length(cm) - ………………..
2. Malnutrition status: ……………(1= Normal, 2= Moderate Malnutrition, 3= SAM)
3. Wt/age : ………..
4. Wt/ht : ………….
5. Ht/age: ………….

**III. Housing situation:**

1. Living in a rented accommodation: .…{1= Yes , 2 = No (own house), 3= (No, others…..)}
2. Type of house: …………(1=*kutcha*, 2 = *pucca,* 3 = *kutcha-pucca*)
3. No. of rooms ……..(1= 1 room, 2 = 2 rooms, 3 = 3 rooms, 4 = 4 rooms)
4. Overcrowding at home: …..(1= yes, 2 = no)
5. Safe water supply: ………. 1.yes 2.no
6. Main type of cooking fuel used : ……..1. LPG 2. Kerosene stove 3. *Chulha*
7. Sanitary latrine :……….1=yes, 2=no
8. If yes: 1= own toilet, 2 = shared toilet If yes: 1= own toilet, 2 = shared toilet

**IV. Maternal knowledge regarding breastfeeding and complementary feeding practices**

1. Which milk is best for the baby?

1.Breast milk 2.Formula milk 3.Animal milk 4. Don’t know

1. Up to which month exclusive breast feeding should be given?

1. Up to 6 months 2.4-6 months 3.7-8 months 4.Don’t know (998)

1. Can bottle feeding be given to baby? 1.Yes 2.No 3.Don’t know(998)
2. At what age complementary foods (solid, semisolid or soft foods) should be introduced? 1.4-6 months 2.At 6 months 3.7-8 months 4. Don’t know(998)}
3. Name three complementary foods that you think are good for a6-9 month old baby? ………………………………………………………………..(1.Know 2.Don’t Know(998))

(Note: If respondent answer any 3 food items then select option1.Eg: Khichidi, sujikheer, mashed fruits and vegetable, roti, mashed dhal, rice with dal, dhaliya with milk, basen or attacheera etc. If respondent say fruit juice, dhal water, milk then select option 2)

1. How many times a day solid/semi-solid foods should be given to 6-8 months baby? 1.= 3or more times 2. 1-2 times 3.Don’t know (998)
2. What should be the consistency of food given to baby? 1. Thick 2.Thin 3.Very thin
3. Name three snacks that can be given to a 6-8 month old baby? ……………………………………………………(1.Know 2.Don’t Know(998)) (Note: If respondent answer any 3 food items then select option1.Eg: fruits- banana, apple, papaya, mango,chiku,orangeetc, murmura, biscuits. If respondent say kurkure, chocolate, chips, fruity then select option 2)
4. What is the quantity of solid/semi-solid foods your child can consume at his/her age per feed?

| 1. 1-2 teapoonfuls | 1. 1/2 of a 250 ml cup |
| --- | --- |
| 1. 3-5 teapoonfuls | 1. 3/4^th^ of a 250 ml cup |
| 1. 2-3 tablespoonfuls | 1. Full cup |
| 1. 4-5 tablespoonfuls |  |

1. Can tea / sugar drinks/ coffee can be given to your child? 1.Yes 2. No
2. Should ghee or oil be used in complementary foods? 1.Yes 2. No 3.Don’t know (998)
3. Do you know till what age breastfeeding can be continued?
    1.Less than 1 year 2. 1 year - < 2 years 3.Upto& beyond 2 years 4. Don’t know (998)
4. Are commercial baby foods better for infants than homemade foods? 1.Yes 2.No 3.Don’t know (998)
5. Do you need to wash hands with soap and water before feeding the child? 1.yes 2. no
6. If your child is not taking food, should he/she be threatened or bribed?
    1. Yes 2. No 3. Sometimes

**V.Feeding Practices**

***Past***

1. When was breastfeeding initiated after birth?

1.Within 1 hour 2. 1-4 hours 3.4-24 hours 4.After 24 hours 5.Not Given

1. Which food was given first after birth (Pre lacteal feed)?

1.Breast milk 2.Animal milk 3.Standard formula 4.Ghutti 5..Honey 6.Others

1. Did you feed colostrum to the child? 1.Yes 2.No
2. Did your baby receive exclusive breastfeeding (EBF) till 6 months of age? 1.Yes 2.No 3.Baby <6 months of age but on EBF 4.Baby < 6 months but not EBF
3. If No, reason 1.Decreased milk secretion after birth 2. Mother not feeling well/ tired 3.Working mother 4.Baby not satisfied/hungry 5. Prelacteal feed
4. Did your baby receive bottle feeding at any time before 6 months of age? 1.Yes 2. No

***Current***

1. What is the current feeding practice?

1.Only breastfeeding 2.Breast milk + animal milk or formula milk
 3.Breastfeeding+ Complementary feeding 4. Completely weaned

If child below 6 months & answer is option 1 or 2, then skip to question no. 94

1. At what age did you started giving your child solid/semi-solid foods?

1.< 6 months 2. At 6 months 3.7-9 months 4.10 months and above
 5.Not started

1. Type of complementary food being fed to the baby?

1.Cow/Buffalo milk 2.Commercial baby food 3.Homemade semi-solid/solid food 4.Commercial baby food + homemade food 5. Homemade semi-solid/solid food + Cow/Buffalo milk 6. Cow/Buffalo milk + Commercial baby food

7. Homemade semi-solid/solid food + Cow/Buffalo milk + Commercial baby food

1. Is your baby currently receiving bottle feeding? 1.Yes 2. No
2. Is your child eating thick food first at the main meal? 1.Yes 2. No
3. How many times in past 24 hours, your child has taken solid/semi-solid foods? ( 0/ 1 / 2 / 3 / 4 / 5 )
4. What is the quantity of solid/semi-solid foods your child can consume at his/her age per feed?

| - 1. 2-3 tablespoonfuls | - 1. 3-4 tablespoonfuls |
| --- | --- |
| - 1. 4-5 tablespoonfuls | - 1. 2/3^rd^ of a 250 ml cup |
| - 1. 3/4^th^ of a 250 ml cup | - 1. Full cup |
| - 1. Full cup |  |

1. What is the mode of feeding the child? 1.By spoon 2.By hand 3.Both 4.Self by hand/spoon
2. What is consistency of feeds being received by your child? 1.Thick 2.Thin 3.Very thin
3. Are you adding ghee or oil in complementary foods? 1.Yes 2.No
4. Average food group taken daily in last 7 days except milk : 1 / 2 / 3 / 4 / 5

| Food groups | Every day | Weekly thrice | Weekly twice | Never |
| --- | --- | --- | --- | --- |
| **Cereals & Starches, tubers** -Rice, Bread atta, suji, dhaliya, corn flakes, Poha |  |  |  |  |
| Potato, carrot, sweet potato |  |  |  |  |
| **Commercial products** - cerelac, farex |  |  |  |  |
| **Commercial infant formula-** lactogen, nestogen, Nan |  |  |  |  |
| **Pulses- nuts, legumes**,  Any dhal, lentils, Peanuts, Almonds, Cashew nuts , besan, Beans |  |  |  |  |
| **Dairy products:** milk, curds, paneer |  |  |  |  |
| Green leafy vegetables-palak, methi. |  |  |  |  |
| **Other Vegetable and Fruits:** pumpkin, bottle guard, cabbage |  |  |  |  |
| Fruits - banana, apple, papaya, orange |  |  |  |  |
| **Meat/fish/chicken** |  |  |  |  |
| **Egg** |  |  |  |  |
| **Oil/ghee/butter** |  |  |  |  |
| Miscellaneous: Sugar/ jaggary |  |  |  |  |
| Biscutes |  |  |  |  |
| sawary snacks |  |  |  |  |
| carbonated drinks |  |  |  |  |
| Tea |  |  |  |  |
| Tetra packet juices(Fruity or maza) |  |  |  |  |
| Maggi noodles, macoroni, pasta |  |  |  |  |
| junk food kurrkurr, chips, pizza,burger |  |  |  |  |
| Chocolates or sweets |  |  |  |  |

1. Are you feeding tea/ sugar drinks to your child? 1.Yes 2. no
2. Is your child receiving any snacks in between meals? 1.Yes 2. No
3. Do you wash the child’s hands with soap before feeding? 1.Yes 2.No
4. Do you wash your hands with soap and water before feeding the child? 1.Yes 2. No
5. Do you maintain eye contact and talk to your child while feeding him/her? 1.Yes 2.No
6. Do you encourage your child to take feed by praising him/her?1.Yes 2. No

|  | Item | Quantity | Calories |
| --- | --- | --- | --- |
| Early morning |  |  |  |
| Breakfast |  |  |  |
| Midmorning |  |  |  |
| Lunch |  |  |  |
| Post lunch |  |  |  |
| Evening |  |  |  |
| Dinner |  |  |  |

1. Total Calories Consumed-……………. (1=Deficit 2= Excess)……………
2. CS-ICFI score……………….

**ANNEXURE -3**

**QUESTIONNAIRE FOR HEALTH WORKERS**

**Department of Community Medicine, PGIMER**

**Title: Effectiveness of a culturally appropriate nutrition educational intervention delivered through health services to improve growth and complementary feeding of 6 months to 1 year old infants in Chandigarh: a quasi experimental study.**

1. **Basic information of ANM:**
2. ID No.
3. Name - ……………………….
4. Age - ………………………………
5. Sex – 1= M, 2= F
6. Address - ……………………………………………………..
7. Phone no. ……………………………….
8. Religion………1. Hindu 2. Muslim 3. Sikh 4.Christian 5.Others
9. Education- ………… (lliterate-1, primary school certificate- 2, middle school certificate - 3, High school certificate - 4, Intermediate or post high school diploma-5, Graduate or post graduate - 6, Professional or Honours- 7)
10. Employment status - 1.Regular 2.contract.
11. Years of service as ANM: 1. 1-3years 2. 4-6 years 3. 7-9 years 4. 10 years or more
12. **Knowledge of ANMs regarding IYCF practices**
13. When will breastfeeding be initiated after birth?

1.Immediately,within 1 hour 2.>1 hour-4 hours 3.5-24 hours 4.After 24 hours.

1. Mother’s first milk (colostrum) should be given to newborn baby?

1.Yes 2.No

1. Do you know what is exclusive breast feeding? (Choose one option)

a. Breast milk only b. Breast milk and water c. Breast milk and/or ORS/Vitamin supplements/Medicines)

(1.Yes 2. No)

1. Can bottle feeding be given to the child? 1.Yes 2. No 3. Don’t know
2. At what age complementary foods (solid, semisolid or soft foods) should be introduced? ………………………………… {1.correct 2. Incorrect 3. Don’t know}
3. Name three complementary foods that you think are best for an infant 6-8 months old? 1.……………………2……………………3…………………(1.Know 2.Don’t Know)
4. How many times a day solid/semi-solid foods should be given to 6-8 month old infant? 1. Once a day 2. 2-3 times 3. 4-5 times 4.Don’t know
5. What should be the consistency of complementary foods given to the infant? 1.Thick 2.Thin 3.Very thin
6. Name three snacks that can be given to a 6-8 month old infant? 1…………..………2………….………3…………………(1.correct 2. incorrect)
7. What is the quantity of solid/semi-solid foods a 6-8month infant can consume at his/her age per feed?
8. Start with 1-2 teaspoons and increase to 1/4^th^ of a 250 ml cup
9. Start with 2-3 tablespoons and increase to 1/2 of a 250 ml cup
10. Start with 4-5 tablespoons and increase to 3/4^th^of a 250 ml cup
11. Full cup
12. Can tea / sugar drinks can be given to the infant? 1.Yes 2. No
13. What is the minimum no. of food groups that should be given to a 6-8 month infant?

(1) Grains, roots and tubers, legumes and nuts;

(2) dairy products ;

(3) flesh foods (meat, fish, poultry);

(4) eggs,

(5) vitamin A rich fruits and vegetables;

(6) other fruits and vegetables

a. 2-3 food groups b. 4 or more food groups (1. Know 2. Don’t know)

1. Do you know till what age breastfeeding can be continued? …………………………………. (1. Know 2.Don’t know)
2. Can ghee/oil/butter be added to the food of infants? 1.Yes 2. No
3. Do you have knowledge about responsive feeding? 1.Yes 2.No
4. **PRACTICE OF ANM REGARDING IYCF PRACTICES**
5. Do you advice mothers to wash their hands with soap and water before feeding infant? 1.Yes 2. No
6. Do you advice mother to wash the infant’s hands before feeding? 1.Yes 2. No
7. Do you advice mother to maintain eye contact and talk to infant while feeding him/her? 1.Yes 2. No
8. Do you tell the mothers to add ghee/oil/butter to the food of infants? 1.Yes 2. No
9. Do you tell the mothers to feed snacks to the infants in between meals? 1.Yes 2. No

| Does this practice occur? | 1.With all children | 2.With some children | 3.Does not occur | 4.Others (comment) |
| --- | --- | --- | --- | --- |
| 31.Weigh child during routine immunization |  |  |  |  |
| 32. Measure Child Growth during routine immunization |  |  |  |  |
| 33. Discuss how the child is feeding during routine immunization |  |  |  |  |
| 34. Group counseling on IYCF during routine immunization sessions |  |  |  |  |
| 35. Carry out demonstrations of young children’s food preparations and feeding techniques during VHND |  |  |  |  |
| 36. Make home visits to assess foods and feeding practices |  |  |  |  |

**ANNEXURE 4**

**TRAINING MODULES FOR HEALTH WORKERS**

**Study Title: Effectiveness of a culturally appropriate nutrition educational intervention delivered through health services to improve growth and complementary feeding of 6 months to 1 year old infants in Chandigarh: a quasi experimental study.**

Name of institute: Post graduate institute of medical research and training (PGIMER)

***Session 1*** –Introduction & orientation

*1. Importance of complementary feeding*

In this session we will:

- define the term complementary feeding;
- discuss the optimal age for children to start complementary feeding;
- discuss the importance of continuing breastfeeding;
- examine the role of health worker and health facility; and
- list the key messages to discuss with caregivers about when to start complementary foods.

Key Messages:

1. Breastfeeding for two years of age or longer helps a child to develop and grow strong and healthy.
2. Starting other foods in addition to breast milk at six months helps a child to grow well.

Most babies do not need complementary foods before six months of age. All babies older than six months of age should receive complementary foods.

*2. Foods to fill the energy gap*

In this session we will:

- discuss the local foods that can help fill this energy gap;

- - - Andexplain the importance of using foods of a thick consistency;
    - discuss ways to enrich foods; and
    - list the key message of how to fill this gap to discuss with caregivers. Key Message:
  1. Foods that are thick enough to stay in the spoon give more energy to the child.

1. *Foods to fill the iron and vitamin A gaps*

In this session we will:

- - - discuss the local foods that can fill the nutrient gaps for iron and vitamin A;
    - discuss the use of processed complementary foods;
    - discuss the fluid needs of the young child;
    - list the key messages of how to fill these gaps to discuss with caregivers

Key Messages:

- 1. Animal foods are speciallygood for children, to help them grow strong and lively.
  2. Peas, beans, lentils, and nuts and seeds are good for children.
  3. Dark green leaves and orange coloured fruit and vegetables help the child to have healthy eyes and fewer infections.

1. *Quantity, variety and frequency of feeding*

In this session we will discuss:

- how to use a mixture or variety of foods to help fill the gaps;
- how often to feed complementary foods;
- the quantity of complementary foods to offer, and
- the key messages to share with caregivers and your co- workers.

Key Messages:

1. A growing child needs three meals plus snacks: give a variety of foods.
2. A growing child needs increasing amounts of food.

| Age | Texture | Frequency | Amount at each meal |
| --- | --- | --- | --- |
| from 6 months | Soft porridge, well  Mashed vegetable, meat, fruit | two times per day plus  frequent breastfeeds | 2 to 3 tablespoonfuls |
| 7 to 8 months | Mashed foods | Three times per day plus frequent breastfeeds | increasing gradually to  2/3 of a 250 ml cup at  each meal |
| 9 to11 months | Finely chopped or  Mashed foods, and foods that baby canpick up | three meals plus one  snack between meals  plus breastfeeds | 3/4 of a 250 ml  cup/bowl |
| 12 to 24 months | Family foods, chopped or mashed if necessary | three meals plus two  snacks between meals  plus breastfeeds | A full 250 ml cup/bowl |

1. *Feeding During Illness and Recovery*

In this session we will look at:

- - - the importance of continuing to feed a child during illness;
  - ways of encouraging children to eat during illness and recovery; and counseling caregivers on appropriate feeding practices during illness.

Key Messages:

1. Encourage the child to drink and to eat during illness and provide extra food after illness to help them recover quickly.

***Session 2-*** Communication Skills:

***1.*** *Listening and learning skills*

In this session we will discuss:

- How to use basic counseling skills to listen and learn from caregivers about their complementary practices. For example:

- 1. Use helpful non-verbal communication
  2. Ask open questions
  3. Use responses and gestures that show interest
  4. Reflect back what the caregiver says
  5. Empathize – show that you understand how she/he feels
  6. Avoid words that sound judging

1. *Building confidence skills*

In this session we will discuss:

- How to use basic counseling skills to build confidence and give support to caregivers about their complementary feeding practices.

1. Accept what a caregiver thinks and feels
2. Recognize and praise what a caregiver and child are doing right
3. Give practical help
4. Give a little relevant information
5. Use simple language
6. Make one or two suggestions, not commands

***Session 3-*** Home visit tasks and procedures:

***1.*** *Gathering Information on Complementary Feeding Practices*

In this session we will look at:

- - the importance of observation skills and observing interactions between caregivers and children;
  - using growth charts in assessment of complementary feeding practices;
  - how to gather information on complementary feeding practices using a Food Intake Tool.

1. *Feeding Techniques and Strategies*

In this session we will look at:

- - feeding practices and their effect on the child’s intake;
  - ways of encouraging responsive feeding practices;
- requirements for clean and safe feeding of young children; and
- how possible it is to carry out these recommendations.

f*ood Intakes and Counseling at each visit*

| Sr. No. | Caregivers practices | Practice in place | Informed/ Suggested |
| --- | --- | --- | --- |
| 1 | Child receives breast milk? |  |  |
| 2 | Child eats three meals of thick consistency yesterday? |  |  |
| 3 | Child ate an animal product yesterday (meat/fish/bird/eggs)? |  |  |
| 4 | Child ate a dairy product yesterday? |  |  |
| 5 | Child ate pulses or nuts yesterday? |  |  |
| 6 | Child ate a dark green or orange vegetable or orange fruit yesterday? |  |  |
| 7 | Child eats sufficient number of meals and snacks yesterday, for his/her age? |  |  |
| 8 | Quantity of food eaten at main meal yesterday appropriate for child’s age? |  |  |
| 9 | Caregiver assists the child at meals times? |  |  |
| 10 | Child takes any vitamin or mineral supplements? |  |  |
| 11 | Child ill and not eating? |  |  |

**ANNEXURE- 5**

**TRAINING MODULE FOR MOTHER**

**Study Title: Effectiveness of a culturally appropriate nutrition educational intervention delivered through health services to improve growth and complementary feeding of 6 months to 1 year old infants in Chandigarh: a quasi experimental study.**

Name of institute: Post graduate institute of medical research and training (PGIMER)

1. KEY MESSAGES FOR COMPLEMENTARY FEEDING
   1. Breastfeeding for two years of age or longer helps a child to develop and grow strong and healthy.
   2. Starting other foods in addition to breast milk at six months helps a child to grow well.
   3. Foods that are thick enough to stay in the spoon give more energy to the child.
   4. Animal foods are especially good for children, to help them grow strong and lively.
   5. Peas, beans, lentils, and nuts and seeds, are good for children.
   6. Dark green leaves and orange colored fruits and vegetables help the child to have healthy eyes and fewer infections.
   7. A growing child needs three meals and snacks: give a variety of foods.
   8. A growing child needs increasing amounts of food.
   9. A young child needs to learn to eat: encourage and give help…with lots of patience.
   10. Encourage the child to drink and to eat during illness and provide extra food after illness to help them recover quickly.

B.AMOUNTS OF FOODS TO OFFER

| Age | Texture | Frequency | Amount at each meal |
| --- | --- | --- | --- |
| from 6 months | Soft porridge, well  Mashed vegetable, meat, fruit | two times per day plus  frequent breastfeeds | 2 to 3 tablespoonfuls |
| 7 to 8 months | Mashed foods | Three times per day plus frequent breastfeeds | increasing gradually to  2/3 of a 250 ml cup at  each meal |
| 9 to11 months | Finely chopped or  Mashed foods, and foods that baby canpick up | three meals plus one  snack between meals  plus breastfeeds | 3/4 of a 250 ml  cup/bowl |
| 12 to 24 months | Family foods, chopped or mashed if necessary | three meals plus two  snacks between meals  plus breastfeeds | A full 250 ml cup/bowl |

C. FIVE WAYS TO KEEP SAFE FOODS

1. Keep the hands clean

Wash your hands before handling food and often during food preparation.

Wash your hands after going to the toilet, changing the baby or in contact with animals. Wash very clean all surfaces and equipment used for food preparation or serving. Protect kitchen areas and food from insects, pests and other animals.

2. Separately keep the raw foods and cooked foods

Use separate equipment and utensils for handling raw meat and foods.

Store foods in separate covered containers.

3. Cook thoroughly

Cook food thoroughly, especially meat, poultry and eggs. Bring foods like soups and stews to boiling point.

For meat and poultry, make sure juices are clear not pink.

Reheat cooked food thoroughly. Bring to the boil or heat until too hot to touch. Stir while re-heating.

4. Keep cooked food at even and safe temperatures

Do not leave cooked food at room temperature for more than two hours. Do not store food too long, even in a refrigerator.

Do not thaw frozen food at room temperature.

Food for infants and young children should ideally be freshly prepared and not stored at all after cooking.

5. Use safe water and raw materials

Use safe water or treat it to make it safe. Choose fresh and wholesome foods.

Use pasteurized milk.

Wash fruits and vegetables in safe water, especially if eaten raw.

**ANNEXURE-6**

**INFORMED CONSENT FORM**

**Study Title: Effectiveness of a culturally appropriate nutrition educational intervention delivered through health services to improve growth and complementary feeding of 6 months to 1 year old infants in Chandigarh: a quasi experimental study.**

Subject’s Initials: _______________

Subject’s Name:_______________

Date of Birth / Age: _________________

|  | Participant's initial |
| --- | --- |
| 1. I confirm that I have read and understood the information sheet dated _ _ _ for the above study and have had the opportunity to ask questions. |  |
| 2. I understand that my participation in the study is voluntary and that I am free to withdraw at any time, without giving any reason, without my medical care or legal rights being affected |  |
| 3. I understand that the Sponsor of the clinical trial, others working on the Sponsor’s behalf, the Ethics Committee and the regulatory authorities will not need my permission to look at my health records both in respect of the current study and any further research that may be conducted in relation to it, even if I withdraw from the trial. I agree to this access. However, I understand that my identity will not be revealed in any information released to third parties or published |  |
| 4. I agree not to restrict the use of any data or results that arise from this study provided such a use is only for scientific purpose(s). |  |
| 5. I agree to take part in the above study. |  |

Signature (or Thumb impression) of the Subject:_____________

Date: _____/_____/______

Signatory’s Name: ________________________________________

Signature of the Investigator: ____________________________

Date:_____/_____/______

Study Investigator’s Name: ____________________________________

**ANNEXURE 7**

**PARTICIPANT INFORMATION SHEET**

**Study Title: Effectiveness of a culturally appropriate nutrition educational intervention delivered through health services to improve growth and complementary feeding of 6 months to 1 year old infants in Chandigarh: a quasi experimental study.**

Dear Parent/Guardian/Participant,

We would like to invite you and/or your child to participate in the research study. You are free to choose to either participate or withdraw from the study.

**Name of the participants**

Mother/Caregiver/Participant …………………..…

Child …………………………

**Purpose of the study**

This study is being done to to assess the effectiveness of culturally appropriate nutrition educational intervention delivered through health services to improve growth and complementary feeding by infants of age 6 months to 1 year old in Chandigarh.

We will ask you some basic questions regarding you and your child and household.

Please take the time to read or to listen as I read the following information. The information in this document is meant to help you decide whether or not to take part in this study. You may talk to others about the study if you wish. Participation in this study is completely voluntary. You may withdraw from this study at any time. Please ask me if there is anything that is not clear, or if you would like more information. When all of your questions have been answered and you understand the study, we will ask for your permission to allow you to take part in the study and to sign this consent form.

**Procedures that will be followed during this study**

If you agree to take part in the study, you will complete an interview with our interviewer who will ask you basic questions. The interview will take some of your valuable time. During this interview, some basic questions will be asked regarding you and your child and household. A routine physical and anthropometric measurement will be done after the interview.

**Benefits and Risks**

It may help to improve the feeding habits of your child and improve his nutritional status. No possible risk.

**Compensation**

We would not be able to provide any compensation to anyone who participates in thisstudy.

**Confidentiality**

Your participation will be kept confidential. Your name will not be revealed to anyone outside of the study team. Your answers will be treated with confidentiality and will be revealed only to the doctors/researchers involved in this study. The result of this study may be published in a report/scientific journal but your name will not be identified.

**Voluntary participation/ withdrawal and right to refuse or withdraw from the study**

Your participation in the research study is completely voluntary. This means that you can choose not to take part in this study. If you agree to be interviewed, you can refuse to respond to any question. You can also stop the interview at any time.

**Questions**

We have used some technical terms in this form. Please feel free to ask about anything you don’t understand and to consider this research and the consent form carefully before you make a decision.

**Cost to the participant**

You will not be paid to participate in the study.

**Right to new information**

If the research team gets any new information during the research study that may affect your decision to continue participating in the study, or may raise some doubts, you will be told about that information.

**Contact information**

For further information/questions, you may contact us at the following address:

1. Dr. Nikita Sharma, Department of Community Medicine.
   Phone no.- 8968874332
2. Dr. Madhu Gupta, Additional Professor, Department of Community Medicine

In case of conflicts, you can contact the chairperson of our institutional ethics committee at the following address:

Chairperson, Institutional Ethics Committee

PGIMER, Chandigarh

In case of conflicts, you can contact the chairperson (convener) of our institutional ethics committee at the following address:

Convener/Chairperson, Institutional Ethics Committee

PGIMER, Chandigarh

Telephone: ................

**References**

[1] UNICEF. Levels and trends in child mortality: Report 2014. 2014.

[2] Victora CG, de Onis M, Hallal PC, Blössner M SR. Worldwide Timing of Growth Faltering : Revisiting Implications for Interventions. Pediatrics 2010;125:e473-480. doi:10.1542/peds.2009-1519.

[3] Victora CG, Adair L, Fall C, Hallal PC, Martorell R, Richter L, et al. Maternal and Child Undernutrition 2 Maternal and child undernutrition : consequences for adult health and human capital 2008;371:340–57. doi:10.1016/S0140-6736(07)61692-4.

[4] World Bank. 2012. India - Nutrition at a glance. Nutrition at a glance ; India. Washington, DC: 2012.

[5] Indian Council of Medical research. Nutrient requirements and recommended dietary allowances for Indians A Report of the Expert Group of the Indian Council of Medical Research. 2009.

[6] UNICEF. The State of the world’s children 2009. 2009.

[7] International Institute for Population Sciences. India National Family Health Survey (NFHS-3), 2005-06. vol. 1. International Institute for Population Sciences; 2007.

[8] Lokshin M, Gupta M Das, Gragnolati M, Ivaschenko O. Improving Child Nutrition ? The Integrated Child Development Services in India. Wiley-Blakewell 2005;36:613–40.

[9] World health organization. Student’s Handbook IMNCI integrated management of neonatal and childhood Illnesses. Geneva WHO 2003:13–8.

[10] Mohan P, Kishore B, Singh S, Bahl R, Puri A, Kumar R. Assessment of Implementation of Integrated Management of Neonatal and Childhood Illness in India. J Heal Popul Nutr 2011;29:629–38.

[11] Srivastava A, Gope R, Nair N, Rath S, Sinha R SP. Are village health sanitation and nutrition committees fulfilling their roles for decentralised health planning and action ? A mixed method study from rural eastern India. BMC Public Health 2016;16:59. doi:10.1186/s12889-016-2699-4.

[12] Chaturvedi A, Nakkeeran N, Doshi M, Patel R BS. Capacity of frontline ICDS functionaries to support caregivers on infant and young child feeding ( IYCF ) practices in Gujarat , India. Asia Pac J Clin Nutr 2014;23:S29-37. doi:10.6133/apjcn.2014.23.s1.04.

[13] Imdad A, Yakoob MY, Bhutta ZA. Impact of maternal education about complementary feeding and provision of complementary foods on child growth in developing countries. BMC Public Health 2011;11:S25. doi:10.1186/1471-2458-11-S3-S25.

[14] Nutrition: Complementary feeding. World Heal Organ 2017. http://www.who.int/nutrition/topics/complementary_feeding/en/ (accessed 27 November 2017).

[15] Brown KH, Duggan C, Watkins JB WW. Complementary Feeding. In: Duggan C, Watkins JB WA, editor. Nutr. Pediatr. Basic Sci. Clin. Appl. 4th ed., B.C. Decker; 2008, p. 411–6.

[16] Duijts L, Jaddoe VWV, Hofman A MH. Prolonged and Exclusive Breastfeeding Reduces the Risk of Infectious Diseases in Infancy. Pediatrics 2010;126:e18-25. doi:10.1542/peds.2008-3256.

[17] Report of Informal Meeting to Review and Develop Indicators for Complementary Feeding. Washington D.C.: 2002.

[18] Bhutta ZA, Ahmed T, Black RE, Cousens S, Dewey K, Giugliani E, et al. What works? Interventions for maternal and child undernutrition and survival. Lancet 2008;371:417–40.

[19] Penny ME, Creed-Kanashiro HM, Robert RC, Narro MR, Caulfield LE, Black RE. Effectiveness of an educational intervention delivered through the health services to improve nutrition in young children: a cluster-randomised controlled trial. Lancet 2005;365:1863–72.

[20] Bhandari N, Mazumder S, Bahl R, Martines J, Black RE, Bhan MK, et al. An educational intervention to promote appropriate complementary feeding practices and physical growth in infants and young children in rural Haryana, India. J Nutr 2004;134:2342–8.

[21] Guldan GS, Fan H-C, Ma X, Ni Z-Z, Xiang X, Tang M-Z. Culturally appropriate nutrition education improves infant feeding and growth in rural Sichuan, China. J Nutr 2000;130:1204–11.

[22] Roy SK, Jolly SP, Shafique S, Fuchs GJ, Mahmud Z, Chakraborty B, et al. Prevention of malnutrition among young children in rural Bangladesh by a food-health-care educational intervention: a randomized, controlled trial. Food Nutr Bull 2007;28:375–83.

[23] UNICEF - Progress for Children - What is undernutrition? UnicefOrg 2017. http://www.unicef.org/progressforchildren/2006n4/index_undernutrition.html. (accessed 27 November 2017).

[24] Pelletier J-G. Severe Malnutrition: A Global Approach. Child Trop 1993;208:209.

[25] Causes and most vulnerable to undernutrition - UNICEF Conceptual Framework. UnicefOrg 2008. https://www.unicef.org/nutrition/training/2.5/4.html (accessed 27 November 2017).

[26] Shah PM. Early detection and prevention of protein calorie malnutrition. Bombay: Popular Prakashan; 1974.

[27] Shi L, Zhang J. Recent evidence of the effectiveness of educational interventions for improving complementary feeding practices in developing countries. J Trop Pediatr 2011;57:91–8.

[28] Dewey KG, Brown KH. Update on technical issues concerning complementary feeding of young children in developing countries and implications for intervention programs. Food Nutr Bull 2003;24:5–28.

[29] Vorster HH, Kruger A. Poverty, malnutrition, underdevelopment and cardiovascular disease: a South African perspective. Cardiovasc J Afr 2007;18:321–4.

[30] Mohiddin L, Phelps L, Walters T. Urban malnutrition: a review of food security and nutrition among the urban poor. Nutr Work 2012;8.

[31] World health organization. WHO Global database on child growth and malnutrition. Child malnutrition estimates by WHO Child Growth Standards based on WHO child growth standards UNITED STATES OF AMERICA (THE). 2014.

[32] Fitchen JM. Hunger, malnutrition, and poverty in the contemporary United States: Some observations on their social and cultural context. Food Foodways 1987;2:309–33.

[33] Black RE, Victora CG, Walker SP, Bhutta ZA, Christian P, De Onis M, et al. Maternal and child undernutrition and overweight in low-income and middle-income countries. Lancet 2013;382:427–51.

[34] UNICEF. Committing to Child Survival: A Promise Renewed. 2015.

[35] State Fact Sheet Chandigarh District Level Household and Facility Survey-4 Ministry of Health and Family Welfare. 2012.

[36] Thakur JS, Prinja S, Bhatia SS. Persisting malnutrition in Chandigarh: decadal underweight trends and impact of ICDS program. Indian Pediatr 2011;48:315–8.

[37] Britton C, McCormick FM, Renfrew MJ, Wade A, King SE. Support for breastfeeding mothers. Cochrane Database Syst Rev 2007;1:CD001141.

[38] Pachón H, Schroeder DG, Marsh DR, Dearden KA, Ha TT, Lang TT. Effect of an integrated child nutrition intervention on the complementary food intake of young children in rural north Viet Nam. Food Nutr Bull 2002;23:59–66.

[39] Ashworth A, Chopra M, McCoy D, Sanders D, Jackson D, Karaolis N, et al. WHO guidelines for management of severe malnutrition in rural South African hospitals: effect on case fatality and the influence of operational factors. Lancet 2004;363:1110–5.

[40] Humphrey JH, Agoestina T, Wu L, Usman A, Nurachim M, Subardja D, et al. Impact of neonatal vitamin A supplementation on infant morbidity and mortality. J Pediatr 1996;128:489–96.

[41] Bhandari N, Bahl R, Taneja S, Strand T, Mølbak K, Ulvik RJ, et al. Substantial reduction in severe diarrheal morbidity by daily zinc supplementation in young north Indian children. Pediatrics 2002;109:e86–e86.

[42] Behrman J, Hoddinott J. An evaluation of the impact of PROGRESA on pre-school child height. Int Food Policy Res Institute, Washington, DC 2000.

[43] Maluccio J, Flores R. Impact evaluation of a conditional cash transfer program: The Nicaraguan Red de Protección Social. Intl Food Policy Res Inst; 2005.

[44] Gibson RS, Yeudall F, Drost N, Mtitimuni BM, Cullinan TR. Experiences of a community-based dietary intervention to enhance micronutrient adequacy of diets low in animal source foods and high in phytate: a case study in rural Malawian children. J Nutr 2003;133:3992S-3999S.

[45] Yeudall F, Gibson RS, Cullinan TR, Mtimuni B. Efficacy of a community-based dietary intervention to enhance micronutrient adequacy of high-phytate maize-based diets of rural Malawian children. Public Health Nutr 2005;8:826–36.

[46] Cambridge. Culture meaning in the Cambridge English dictionary. © Cambridge Univ Press 2016. http://dictionary.cambridge.org/dictionary/english/culture.

[47] UNICEF. Complementary feeding of young children in developing countries: a review of current scientific knowledge. WHO; 1998.

[48] Allen LH, Gillespie SR. What works? A review of the efficacy and effectiveness of nutrition interventions. Asian Development Bank; 2001.

[49] Caulfield LE, Huffman SL, Piwoz EG. Interventions to improve intake of complementary foods by infants 6 to 12 months of age in developing countries: impact on growth and on the prevalence of malnutrition and potential contribution to child survival. Food Nutr Bull 1999;20:183–200.

[50] Schroeder DG, Martorell R, Rivera JA, Ruel MT, Habicht J-P. Age Differences in the Impact of Nutritional Supplementation. J Nutr 1995;125:1051S.

[51] Zaman S, Ashraf RN, Martines J. Training in complementary feeding counselling of healthcare workers and its influence on maternal behaviours and child growth: a cluster-randomized controlled trial in Lahore, Pakistan. J Health Popul Nutr 2008;26:210.

[52] Zahid Khan A, Rafique G, Qureshi H, Halai Badruddin S. A nutrition education intervention to combat undernutrition: experience from a developing country. ISRN Nutr 2013;2013.

[53] Kushwaha KP, Sankar J, Sankar MJ, Gupta A, Dadhich JP, Gupta YP, et al. Effect of peer counselling by mother support groups on infant and young child feeding practices: the Lalitpur experience. PLoS One 2014;9:e109181.

[54] Kilaru A, Griffiths PL, Ganapathy S, Shanti G. Community-based nutrition education for improving infant growth in rural Karnataka. Indian Pediatr 2005;42:425.

[55] Rao S, Swathi PM, Unnikrishnan B, Hegde A. Study of complementary feeding practices among mothers of children aged six months to two years-A study from coastal south India. Australas Med J 2011;4:252.

[56] Chandramouli C. Census of india 2011: rural urban distribution of population (Provisional population totals). Indian Ministry of Home Affairs. New Delhi. vol. 4. 2011.

[57] Chandramouli C. Census of India 2011.Provisional Population Totals.New Delhi: Government of India. 2011.

[58] Kirkwood BR, Sterne JAC. Essential medical statistics. Second edi. John Wiley & Sons; 2010.

[59] Kulwa KBM, Verstraeten R, Bouckaert KP, Mamiro PS, Kolsteren PW, Lachat C. Effectiveness of a nutrition education package in improving feeding practices, dietary adequacy and growth of infants and young children in rural Tanzania: rationale, design and methods of a cluster randomised trial. BMC Public Health 2014;14:1077.

[60] UNICEF. HEIGHT/LENGTH MEASURING BOARDS. UNICEF. Technical Bulletin No.18. 2012. doi:http://www.unicef.org/supply/files/Height_Length_Measuring_Boards.pdf.

[61] World Health Organization. Complementary feeding: report of global consultation and summary of guiding principles for complementary feeding of the breastfed child. Geneva: 2001.

[62] World Health Organization. Complementary feeding counselling: a training course. 2004.
