# Supplementary material for: Effectiveness of a culturally appropriate nutrition educational intervention delivered through health services to improve growth and complementary feeding of infants: A quasi-experimental study from Chandigarh, India
Source: PLoS One. 2020 Mar 17;15(3):e0229755. doi: 10.1371/journal.pone.0229755 (PMC7077818; doi:10.1371/journal.pone.0229755)

**S2 File. TREND Statement Checklist**

|  | **Paper** |  |  | **Item** |  | **Descriptor** | **Reported?** | | |  |  |
| --- | --- | --- | --- | --- | --- | --- | --- | --- | --- | --- | --- |
|  | **Section/** |  |  | **No** |  |  |  |  | **Pg #** |  |  |
|  | **Topic** |  |  |  |  |  |  |  |  |  |  |
|  | **Title and Abstract** | | | |  |  |  |  |  |  |  |
|  | Title and |  | 1 | |  | Information on how unit were allocated to interventions | ✔ |  | 4 |  |  |
|  | Abstract |  |  |  |  | Structured abstract recommended | ✔ |  | 4 |  |  |
|  |  |  |  |  |  | Information on target population or study sample | ✔ |  | 4 |  |  |
|  | **Introduction** |  |  |  |  |  |  |  |  |  |  |
|  | Background |  | 2 | |  | Scientific background and explanation of rationale | ✔ |  | 6-7 |  |  |
|  |  |  |  |  |  | Theories used in designing behavioral interventions | 🗙 |  |  |  |  |
|  | **Methods** |  |  |  |  |  |  |  |  |  |  |
|  | Participants |  | 3 | |  | Eligibility criteria for participants, including criteria at different levels in | ✔ |  | 10 |  |  |
|  |  |  |  |  |  | recruitment/sampling plan (e.g., cities, clinics, subjects) |  |  |  |  |  |
|  |  |  |  |  |  | Method of recruitment (e.g., referral, self-selection), including the | ✔ |  | 11 |  |  |
|  |  |  |  |  |  | sampling method if a systematic sampling plan was implemented |  |  |  |  |  |
|  |  |  |  |  |  | Recruitment setting | ✔ |  | 11 |  |  |
|  |  |  |  |  |  | Settings and locations where the data were collected | ✔ |  | 8-9 |  |  |
|  | Interventions |  | 4 | |  | Details of the interventions intended for each study condition and how |  |  |  |  |  |
|  |  |  |  |  |  | and when they were actually administered, specifically including: |  |  |  |  |  |
|  |  |  |  |  |  | o Content: what was given? | ✔ |  | 14-15 |  |  |
|  |  |  |  |  |  | o Delivery method: how was the content given? | ✔ |  | 14-15 |  |  |
|  |  |  |  |  |  | o Unit of delivery: how were the subjects grouped during delivery? | ✔ |  | 15 |  |  |
|  |  |  |  |  |  | o Deliverer: who delivered the intervention? | ✔ |  | 14 |  |  |
|  |  |  |  |  |  | o Setting: where was the intervention delivered? | ✔ |  | 13 |  |  |
|  |  |  |  |  |  | o Exposure quantity and duration: how many sessions or episodes or | ✔ |  | 14 |  |  |
|  |  |  |  |  |  | events were intended to be delivered? How long were they |  |  |  |  |  |
|  |  |  |  |  |  | intended to last? |  |  |  |  |  |
|  |  |  |  |  |  | o Time span: how long was it intended to take to deliver the | ✔ |  | 16 |  |  |
|  |  |  |  |  |  | intervention to each unit? |  |  |  |  |  |
|  |  |  |  |  |  | o Activities to increase compliance or adherence (e.g., incentives) | ✔ |  | 14 |  |  |
|  | Objectives |  | 5 | |  | Specific objectives and hypotheses | ✔ |  | 7 |  |  |
|  | Outcomes |  | 6 | |  | Clearly defined primary and secondary outcome measures | ✔ |  | 17 |  |  |
|  |  |  |  |  |  | Methods used to collect data and any methods used to enhance the | ✔ |  | 17 |  |  |
|  |  |  |  |  |  | quality of measurements |  |  |  |  |  |
|  |  |  |  |  |  | Information on validated instruments such as psychometric and biometric | ✔ |  | 12-13 |  |  |
|  |  |  |  |  |  | properties |  |  |  |  |  |
|  | Sample Size |  | 7 | |  | How sample size was determined and, when applicable, explanation of any | ✔ |  | 10 |  |  |
|  |  |  |  |  |  | interim analyses and stopping rules |  |  |  |  |  |
|  | Assignment |  | 8 | |  | Unit of assignment (the unit being assigned to study condition, e.g., | ✔ |  | 8 |  |  |
|  | Method |  |  |  |  | individual, group, community) |  |  |  |  |  |
|  |  |  |  |  |  | Method used to assign units to study conditions, including details of any | ✔ |  | 8 |  |  |
|  |  |  |  |  |  | restriction (e.g., blocking, stratification, minimization) |  |  |  |  |  |
|  |  |  |  |  |  | Inclusion of aspects employed to help minimize potential bias induced due | ✔ |  | 8 |  |  |
|  |  |  |  |  |  | to non-randomization (e.g., matching) |  |  |  |  |  |


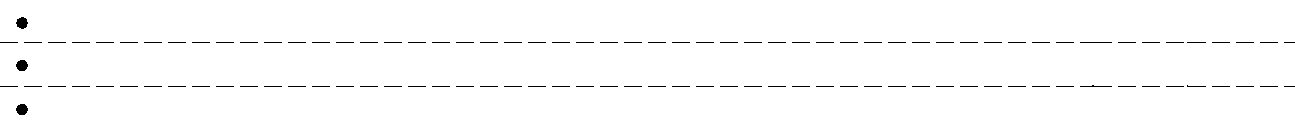

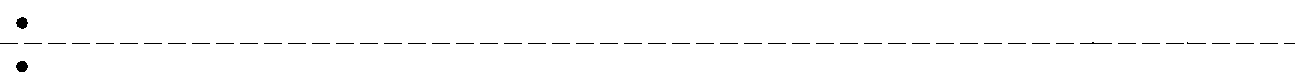

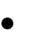

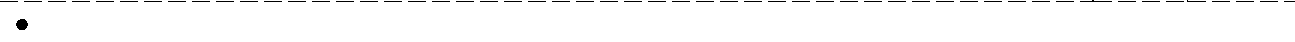

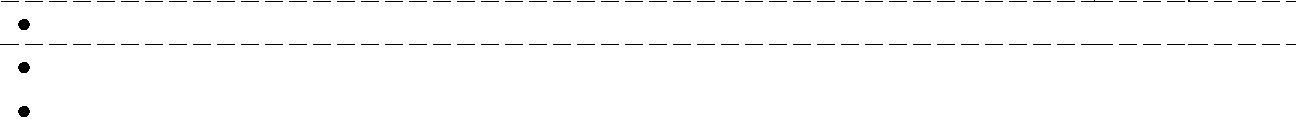

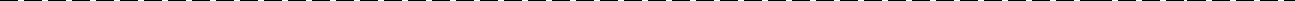

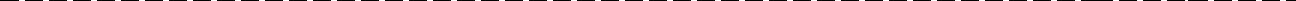

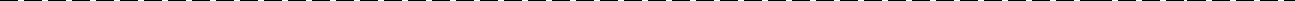

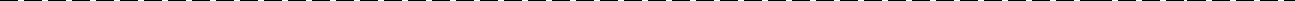

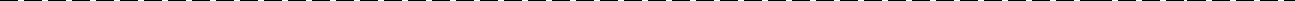

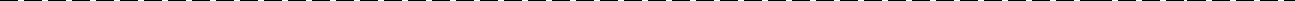

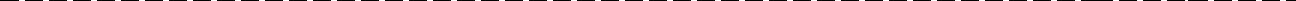

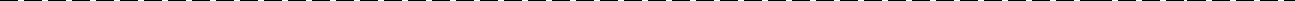

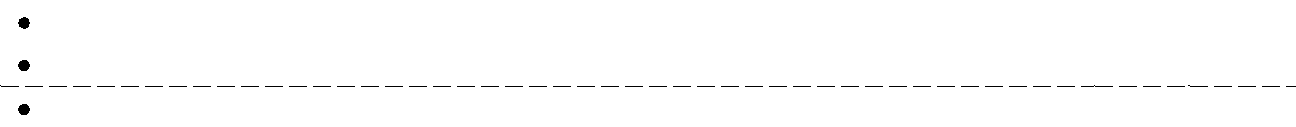

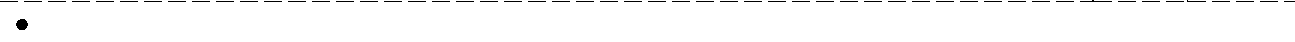

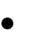

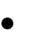

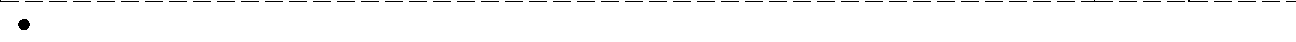

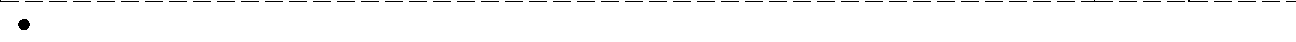

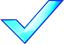


**TREND Statement Checklist**

| Blinding | 9 | Whether or not participants, those administering the interventions, and | ✔ | 37 |
| --- | --- | --- | --- | --- |
| (masking) |  | those assessing the outcomes were blinded to study condition assignment; |  |  |
|  |  | if so, statement regarding how the blinding was accomplished and how it |  |  |
|  |  | was assessed. |  |  |
|  |  |  |  |  |
| Unit of Analysis | 10 | Description of the smallest unit that is being analyzed to assess | ✔ | 17 |
|  |  | intervention effects (e.g., individual, group, or community) |  |  |
|  |  | If the unit of analysis differs from the unit of assignment, the analytical | 🗙 |  |
|  |  | method used to account for this (e.g., adjusting the standard error |  |  |
|  |  | estimates by the design effect or using multilevel analysis) |  |  |
| Statistical | 11 | Statistical methods used to compare study groups for primary methods | ✔ | 17 |
| Methods |  | outcome(s), including complex methods of correlated data |  |  |
|  |  | Statistical methods used for additional analyses, such as a subgroup | 🗙 |  |
|  |  | analyses and adjusted analysis |  |  |
|  |  | Methods for imputing missing data, if used | 🗙 |  |
|  |  | Statistical software or programs used | ✔ | 17 |
| **Results** |  |  |  |  |
| Participant flow | 12 | Flow of participants through each stage of the study: enrollment, | ✔ | 10 |
|  |  | assignment, allocation, and intervention exposure, follow-up, analysis (a |  |  |
|  |  | diagram is strongly recommended) |  |  |
|  |  | o Enrollment: the numbers of participants screened for eligibility, | ✔ | 10 |
|  |  | found to be eligible or not eligible, declined to be enrolled, and |  |  |
|  |  | enrolled in the study |  |  |
|  |  | o Assignment: the numbers of participants assigned to a study | ✔ | 18 |
|  |  | condition |  |  |
|  |  | o Allocation and intervention exposure: the number of participants | ✔ | 10 |
|  |  | assigned to each study condition and the number of participants |  |  |
|  |  | who received each intervention |  |  |
|  |  | o Follow-up: the number of participants who completed the follow- | ✔ | 18 |
|  |  | up or did not complete the follow-up (i.e., lost to follow-up), by |  |  |
|  |  | study condition |  |  |
|  |  | o Analysis: the number of participants included in or excluded from | ✔ | 18 |
|  |  | the main analysis, by study condition |  |  |
|  |  | Description of protocol deviations from study as planned, along with | ✔ | 10 |
|  |  | reasons |  |  |
| Recruitment | 13 | Dates defining the periods of recruitment and follow-up | ✔ | 13 |
| Baseline Data | 14 | Baseline demographic and clinical characteristics of participants in each | ✔ | 18-20 |
|  |  | study condition |  |  |
|  |  | Baseline characteristics for each study condition relevant to specific | ✔ | 18-20 |
|  |  | disease prevention research |  |  |
|  |  | Baseline comparisons of those lost to follow-up and those retained, overall | ✔ | 18-20 |
|  |  | and by study condition |  |  |
|  |  | Comparison between study population at baseline and target population | ✔ | 18-20 |
|  |  | of interest |  |  |
| Baseline | 15 | Data on study group equivalence at baseline and statistical methods used | ✔ | 18-20 |
| equivalence |  | to control for baseline differences |  |  |
|  |  |  |  |  |


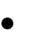

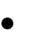

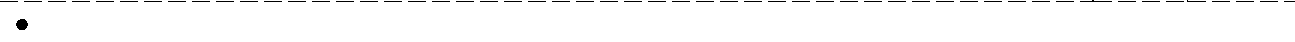

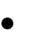

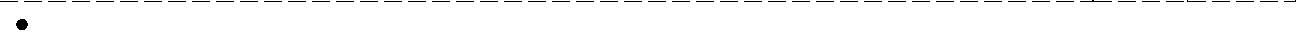

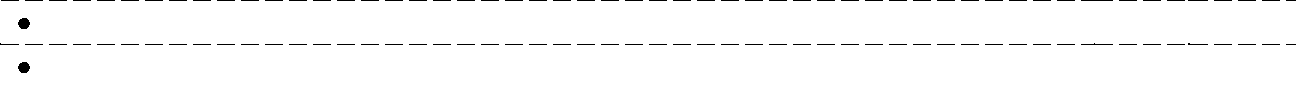

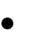

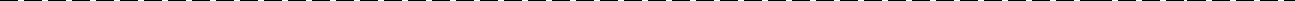

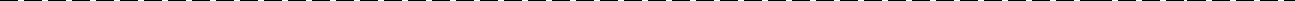

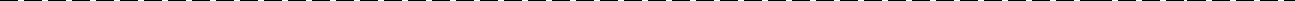

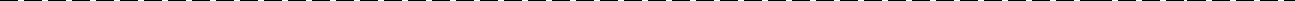

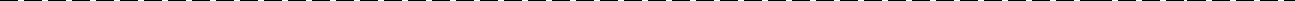

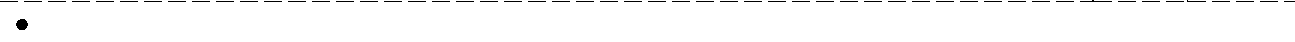

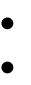

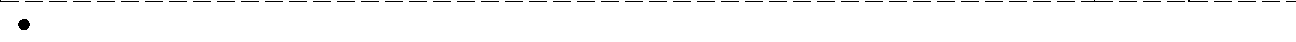

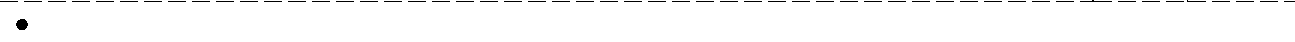

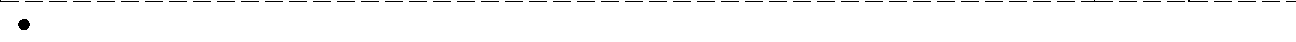

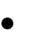


**TREND Statement Checklist**

| Numbers | 16 | Number of participants (denominator) included in each analysis for each | ✔ | 18 |
| --- | --- | --- | --- | --- |
| analyzed |  | study condition, particularly when the denominators change for different |  |  |
|  |  | outcomes; statement of the results in absolute numbers when feasible |  |  |
|  |  | Indication of whether the analysis strategy was “intention to treat” or, if | ✔ | 17 |
|  |  | not, description of how non-compliers were treated in the analyses |  |  |
| Outcomes and | 17 | For each primary and secondary outcome, a summary of results for each | ✔ | 28 |
| estimation |  | estimation study condition, and the estimated effect size and a confidence |  |  |
|  |  | interval to indicate the precision |  |  |
|  |  | Inclusion of null and negative findings | ✔ | 20-25 |
|  |  | Inclusion of results from testing pre-specified causal pathways through | 🗙 |  |
|  |  | which the intervention was intended to operate, if any |  |  |
| Ancillary | 18 | Summary of other analyses performed, including subgroup or restricted | 🗙 |  |
| analyses |  | analyses, indicating which are pre-specified or exploratory |  |  |
| Adverse events | 19 | Summary of all important adverse events or unintended effects in each | ✔ | 18 |
|  |  | study condition (including summary measures, effect size estimates, and |  |  |
|  |  | confidence intervals) |  |  |
| **DISCUSSION** |  |  |  |  |
| Interpretation | 20 | Interpretation of the results, taking into account study hypotheses, | ✔ | 33,37 |
|  |  | sources of potential bias, imprecision of measures, multiplicative analyses, |  |  |
|  |  | and other limitations or weaknesses of the study |  |  |
|  |  | Discussion of results taking into account the mechanism by which the | ✔ | 33-35 |
|  |  | intervention was intended to work (causal pathways) or alternative |  |  |
|  |  | mechanisms or explanations |  |  |
|  |  | Discussion of the success of and barriers to implementing the intervention, | ✔ | 36-37 |
|  |  | fidelity of implementation |  |  |
|  |  | Discussion of research, programmatic, or policy implications | ✔ | 38 |
| Generalizability | 21 | Generalizability (external validity) of the trial findings, taking into account | ✔ | 38 |
|  |  | the study population, the characteristics of the intervention, length of |  |  |
|  |  | follow-up, incentives, compliance rates, specific sites/settings involved in |  |  |
|  |  | the study, and other contextual issues |  |  |
| Overall | 22 | General interpretation of the results in the context of current evidence | ✔ | 38 |
| Evidence |  | and current theory |  |  |


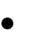

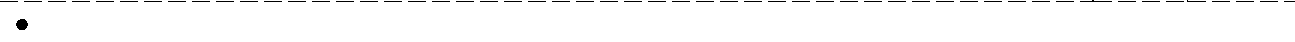

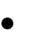

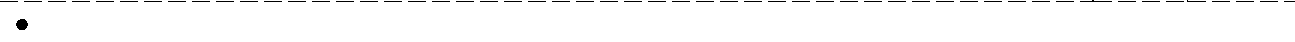

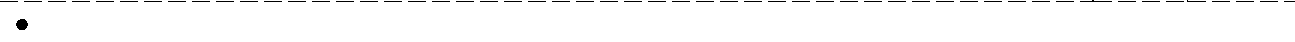

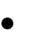

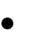

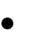

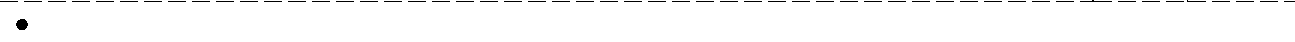

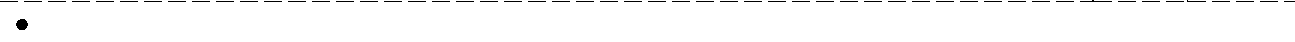

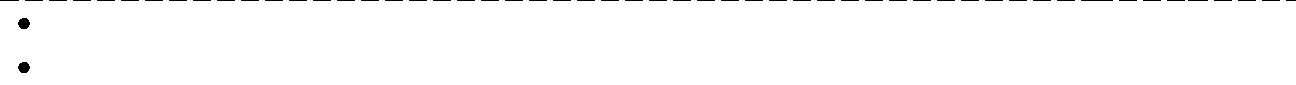

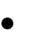

Supplement: S2 File — (DOCX) [file pone.0229755.s002.docx]
