# Supplementary material for: Effectiveness of a culturally appropriate nutrition educational intervention delivered through health services to improve growth and complementary feeding of infants: A quasi-experimental study from Chandigarh, India
Source: PLoS One. 2020 Mar 17;15(3):e0229755. doi: 10.1371/journal.pone.0229755 (PMC7077818; doi:10.1371/journal.pone.0229755)
Supplement: S3 File — (DOCX) [file pone.0229755.s003.docx]

**S3 File. Informed Consent Form**

**Study Title:** Effectiveness of a culturally appropriate nutrition educational intervention delivered through health services to improve growth and complementary feeding of infants: A quasi experimental study in Chandigarh, India.

**Name of the Institute:** Post Graduate Institute of Medical Education and Research (PGIMER), Chandigarh.

Subject’s Initials: _______________

Subject’s Name:_______________

Date of Birth / Age: _________________

|  | Participant's initial |
| --- | --- |
| 1. I confirm that I have read and understood the information sheet dated _ _ _ for the above study and have had the opportunity to ask questions. |  |
| 2. I understand that my participation in the study is voluntary and that I am free to withdraw at any time, without giving any reason, without my medical care or legal rights being affected |  |
| 3. I understand that the Sponsor of the clinical trial, others working on the Sponsor’s behalf, the Ethics Committee and the regulatory authorities will not need my permission to look at my health records both in respect of the current study and any further research that may be conducted in relation to it, even if I withdraw from the trial. I agree to this access. However, I understand that my identity will not be revealed in any information released to third parties or published |  |
| 4. I agree not to restrict the use of any data or results that arise from this study provided such a use is only for scientific purpose(s). |  |
| 5. I agree to take part in the above study. |  |

Signature (or Thumb impression) of the Subject:_____________

Date: _____/_____/______

Signatory’s Name: ________________________________________

Signature of the Investigator: ____________________________

Date:_____/_____/______

Study Investigator’s Name: ____________________________________
