# Supplementary material for: Effectiveness of a culturally appropriate nutrition educational intervention delivered through health services to improve growth and complementary feeding of infants: A quasi-experimental study from Chandigarh, India
Source: PLoS One. 2020 Mar 17;15(3):e0229755. doi: 10.1371/journal.pone.0229755 (PMC7077818; doi:10.1371/journal.pone.0229755)
Supplement: S4 File — (DOCX) [file pone.0229755.s004.docx]

**S4 File.** **Participant Information Sheet**

**Study Title:** Effectiveness of a culturally appropriate nutrition educational intervention delivered through health services to improve growth and complementary feeding of infants: A quasi experimental study in Chandigarh, India.

**Name of the Institute:** Post Graduate Institute of Medical Education and Research (PGIMER), Chandigarh.

Dear Parent/Guardian/Participant,

We would like to invite you and/or your child to participate in the research study. You are free to choose to either participate or withdraw from the study.

**Name of the participants**

Mother/Caregiver/Participant …………………..…

Child …………………………

**Purpose of the study**

This study is being done to to assess the effectiveness of culturally appropriate nutrition educational intervention delivered through health services to improve growth and complementary feeding by infants of age 6 months to 1 year old in Chandigarh.

We will ask you some basic questions regarding you and your child and household.

Please take the time to read or to listen as I read the following information. The information in this document is meant to help you decide whether or not to take part in this study. You may talk to others about the study if you wish. Participation in this study is completely voluntary. You may withdraw from this study at any time. Please ask me if there is anything that is not clear, or if you would like more information. When all of your questions have been answered and you understand the study, we will ask for your permission to allow you to take part in the study and to sign this consent form.

**Procedures that will be followed during this study**

If you agree to take part in the study, you will complete an interview with our interviewer who will ask you basic questions. The interview will take some of your valuable time. During this interview, some basic questions will be asked regarding you and your child and household. A routine physical and anthropometric measurement will be done after the interview.

**Benefits and Risks**

It may help to improve the feeding habits of your child and improve his nutritional status. No possible risk.

**Compensation**

We would not be able to provide any compensation to anyone who participates in thisstudy.

**Confidentiality**

Your participation will be kept confidential. Your name will not be revealed to anyone outside of the study team. Your answers will be treated with confidentiality and will be revealed only to the doctors/researchers involved in this study. The result of this study may be published in a report/scientific journal but your name will not be identified.

**Voluntary participation/ withdrawal and right to refuse or withdraw from the study**

Your participation in the research study is completely voluntary. This means that you can choose not to take part in this study. If you agree to be interviewed, you can refuse to respond to any question. You can also stop the interview at any time.

**Questions**

We have used some technical terms in this form. Please feel free to ask about anything you don’t understand and to consider this research and the consent form carefully before you make a decision.

**Cost to the participant**

You will not be paid to participate in the study.

**Right to new information**

If the research team gets any new information during the research study that may affect your decision to continue participating in the study, or may raise some doubts, you will be told about that information.

**Contact information**

For further information/questions, you may contact us at the following address:

1. Dr. Nikita Sharma, Department of Community Medicine and School of Public Health, PGIMER, Chandigarh. Phone no: xxxxxxx.
2. Dr. Madhu Gupta, Professor, Department of Community Medicine and School of Public Health, PGIMER, Chandigarh. Phone no: xxxxxxx.

In case of conflits, you can contact the chairperson of our institutional ethics committee at the following address:

Chairperson/Convener, Institutes Ethics Committee, PGIMER, Chandigarh

Telephone: xxxxxxx
