# Supplementary material for: Effectiveness of a culturally appropriate nutrition educational intervention delivered through health services to improve growth and complementary feeding of infants: A quasi-experimental study from Chandigarh, India
Source: PLoS One. 2020 Mar 17;15(3):e0229755. doi: 10.1371/journal.pone.0229755 (PMC7077818; doi:10.1371/journal.pone.0229755)
Supplement: S5 File — (DOCX) [file pone.0229755.s005.docx]

**S5 File. Focus Group Discussion Guide for Mothers/ Caregivers.**

**Title**

Effectiveness of a culturally appropriate nutrition educational intervention delivered through health services to improve growth and complementary feeding of infants: A quasi experimental study in Chandigarh, India.

**Name of the Institute:** Post Graduate Institute of Medical Education and Research (PGIMER), Chandigarh.

**Estimated time:** 90 minutes at maximum

**Objectives**

1. To explore current practice, barriers, facilitators on complementary feeding (CF) from perspectives of mothers/ ANMs

2. To explore the culturally appropriate foods and foods that is available to families in your area.

3. To explore the knowledge and attitude of mothers/ANM regarding complementary feeding.

4. To identify the role/influence of the family members, especially grandmother and husband on decision making of the mother to give appropriate complementary foods.

5. To identify potential communication channels for infant and young child feeding.

**Subjects**

A group of 8-10 mothers/ caregivers / 3-4 ANMs/ 2-3 community representatives will be conveniently selected from the community. Age group difference, economic conditions (poor and non-poor) and different children groups (6-8 months and 9-11 months) will be considered to select purposive participants. In total, 2 FGDs with the participants will be done.

Materials: Pieces of colored paper, different colored pens, audio recorders/ video recorders.

**Methods**

The focus group discussion will be facilitated by the researcher who is the junior resident and a note-taker. The discussion will be audio recorded/video-recorded if participants agree to do so. Free listing and ranking will be applied to identify the common benefits, disadvantages, roles of family members etc. The following topics will be explored:

- Culturally appropriate foods and foods that is available to families in your area.
- gap between knowledge and action of mothers
- frequency of feeding etc.

**FGD for mother included the following discussion points**

1. At what age should the mother start to feed her child foods other than breastmilk? Why?
2. What are the current feeding practices of your child?
3. What are the culturally appropriate foods available in your area?
4. Can you tell us what foods are appropriate to give children 6-12 months? And why?

PROBE: any foods including 1) cereals (rice), 2) meat, fish, egg, 3) fruits (banana, mango), 4) vegetable [*saag* (spinach), tomato, *gobi* (cauliflower)], 5) liquids like (tea, milk)

1. Is there any difference between what you would give to children 6-8 months old and what you would give to children 9-12 months old? [example: *daal ka pani* (gravy) for the former and *khichadi* (rice and pulses mix), *dalia* (porridge) for the latter]
2. What foods are NOT good/appropriate for children 6-12 months? And why? (not good taste, take time to prepare, cook, feed the child…….)
3. What is the support you have to feed your child?

(PROBE: preparing, cooking, feeding the child, encourage the child to eat)

1. Who else in the family supports the mother to feed her child? (FREE LISTING then RANKING who is the most supportive (PROBE: the father, the grandfather, cousin, neighbour)

**FGD for health workers included the following discussion points**

1. What are the factors that contribute to under nutrition among children in this community?
2. What are the current practices, barriers, facilitators and influencers complementary feeding (CF) in the community ?
3. At what age should the mother start to feed her child foods other than breastmilk? Why?
4. What are the culturally appropriate foods available in your area?
5. Can you tell us what foods are good / culturally appropriate to give children 6-12 months? And why?
6. Is there any difference between what you would give to children 6-8 months old and what you would give to children 9-12 months old?
7. What foods are NOT good/appropriate for children 6-12 months? And why?
8. How do you support the mother to feed her child? What more can you do to support the mother? FREE LISTING (PROBE: preparing, cooking, instructing, demonstrating the mother to feed the child, feeding the child, encourage the child to eat)
9. How can you improve the complementary feeding practices in the community?

Also, after an issue has been discussed, a summarization will be done to ensure that all opinions surrounding the issue have been covered.
