# Supplementary material for: Effectiveness of a culturally appropriate nutrition educational intervention delivered through health services to improve growth and complementary feeding of infants: A quasi-experimental study from Chandigarh, India
Source: PLoS One. 2020 Mar 17;15(3):e0229755. doi: 10.1371/journal.pone.0229755 (PMC7077818; doi:10.1371/journal.pone.0229755)
Supplement: S6 File — (DOCX) [file pone.0229755.s006.docx]

**S6 File. Questionnaire for Mother-Infant Dyad**

**Title:** Effectiveness of a culturally appropriate nutrition educational intervention delivered through health services to improve growth and complementary feeding of infants: A quasi experimental study in Chandigarh, India.

**Name of the Institute:** Post Graduate Institute of Medical Education and Research (PGIMER), Chandigarh.

**I. Basic information of mother/ caregiver:**

1. Unique ID no. …………………………
2. Name - ……………………………..
3. Age(years) - ………………………………
4. Sex: 1= M, 2=F
5. Address - ……………………………………………………..
6. Area: 1. Urban 2. Rural 3. Slum
7. Phone no. ……………………………….
8. Religion- 1. Hindu 2. Muslim 3. Sikh 4 Christian 5. Others.
9. Caste – 1.Gen 2. SC 3. ST 4. OBC 5. others
10. Education- 1/2/3/4/5/6/7
11. Occupation - 1/2/3/4/5
12. Monthly income of the mother:…………..
13. Marital status – 1/2/3/4/5/6
14. If mother is working who will take care of the baby ………….( 1 = in laws, 2 = crèche, 3 = others specify …………………. 4.By self )
15. Type of family- 1. Nuclear family 2. Joint family 3. Three generation family 4. Broken family 5. Other specify………

Family profile –

| Serial no. | Name | Age | Sex | Relationship with the head | Education | Occupation | Monthly income |
| --- | --- | --- | --- | --- | --- | --- | --- |
|  |  |  |  |  |  |  |  |
|  |  |  |  |  |  |  |  |
|  |  |  |  |  |  |  |  |
|  |  |  |  |  |  |  |  |
|  |  |  |  |  |  |  |  |
|  |  |  |  |  |  |  |  |
|  |  |  |  |  |  |  |  |

1. **Relation to family member-** self-1, father-2, mother-3, sister-4, brother-5, grand (father-mother)-6, uncle & aunt-7, other-8
2. **Gender-** Male- 1, Female*-* 2
3. **Education-** illiterate-1, primary school certificate- 2, middle school certificate - 3, High school certificate - 4, Intermediate or post high school diploma-5, Graduate or post graduate - 6, Professional or Honours- 7
4. **Occupation -** 1. Working (Govt./Private) 2. Homemaker 3.selfemployed
    4. Labourer/ maid 5. Student
5. **Marital status-** unmarried-1, married-2, widow-3, separated-4, divorce-5, married but not gauna-6
6. Total income of the family-…………..
7. Kuppuswamy SES score:……………
8. Socioeconomic status (According to modified Kuppuswamy Scale, 2014) – 1.Upper class 2. Upper middle class 3. Lower middle class 4. Upper lower class 5. Lower class
9. Food security score:………….(1= always enough food to eat, 2 = sometimes not enough food to eat, 3 = often not enough to eat)

**II. Child Information sheet**

1. Unique ID no of the child:
2. Name - …………………….
3. Father’s Name - ………………………….
4. D.O.B. - …………………(dd/mm/yy)
5. If date of birth not available age of the baby as per caregiver:………(dd/mm/yy)
6. Completed age at enrolment - …………..months …………days
7. Sex – ……….(1 = M 2 = F)
8. Was the child born at …………( 1= hospital, 2= home, 3=other)
9. Order of birth – 1 / 2 / 3 / 4 / 5 / 6 and above.
10. Birth weight (in Kg) - …………………….
11. Birth:………………(1= Term, 2= Preterm…….Weeks)
12. Immunization status –

| Sr. No. | Vaccine | date of administration of vaccine | age at which vaccine administered | Status- (1= Received/2=not received) |
| --- | --- | --- | --- | --- |
|  | Date of Birth |  |  |  |
| 32. | BCG |  |  |  |
| 33. | OPV-0 |  |  |  |
| 34. | Hep B -0 |  |  |  |
| 35. | DPT1/ Penta1 |  |  |  |
| 36. | DPT2/Penta2 |  |  |  |
| 37. | DPT3/Penta3 |  |  |  |
| 38. | IPV |  |  |  |
| 39. | Rotavirus vaccine |  |  |  |
| 40. | OPV-1 |  |  |  |
| 41. | OPV-2 |  |  |  |
| 42. | OPV-3 |  |  |  |
| 43. | Measles |  |  |  |
| 44. | Vitamin A |  |  |  |

45. Present weight (Kg) -…………………..

1. Present length(cm) - ………………..
2. Malnutrition status: ……………(1= Normal, 2= Moderate Malnutrition, 3= SAM)
3. Wt/age : ………..
4. Wt/ht : ………….
5. Ht/age: ………….

**III. Housing situation:**

1. Living in a rented accommodation: .…{1= Yes , 2 = No (own house), 3= (No, others…..)}
2. Type of house: …………(1=*kutcha*, 2 = *pucca,* 3 = *kutcha-pucca*)
3. No. of rooms ……..(1= 1 room, 2 = 2 rooms, 3 = 3 rooms, 4 = 4 rooms)
4. Overcrowding at home: …..(1= yes, 2 = no)
5. Safe water supply: ………. 1.yes 2.no
6. Main type of cooking fuel used : ……..1. LPG 2. Kerosene stove 3. *Chulha*
7. Sanitary latrine :……….1=yes, 2=no
8. If yes: 1= own toilet, 2 = shared toilet If yes: 1= own toilet, 2 = shared toilet

**IV. Maternal knowledge regarding breastfeeding and complementary feeding practices**

1. Which milk is best for the baby?

1.Breast milk 2.Formula milk 3.Animal milk 4. Don’t know

1. Up to which month exclusive breast feeding should be given?

1. Up to 6 months 2.4-6 months 3.7-8 months 4.Don’t know (998)

1. Can bottle feeding be given to baby? 1.Yes 2.No 3.Don’t know(998)
2. At what age complementary foods (solid, semisolid or soft foods) should be introduced? 1.4-6 months 2.At 6 months 3.7-8 months 4. Don’t know(998)}
3. Name three complementary foods that you think are good for a6-9 month old baby? ………………………………………………………………..(1.Know 2.Don’t Know(998))

(Note: If respondent answer any 3 food items then select option1.Eg: Khichidi, sujikheer, mashed fruits and vegetable, roti, mashed dhal, rice with dal, dhaliya with milk, basen or attacheera etc. If respondent say fruit juice, dhal water, milk then select option 2)

1. How many times a day solid/semi-solid foods should be given to 6-8 months baby? 1.= 3or more times 2. 1-2 times 3.Don’t know (998)
2. What should be the consistency of food given to baby? 1. Thick 2.Thin 3.Very thin
3. Name three snacks that can be given to a 6-8 month old baby? ……………………………………………………(1.Know 2.Don’t Know(998)) (Note: If respondent answer any 3 food items then select option1.Eg: fruits- banana, apple, papaya, mango,chiku,orangeetc, murmura, biscuits. If respondent say kurkure, chocolate, chips, fruity then select option 2)
4. What is the quantity of solid/semi-solid foods your child can consume at his/her age per feed?

| 1. 1-2 teapoonfuls | 1. 1/2 of a 250 ml cup |
| --- | --- |
| 1. 3-5 teapoonfuls | 1. 3/4^th^ of a 250 ml cup |
| 1. 2-3 tablespoonfuls | 1. Full cup |
| 1. 4-5 tablespoonfuls |  |

1. Can tea / sugar drinks/ coffee can be given to your child? 1.Yes 2. No
2. Should ghee or oil be used in complementary foods? 1.Yes 2. No 3.Don’t know (998)
3. Do you know till what age breastfeeding can be continued?
    1.Less than 1 year 2. 1 year - < 2 years 3.Upto& beyond 2 years 4. Don’t know (998)
4. Are commercial baby foods better for infants than homemade foods? 1.Yes 2.No 3.Don’t know (998)
5. Do you need to wash hands with soap and water before feeding the child? 1.yes 2. no
6. If your child is not taking food, should he/she be threatened or bribed?
    1. Yes 2. No 3. Sometimes

**V. Feeding Practices**

***Past***

1. When was breastfeeding initiated after birth?

1.Within 1 hour 2. 1-4 hours 3.4-24 hours 4.After 24 hours 5.Not Given

1. Which food was given first after birth (Pre lacteal feed)?

1.Breast milk 2.Animal milk 3.Standard formula 4.Ghutti 5..Honey 6.Others

1. Did you feed colostrum to the child? 1.Yes 2.No
2. Did your baby receive exclusive breastfeeding (EBF) till 6 months of age? 1.Yes 2.No 3.Baby <6 months of age but on EBF 4.Baby < 6 months but not EBF
3. If No, reason 1.Decreased milk secretion after birth 2. Mother not feeling well/ tired 3.Working mother 4.Baby not satisfied/hungry 5. Prelacteal feed
4. Did your baby receive bottle feeding at any time before 6 months of age? 1.Yes 2. No

***Current***

1. What is the current feeding practice?

1.Only breastfeeding 2.Breast milk + animal milk or formula milk
 3.Breastfeeding+ Complementary feeding 4. Completely weaned

If child below 6 months & answer is option 1 or 2, then skip to question no. 94

1. At what age did you started giving your child solid/semi-solid foods?

1.< 6 months 2. At 6 months 3.7-9 months 4.10 months and above
 5.Not started

1. Type of complementary food being fed to the baby?

1.Cow/Buffalo milk 2.Commercial baby food 3.Homemade semi-solid/solid food 4.Commercial baby food + homemade food 5. Homemade semi-solid/solid food + Cow/Buffalo milk 6. Cow/Buffalo milk + Commercial baby food

7. Homemade semi-solid/solid food + Cow/Buffalo milk + Commercial baby food

1. Is your baby currently receiving bottle feeding? 1.Yes 2. No
2. Is your child eating thick food first at the main meal? 1.Yes 2. No
3. How many times in past 24 hours, your child has taken solid/semi-solid foods? ( 0/ 1 / 2 / 3 / 4 / 5 )
4. What is the quantity of solid/semi-solid foods your child can consume at his/her age per feed?

| - 1. 2-3 tablespoonfuls | - 1. 3-4 tablespoonfuls |
| --- | --- |
| - 1. 4-5 tablespoonfuls | - 1. 2/3^rd^ of a 250 ml cup |
| - 1. 3/4^th^ of a 250 ml cup | - 1. Full cup |
| - 1. Full cup |  |

1. What is the mode of feeding the child? 1.By spoon 2.By hand 3.Both 4.Self by hand/spoon
2. What is consistency of feeds being received by your child? 1.Thick 2.Thin 3.Very thin
3. Are you adding ghee or oil in complementary foods? 1.Yes 2.No
4. Average food group taken daily in last 7 days except milk : 1 / 2 / 3 / 4 / 5

| Food groups | Every day | Weekly thrice | Weekly twice | Never |
| --- | --- | --- | --- | --- |
| **Cereals & Starches, tubers** -Rice, Bread *atta*, *suji, dhaliya*, corn flakes, *Poha* |  |  |  |  |
| Potato, carrot, sweet potato |  |  |  |  |
| **Commercial products** - cerelac, farex |  |  |  |  |
| **Commercial infant formula-** lactogen, nestogen, Nan |  |  |  |  |
| **Pulses- nuts, legumes**,  Any dhal, lentils, Peanuts, Almonds, Cashew nuts, *besan*, Beans |  |  |  |  |
| **Dairy products:** milk, curds, paneer |  |  |  |  |
| Green leafy vegetables-*palak, methi*. |  |  |  |  |
| **Other Vegetable and Fruits:** pumpkin, bottle guard, cabbage |  |  |  |  |
| Fruits - banana, apple, papaya, orange |  |  |  |  |
| **Meat/fish/chicken** |  |  |  |  |
| **Egg** |  |  |  |  |
| **Oil/ghee/butter** |  |  |  |  |
| Miscellaneous: Sugar/ jaggary |  |  |  |  |
| Biscutes |  |  |  |  |
| sawary snacks |  |  |  |  |
| carbonated drinks |  |  |  |  |
| Tea |  |  |  |  |
| Tetra packet juices(Fruity or maza) |  |  |  |  |
| Maggi noodles, macoroni, pasta |  |  |  |  |
| junk food *kurrkurre*, chips, pizza,burger |  |  |  |  |
| Chocolates or sweets |  |  |  |  |

1. Are you feeding tea/ sugar drinks to your child? 1.Yes 2. no
2. Is your child receiving any snacks in between meals? 1.Yes 2. No
3. Do you wash the child’s hands with soap before feeding? 1.Yes 2.No
4. Do you wash your hands with soap and water before feeding the child? 1.Yes 2. No
5. Do you maintain eye contact and talk to your child while feeding him/her? 1.Yes 2.No
6. Do you encourage your child to take feed by praising him/her?1.Yes 2. No

|  | Item | Quantity | Calories |
| --- | --- | --- | --- |
| Early morning |  |  |  |
| Breakfast |  |  |  |
| Midmorning |  |  |  |
| Lunch |  |  |  |
| Post lunch |  |  |  |
| Evening |  |  |  |
| Dinner |  |  |  |

1. Total Calories Consumed-……………. (1=Deficit 2= Excess)……………
2. Cumulative Score for Infant and young child Feeding index (CS-ICFI)……………….
3. Signature of the investigator: ……………….
4. Name of the investigator: ……………….
