# Supplementary material for: Effectiveness of a culturally appropriate nutrition educational intervention delivered through health services to improve growth and complementary feeding of infants: A quasi-experimental study from Chandigarh, India
Source: PLoS One. 2020 Mar 17;15(3):e0229755. doi: 10.1371/journal.pone.0229755 (PMC7077818; doi:10.1371/journal.pone.0229755)
Supplement: S7 File — (DOCX) [file pone.0229755.s007.docx]

**S7 File. Questionnaire For Health Workers**

**Title:** Effectiveness of a culturally appropriate nutrition educational intervention delivered through health services to improve growth and complementary feeding of infants: A quasi experimental study in Chandigarh, India.

**Name of the Institute:** Post Graduate Institute of Medical Education and Research (PGIMER), Chandigarh.

**Basic information of ANM:**

1. ID No.
2. Name - ……………………….
3. Age - ………………………………
4. Sex – 1= M, 2= F
5. Address - ……………………………………………………..
6. Phone no. ……………………………….
7. Religion………1. Hindu 2. Muslim 3. Sikh 4.Christian 5.Others
8. Education- ………… (lliterate-1, primary school certificate- 2, middle school certificate - 3, High school certificate - 4, Intermediate or post high school diploma-5, Graduate or post graduate - 6, Professional or Honours- 7)
9. Employment status - 1.Regular 2.contract.
10. Years of service as ANM: 1. 1-3years 2. 4-6 years 3. 7-9 years 4. 10 years or more
11. **Knowledge of ANMs regarding Infant and Young Child Feeding (IYCF) practices**
12. When will breastfeeding be initiated after birth?

1.Immediately, within 1 hour 2.>1 hour-4 hours 3.5-24 hours 4.After 24 hours.

1. Mother’s first milk (colostrum) should be given to newborn baby?

1.Yes 2.No

1. Do you know what is exclusive breast feeding? (Choose one option)

a. Breast milk only b. Breast milk and water c. Breast milk and/or ORS/Vitamin supplements/Medicines)

(1.Yes 2. No)

1. Can bottle feeding be given to the child? 1.Yes 2. No 3. Don’t know
2. At what age complementary foods (solid, semisolid or soft foods) should be introduced? ………………………………… {1.correct 2. Incorrect 3. Don’t know}
3. Name three complementary foods that you think are best for an infant 6-8 months old? 1.……………………2……………………3…………………(1.Know 2.Don’t Know)
4. How many times a day solid/semi-solid foods should be given to 6-8 month old infant? 1. Once a day 2. 2-3 times 3. 4-5 times 4.Don’t know
5. What should be the consistency of complementary foods given to the infant? 1.Thick 2.Thin 3.Very thin
6. Name three snacks that can be given to a 6-8 month old infant? 1…………..………2………….………3…………………(1.correct 2. incorrect)
7. What is the quantity of solid/semi-solid foods a 6-8month infant can consume at his/her age per feed?
8. Start with 1-2 teaspoons and increase to 1/4^th^ of a 250 ml cup
9. Start with 2-3 tablespoons and increase to 1/2 of a 250 ml cup
10. Start with 4-5 tablespoons and increase to 3/4^th^of a 250 ml cup
11. Full cup
12. Can tea / sugar drinks can be given to the infant? 1.Yes 2. No
13. What is the minimum no. of food groups that should be given to a 6-8 month infant?

(1) Grains, roots and tubers, legumes and nuts;

(2) dairy products ;

(3) flesh foods (meat, fish, poultry);

(4) eggs,

(5) vitamin A rich fruits and vegetables;

(6) other fruits and vegetables

a. 2-3 food groups b. 4 or more food groups (1. Know 2. Don’t know)

1. Do you know till what age breastfeeding can be continued? …………………………………. (1. Know 2.Don’t know)
2. Can ghee/oil/butter be added to the food of infants? 1.Yes 2. No
3. Do you have knowledge about responsive feeding? 1.Yes 2.No
4. **Practice of ANM regarding IYCF practices**
5. Do you advice mothers to wash their hands with soap and water before feeding infant? 1.Yes 2. No
6. Do you advice mother to wash the infant’s hands before feeding? 1.Yes 2. No
7. Do you advice mother to maintain eye contact and talk to infant while feeding him/her? 1.Yes 2. No
8. Do you tell the mothers to add ghee/oil/butter to the food of infants? 1.Yes 2. No
9. Do you tell the mothers to feed snacks to the infants in between meals? 1.Yes 2. No

| Does this practice occur? | 1.With all children | 2.With some children | 3.Does not occur | 4.Others (comment) |
| --- | --- | --- | --- | --- |
| 31.Weigh child during routine immunization |  |  |  |  |
| 32. Measure Child Growth during routine immunization |  |  |  |  |
| 33. Discuss how the child is feeding during routine immunization |  |  |  |  |
| 34. Group counseling on IYCF during routine immunization sessions |  |  |  |  |
| 35. Carry out demonstrations of young children’s food preparations and feeding techniques during VHND |  |  |  |  |
| 36. Make home visits to assess foods and feeding practices |  |  |  |  |
