# Supplementary material for: Effectiveness of a culturally appropriate nutrition educational intervention delivered through health services to improve growth and complementary feeding of infants: A quasi-experimental study from Chandigarh, India
Source: PLoS One. 2020 Mar 17;15(3):e0229755. doi: 10.1371/journal.pone.0229755 (PMC7077818; doi:10.1371/journal.pone.0229755)
Supplement: S8 File — (DOCX) [file pone.0229755.s008.docx]

**S8 File.** **Training Modules For Health Workers**

**Study Title:** Effectiveness of a culturally appropriate nutrition educational intervention delivered through health services to improve growth and complementary feeding of infants: A quasi experimental study in Chandigarh, India.

**Name of the Institute:** Post Graduate Institute of Medical Education and Research (PGIMER), Chandigarh.

**Session 1 –Introduction & orientation**

*1. Importance of complementary feeding*

In this session we will:

- define the term complementary feeding;
- discuss the optimal age for children to start complementary feeding;
- discuss the importance of continuing breastfeeding;
- examine the role of health worker and health facility; and
- list the key messages to discuss with caregivers about when to start complementary foods.

Key Messages:

1. Breastfeeding for two years of age or longer helps a child to develop and grow strong and healthy.
2. Starting other foods in addition to breast milk at six months helps a child to grow well.

Most babies do not need complementary foods before six months of age. All babies older than six months of age should receive complementary foods.

*2. Foods to fill the energy gap*

In this session we will:

- discuss the local foods that can help fill this energy gap;

- - - And explain the importance of using foods of a thick consistency;
    - discuss ways to enrich foods; and
    - list the key message of how to fill this gap to discuss with caregivers. Key Message:
  1. Foods that are thick enough to stay in the spoon give more energy to the child.

1. *Foods to fill the iron and vitamin A gaps*

In this session we will:

- - - discuss the local foods that can fill the nutrient gaps for iron and vitamin A;
    - discuss the use of processed complementary foods;
    - discuss the fluid needs of the young child;
    - list the key messages of how to fill these gaps to discuss with caregivers

Key Messages:

- 1. Animal foods are speciallygood for children, to help them grow strong and lively.
  2. Peas, beans, lentils, and nuts and seeds are good for children.
  3. Dark green leaves and orange coloured fruit and vegetables help the child to have healthy eyes and fewer infections.

1. *Quantity, variety and frequency of feeding*

In this session we will discuss:

- how to use a mixture or variety of foods to help fill the gaps;
- how often to feed complementary foods;
- the quantity of complementary foods to offer, and
- the key messages to share with caregivers and your co- workers.

Key Messages:

1. A growing child needs three meals plus snacks: give a variety of foods.
2. A growing child needs increasing amounts of food.

| Age | Texture | Frequency | Amount at each meal |
| --- | --- | --- | --- |
| from 6 months | Soft porridge, well  Mashed vegetable, meat, fruit | two times per day plus  frequent breastfeeds | 2 to 3 tablespoonfuls |
| 7 to 8 months | Mashed foods | Three times per day plus frequent breastfeeds | increasing gradually to  2/3 of a 250 ml cup at  each meal |
| 9 to11 months | Finely chopped or  Mashed foods, and foods that baby canpick up | three meals plus one  snack between meals  plus breastfeeds | 3/4 of a 250 ml  cup/bowl |
| 12 to 24 months | Family foods, chopped or mashed if necessary | three meals plus two  snacks between meals  plus breastfeeds | A full 250 ml cup/bowl |

1. *Feeding During Illness and Recovery*

In this session we will look at:

- - - the importance of continuing to feed a child during illness;
  - ways of encouraging children to eat during illness and recovery; and counseling caregivers on appropriate feeding practices during illness.

Key Messages:

1. Encourage the child to drink and to eat during illness and provide extra food after illness to help them recover quickly.

**Session *2-* Communication Skills**

***1.*** *Listening and learning skills*

In this session we will discuss:

- How to use basic counseling skills to listen and learn from caregivers about their complementary practices. For example:

- 1. Use helpful non-verbal communication
  2. Ask open questions
  3. Use responses and gestures that show interest
  4. Reflect back what the caregiver says
  5. Empathize – show that you understand how she/he feels
  6. Avoid words that sound judging

1. *Building confidence skills*

In this session we will discuss:

- How to use basic counseling skills to build confidence and give support to caregivers about their complementary feeding practices.

1. Accept what a caregiver thinks and feels
2. Recognize and praise what a caregiver and child are doing right
3. Give practical help
4. Give a little relevant information
5. Use simple language
6. Make one or two suggestions, not commands

**Session 3*-* Home visit tasks and procedures**

***1.*** *Gathering Information on Complementary Feeding Practices*

In this session we will look at:

- - the importance of observation skills and observing interactions between caregivers and children;
  - using growth charts in assessment of complementary feeding practices;
  - how to gather information on complementary feeding practices using a Food Intake Tool.

1. *Feeding Techniques and Strategies*

In this session we will look at:

- - feeding practices and their effect on the child’s intake;
  - ways of encouraging responsive feeding practices;
- requirements for clean and safe feeding of young children; and
- how possible it is to carry out these recommendations.

f*ood Intakes and Counseling at each visit*

| Sr. No. | Caregivers practices | Practice in place | Informed/ Suggested |
| --- | --- | --- | --- |
| 1 | Child receives breast milk? |  |  |
| 2 | Child eats three meals of thick consistency yesterday? |  |  |
| 3 | Child ate an animal product yesterday (meat/fish/bird/eggs)? |  |  |
| 4 | Child ate a dairy product yesterday? |  |  |
| 5 | Child ate pulses or nuts yesterday? |  |  |
| 6 | Child ate a dark green or orange vegetable or orange fruit yesterday? |  |  |
| 7 | Child eats sufficient number of meals and snacks yesterday, for his/her age? |  |  |
| 8 | Quantity of food eaten at main meal yesterday appropriate for child’s age? |  |  |
| 9 | Caregiver assists the child at meals times? |  |  |
| 10 | Child takes any vitamin or mineral supplements? |  |  |
| 11 | Child ill and not eating? |  |  |
