# Supplementary material for: Effectiveness of a culturally appropriate nutrition educational intervention delivered through health services to improve growth and complementary feeding of infants: A quasi-experimental study from Chandigarh, India
Source: PLoS One. 2020 Mar 17;15(3):e0229755. doi: 10.1371/journal.pone.0229755 (PMC7077818; doi:10.1371/journal.pone.0229755)
Supplement: S9 File — (DOCX) [file pone.0229755.s009.docx]

**S9 File. Training Module for Mothers**

**Study Title:** Effectiveness of a culturally appropriate nutrition educational intervention delivered through health services to improve growth and complementary feeding of infants: A quasi experimental study in Chandigarh, India.

**Name of the Institute:** Post Graduate Institute of Medical Education and Research (PGIMER), Chandigarh.

1. **Key messages for complementary feeding**
   1. Breastfeeding for two years of age or longer helps a child to develop and grow strong and healthy.
   2. Starting other foods in addition to breast milk at six months helps a child to grow well.
   3. Foods that are thick enough to stay in the spoon give more energy to the child.
   4. Animal foods are especially good for children, to help them grow strong and lively.
   5. Peas, beans, lentils, and nuts and seeds, are good for children.
   6. Dark green leaves and orange colored fruits and vegetables help the child to have healthy eyes and fewer infections.
   7. A growing child needs three meals and snacks: give a variety of foods.
   8. A growing child needs increasing amounts of food.
   9. A young child needs to learn to eat: encourage and give help…with lots of patience.
   10. Encourage the child to drink and to eat during illness and provide extra food after illness to help them recover quickly.
2. **Amounts of foods to offer**

| Age | Texture | Frequency | Amount at each meal |
| --- | --- | --- | --- |
| from 6 months | Soft porridge, well  Mashed vegetable, meat, fruit | two times per day plus  frequent breastfeeds | 2 to 3 tablespoonfuls |
| 7 to 8 months | Mashed foods | Three times per day plus frequent breastfeeds | increasing gradually to  2/3 of a 250 ml cup at  each meal |
| 9 to11 months | Finely chopped or  Mashed foods, and foods that baby canpick up | three meals plus one  snack between meals  plus breastfeeds | 3/4 of a 250 ml  cup/bowl |
| 12 to 24 months | Family foods, chopped or mashed if necessary | three meals plus two  snacks between meals  plus breastfeeds | A full 250 ml cup/bowl |

**C.** **Five ways to keep safe foods**

1. Keep the hands clean

Wash your hands before handling food and often during food preparation.

Wash your hands after going to the toilet, changing the baby or in contact with animals. Wash very clean all surfaces and equipment used for food preparation or serving. Protect kitchen areas and food from insects, pests and other animals.

2. Separately keep the raw foods and cooked foods

Use separate equipment and utensils for handling raw meat and foods.

Store foods in separate covered containers.

3. Cook thoroughly

Cook food thoroughly, especially meat, poultry and eggs. Bring foods like soups and stews to boiling point.

For meat and poultry, make sure juices are clear not pink.

Reheat cooked food thoroughly. Bring to the boil or heat until too hot to touch. Stir while re-heating.

4. Keep cooked food at even and safe temperatures

Do not leave cooked food at room temperature for more than two hours. Do not store food too long, even in a refrigerator.

Do not thaw frozen food at room temperature.

Food for infants and young children should ideally be freshly prepared and not stored at all after cooking.

5. Use safe water and raw materials

Use safe water or treat it to make it safe. Choose fresh and wholesome foods.

Use pasteurized milk.

Wash fruits and vegetables in safe water, especially if eaten raw.
