# Supplementary material for: Effectiveness of a culturally appropriate nutrition educational intervention delivered through health services to improve growth and complementary feeding of infants: A quasi-experimental study from Chandigarh, India
Source: PLoS One. 2020 Mar 17;15(3):e0229755. doi: 10.1371/journal.pone.0229755 (PMC7077818; doi:10.1371/journal.pone.0229755)
Supplement: S11 File — (DOCX) [file pone.0229755.s011.docx]

**S11 File. CODE BOOK**

**Title: Effectiveness of a culturally appropriate nutrition educational intervention delivered through health services to improve growth and complementary feeding of 6 months to 1 year old infants in Chandigarh: a quasi experimental study.**

All endline variables start with **E_variable name** e.g. **E_Serial no., E_Best milk for baby etc.**

*** Missing children at endline** are coded as **9999.**

**I. Basic information of mother/ caregiver:**

1. **Serial no.**
2. **Group: 1. Intervention group 2. Control group**
3. **Timeline : 0. Baseline 1. Endline**
4. Age (years) - ………………………………
5. Sex: 1= M, 2=F
6. Area: 1. Rural 2. Peri-urban 3. Slum
7. Religion- 1. Hindu 2. Muslim 3. Sikh 4 Christian 5. Others.
8. Caste – 1.General 2.Schedule caste 3. Schedule tribe 4. Other backward caste 5. others
9. Education- 1/2/3/4/5/6/7

*(****Education-*** *illiterate-1, primary school certificate- 2, middle school certificate - 3, High school certificate - 4, Intermediate or post high school diploma-5, Graduate or post graduate - 6, Professional or Honours- 7)*

1. Occupation - 1/2/3/4/5

*(****Occupation -*** *1.Working (Government/Private job) 2. Homemaker 3.self-employed 4. Laborer/ maid 5. Student)*

1. Type of family- 1. Nuclear family 2. Joint family 3. Three generation family 4. Broken family 5. Other specify………
2. Socioeconomic status (According to modified Kuppuswamy Scale, 2017) – 1.Upper class 2. Upper middle class 3. Lower middle class 4. Upper lower class 5. Lower class

**II. Child Information sheet**

1. Enrolment age of infant - …………..months
2. Sex of infant – ……….(1 = M 2 = F)
3. Was the child born at (Birth Place)…………( 1= hospital, 2= home, 3=other)
4. Order of birth – 1 / 2 / 3 / 4 / 5 / 6 and above.
5. Birth weight (in Kg) - …………………….
6. Birth:………………(1= Term, 2= Preterm)
7. Weight (Kg) -…………………..
8. Length(cm) - ………………..
9. Malnutrition status: ……………(1= Normal, 2= Moderate Malnutrition, 3= SAM)
10. Weight for age : ………..
11. Weight for length: ………….
12. Length for age: ………….

**III. Housing situation:**

1. Type of house: …………(1=*kutcha*, 2 = *pucca,* 3 = *kutcha-pucca*)
2. Overcrowding at home: …..(1= yes, 2 = no)
3. Toilet : 1. Own toilet 2. Sharing toilet

**IV.Maternal knowledge regarding breastfeeding and complementary feeding practices**

1. Which milk is best for the baby?

1.Breast milk 2.Formula milk 3.Animal milk 998. Don’t know

1. Up to which month exclusive breast feeding should be given?

1. Up to 6 months 2.4-6 months 3.7-8 months 998.Don’t know

1. Can bottle feeding be given to baby?

1.Yes 2. No 998.Don’t know

1. At what age complementary foods (solid, semisolid or soft foods) should be introduced?

1.4-6 months 2.At 6 months 3.7-8 months 998. Don’t know

1. Name three complementary foods that you think are good for a 6-9 month old baby? (1.Know 2.Don’t Know(998))
2. How many times a day solid/semi-solid foods should be given to 6-8 months baby? 1.= 3or more times 2. 1-2 times 998.Don’t know
3. What should be the consistency of food given to baby? 1. Thick 2.Thin 3.Very thin
4. Name three snacks that can be given to a 6-8 month old baby? 1.Know 998.Don’t Know
5. Should ghee or oil be used in complementary foods? 1.Yes 2.No
    998.Don’t Know
6. Do you know till what age breastfeeding can be continued?
    1.Less than 1 year 2. 1 year - < 2 years 3.Upto& beyond 2 years
    998.Don’t Know
7. Are commercial baby foods better for infants than homemade foods? 1.Yes 2.No 998.Don’t know
8. Do you need to wash hands with soap and water before feeding the child? 1.yes 2. no
9. If your child is not taking food, should he/she be threatened or bribed?
    1. Yes 2. No 3. Sometimes

**IV.Feeding Practices**

***Past***

1. When was breastfeeding initiated after birth?

1.Within 1 hour 2. 1-4 hours 3.4-24 hours 4.After 24 hours 5.Not Given

1. Did your baby receive exclusive breastfeeding (EBF) till 6 months of age? 1.Yes 2.No 3.Baby <6 months of age but on EBF 4.Baby < 6 months but not EBF
2. Did your baby receive bottle feeding at any time before 6 months of age? 1.Yes 2. No

***Current***

1. What is the current feeding practice?
2. Only breastfeeding 2.Breast milk+animal milk or formula milk 3.Breastfeeding+ Complementary feeding 4. Completely weaned 999.Missing /not applicable
3. At what age did you started giving complementary foods (solid/semi-solid foods) to your child?
4. Less than 6 months 2. At 6 months 3.7-9 months 4.10 months and above 5.Not started 999.Missing/not applicable
5. Is your baby currently receiving bottle feeding? 1.Yes 2. No 999.Missing/not applicable
6. Is your child eating thick food first at the main meal? 1.Yes 2. No 999.Missing/not applicable
7. What is the mode of feeding the child? 1.By spoon 2.By hand 3.Both 4.Self by hand/spoon 999.Missing/not applicable
8. What is consistency of feeds being received by your child? 1.Thick 2.Thin 3.Very thin 999.Missing/not applicable
9. Average food group taken daily in last 7 days except milk : 1 / 2 / 3 / 4 / 5/ 999(Missing/not applicable)
10. Are you feeding tea/ sugar drinks to your child? 1.Yes 2. No 999.Missing/not applicable
